# Dividing time—An absolute chronological study of material culture from Early Iron Age urnfields in Denmark

**DOI:** 10.1371/journal.pone.0300649

**Published:** 2024-05-28

**Authors:** Helene Agerskov Rose, John Meadows

**Affiliations:** 1 Centre for Baltic and Scandinavian Archaeology, Foundation of Museums of the State of Schleswig-Holstein, Schleswig-Holstein, Germany; 2 Department of Historical Studies, University of Gothenburg, Gothenburg, Sweden; 3 Leibniz-Laboratory for AMS Dating and Stable Isotope Research, Kiel University, Schleswig-Holstein, Germany; University of California Santa Cruz, UNITED STATES

## Abstract

Chronological frameworks based on artefact typologies are essential for interpreting the archaeological record, but they inadvertently treat transitions between phases as abrupt events and disregard the temporality of transformation processes within and between individual phases. This study presents an absolute chronological investigation of a dynamic material culture from Early Iron Age urnfields in Denmark. The chronological framework of Early Iron Age in Southern Scandinavia is largely unconstrained by absolute dating, primarily due to it coinciding with the so-called ‘Hallstatt calibration plateau’ (c.750 to 400 cal BC), and it is difficult to correlate it with Central European chronologies due to a lack of imported artefacts. This study applies recent methodological advances in radiocarbon dating and Bayesian chronological modelling, specifically a statistical model for wood-age offsets in cremated bone and presents the first large-scale radiocarbon investigation of regional material culture from Early Iron Age in Southern Jutland, Denmark. Dated material is primarily cremated bone from 111 cremation burials from three urnfields. The study presents absolute date ranges for 16 types of pottery and 15 types of metalwork, which include most of the recognised metalwork types from the period. This provides new insights into gradual change in material culture, when certain artefact types were in production and primary use, how quickly types were taken up and later abandoned, and distinguishing periods of faster and slower change. The study also provides the first absolute chronology for the period, enabling correlation with chronologies from other regions. Urnfields were introduced at the Bronze-Iron Age transformation, which is often assumed to have occurred c.530-500 BC. We demonstrate that this transformation took place in the 7^th^ century BC, however, which revives the discussion of whether the final Bronze Age period VI should be interpreted as a transitional phase to the Iron Age.

## Introduction

**Fig 1:**
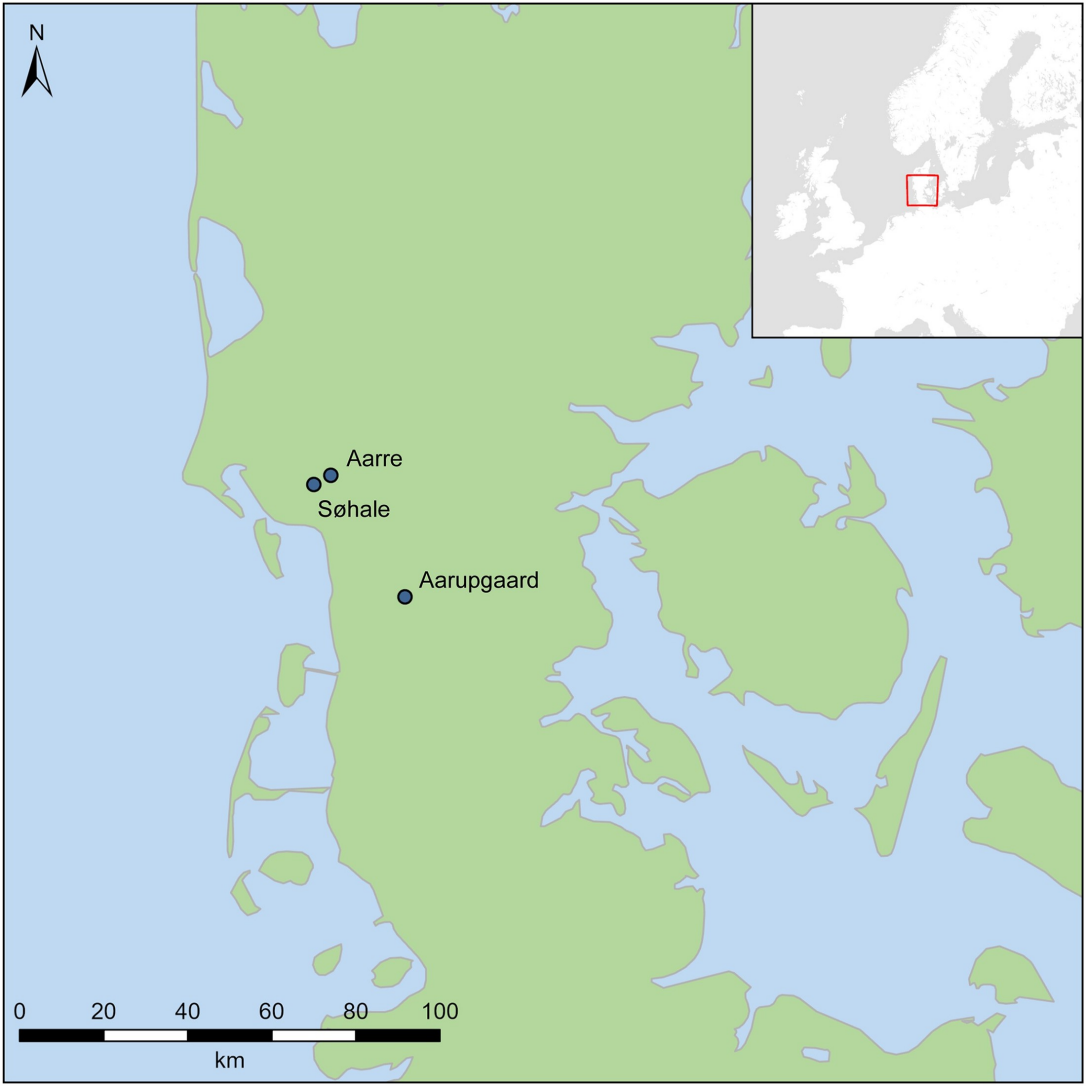
Location of Aarupgaard, Aarre and Søhale urnfields in south-west Jutland, Denmark. Map by R. Potter with Natural Earth (free vector and raster map data @ naturalearthdata.com).

Time and culture are old concepts within the discipline of archaeology, but how these are approached differs across the globe and has also changed over time, reflecting the emerging ‘schools of thought’. A common approach is the construction of chronological frameworks based on systematized observations of stratigraphy and typology, where burials have traditionally provided closed-context material for the typo-chronological analyses [e.g. 1]. This study investigates the absolute chronology of artefact assemblages from three urnfield cemeteries in Denmark (Fig 1). Concepts of period, culture and even geography have become ingrained into the discipline and it can be difficult to distinguish the terms ‘urnfield period’, ‘urnfield culture(s)’ and ‘urnfield area’ that are used interchangeably in the literature [2]. Here the term ‘urnfield’ are used only to describe the specific funerary practice present in northern Europe during the first millennium BC, without any assumptions regarding chronology or ethnicity.

**Fig 2:**
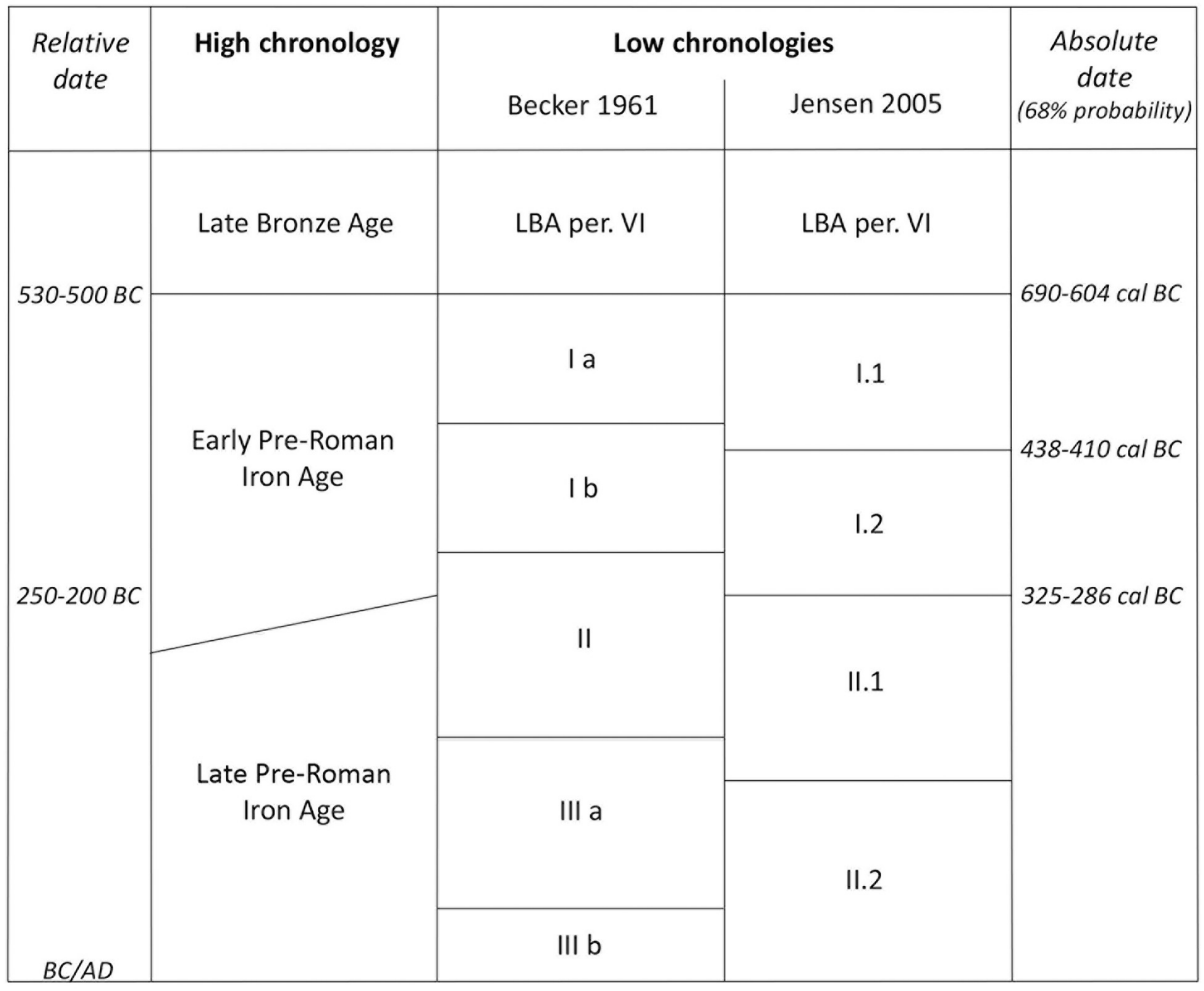
Chronological framework of the Pre-Roman Iron Age in Southern Scandinavia [1, 10]. Absolute dates at 68% probability are based on results presented in this paper.

The earlier part of the Iron Age in Southern Scandinavian, which is defined as ‘Pre-Roman’ (i.e., ‘before the Roman Empire’), began in c.500 BC [3]. The relative chronological framework of the Pre-Roman Iron Age (PRIA) is traditionally based on typo-chronological studies of urnfield artefact assemblages [1, 4–6], and presently two chronological systems are used in Danish archaeology: Becker’s from 1961 and Jensen’s from 2005 (Fig 2). Becker divided the PRIA into three periods, corresponding to Central European La Tène periods I-III [1], but Jensen demonstrated how this produced an uneven distribution of pottery and metalwork over the period, along with difficulties in harmonizing the materials, possibly because of divergent chronological sensitivities [7, 8]. Metalwork typology has been assumed to be chronologically sensitive, with more rapidly changing typological traits, whereas pottery types are considered to be more ‘conservative’ and longer-lasting [9]. Jensen [10] revised the chronological framework, specifically avoiding the use of ‘type fossils’ and divided the PRIA into two main chronological periods: Early PRIA (c.500-250 BC) and Late PRIA (c.250 BC–AD 1) [11], with further divisions into sub-periods and phases specific to the geographical area.

It is difficult to correlate the relative chronologies of Southern Scandinavia and Central European due to a lack of imported artefacts, but the Early Pre-Roman Iron Age has been suggested to coincide with Ha D in Central Europe, based on a few Wendel rings and eyelet rings from depot finds and two North European Certosa-type fibulae from a flat grave [12–14]. The chronological framework of Southern Scandinavia is largely unconstrained by absolute dating [although see 15]. This is partly due to difficulties associating features dated by radiocarbon (^14^C) or dendrochronology with typo-chronological phases, but primarily due to the longstanding perception that between c.750 and 400 cal BC the ^14^C calibration curve is too flat to justify ^14^C dating [16]. It is only with relatively recent developments in ^14^C dating techniques that it has become possible to date the bio-apatite of fully calcined bone [17], which is of great importance for research into the Late Bronze Age-Early Iron Age in north-western Europe when cremation was the dominant burial rite [e.g. 18]. Experimental studies have however shown that significant carbon exchange occurs between bone-apatite and the pyre atmosphere during cremation, which can cause a calendar date offset between the ^14^C event and the date of cremation [19–22]. If the effects of wood-age offsets on ^14^C dates from cremated bone are corrected with a statistical outlier model [22], it will date the cremation event and thus the deposition date of the associated artefacts. From an archaeological perspective, the cremation is a separate event occurring after death and before burial, but these events cannot have been separated by more than a few days or weeks. The production date of an artefact must have occurred sometime before deposition, and for the present purpose, it is assumed that part of the metalwork might have a discernible residence time, but that most of the funerary urns were made less than a decade before the cremation.

### Dividing time and identifying change

How archaeology as a discipline divides time and approaches chronology is largely defined by principles arising from cultural history that assume similarities and differences in material culture can be employed to define discrete and relatively homogenous social entities [23]. Cultural history was critiqued by New Archaeology for being descriptive rather than explaining human beliefs and behaviours [24, 25], but the methodological basis for placing research in space and time remained. This approach privileges certain research questions and has a great influence on how we write archaeological narratives [26].

Sequential ordering of archaeological material into relative typological chronologies or typo-chronologies is a vital part of the cultural history methodology [27, 28], and archaeologists have since the 19th century strived to construct increasingly detailed chronologies [e.g. 29–31]. Such frameworks have inherent linear and successive structures and assume change occurs at a constant rate in a progressive step-wise sequence [23]. The uniformitarian nature of typo-chronologies leads to the creation of non-overlapping chronological units, where generally, short time spans are thought to reflect dynamic societies in more troubled times, whereas longer time spans reflect peaceful periods [32]. Transitions between non-overlapping chronological units are inadvertently treated as abrupt, disregarding the temporality of transformation processes within and between periods. More detailed chronologies, with shorter periods, can be created when material culture changed relatively quickly, whereas slower change leads to the creation of longer periods [32]. Typo-chronologies are good at showing long-term and geographically broad patterns of change in the archaeological record and they can help us understand how change in one domain of a society, such as change in the material culture or the introduction of a new burial practice, might be linked to broader transformations in the society and its environment. Typo-chronologies are dependent on arbitrary geographical constrains such as modern countries or regions, which will obscure spatial differences within the selected area and between different areas.

Burials have traditionally provided closed-context material for typo-chronological analyses [e.g. 1], but because mortuary practices are often conservative in nature [33, 34], the temporal scale of grave-good chronologies might not equal the temporal scale otherwise observable at contemporaneous settlement sites [35]. Another challenge to chronological analyses of burial assemblages is the possibility of objects being passed between generations as heirlooms [30]. It has been suggested that pottery from graves is not representative of the style otherwise in use at the time of death, but rather a style produced or selected specifically for funerary purposes [36]. There are demonstrated difficulties in correlating metalwork and pottery typologies from the PRIA in Denmark [7]. This may be due to different temporal sensitivity, where metalwork patterns are intrinsically more fluid than pottery shapes, leading to expected higher rates of change in metalwork types. Pottery typology was also regarded as more conservative in Early Iron Age urnfield material from Schleswig-Holstein [9]. There is a need to evaluate existing typo-chronologies and resolve possible temporal discrepancies using absolute dating, but this is not a straightforward process, as evidenced by ^14^C dating of sequential cultural layers with diagnostic pottery, whose results can be difficult to reconcile with existing chronological frameworks [e.g. 37]. Such studies commonly use pottery types as markers of certain periods or phases, but without investigating the currencies of the individual types it is not possible to conclude whether it is the chronology or the typologies, or indeed both, that are in need of a revision.

### Modelling change within a Bayesian framework

Bayesian statistics offers a coherent statistical framework for evaluating and interpreting statistically independent likelihoods for the calendar dates of events associated with an archaeological phenomenon in view of our relative dating information [38]. Calendar information will often be calibrated ^14^C ages, but thermoluminescence dates, dendrochronological dates, numismatic or historical dates can equally be included. A Bayesian chronological model combines probability distributions of calibrated dates with prior information consisting of expert observations obtained independently of the likelihoods. This ‘prior information’ can either take the form of informative priors based on the temporal relationships between samples, such as relative dating based on traditional stratigraphy or typology, or as uninformative priors that impose a statistical distribution on the dated events, e.g. assuming that the calendar dates of potential ^14^C samples are uniformly distributed between start and end boundaries [39, 40]. The model uses this prior information to constrain the statistically most likely date ranges (*posterior density estimates*) of the ^14^C samples. Bayesian chronological models can estimate dates of events that cannot be dated directly, such as when burial activity started at a cemetery, or when an artefact type came into use, which allows us to investigate change as a process and to test existing interpretations of causal processes. The introduction of an artefact type at more sites can be modelled individually but modelling its spatio-temporal progress across the landscape using geography as prior information, remains challenging in spite of recent progress [e.g. 41].

In most cases, a Bayesian chronological model will significantly improve the overall precision of directly dated events compared to individual calibrated ^14^C dates, but studies coinciding with plateaus in the ^14^C calibration curve remain challenging. Here even relatively precise ^14^C ages for short-lived samples can produce calibrated date ranges spanning the whole plateau, or at best, give multimodal distributions offering alternative solutions in different centuries. This study is engaging with the ^14^C plateau c.750-400 BC [42, 43], also known as the Hallstatt plateau [44, 45], which has been detrimental to studies of Early Iron Age chronologies in Europe [46], but also with a later inversion of the calibration curve which inevitably yields multimodal distributions for samples formed in c.320-200 cal BC. With recent developments in ^14^C science, however, such plateaus and inversions might no longer be a ‘catastrophe’ for archaeological chronology [16]. Developments include ^14^C measurements with increasing precisions [e.g. 47] and new algorithms applying less smoothing to the new IntCal20 calibration dataset [48]. Finally, IntCal20 include calibration data at single-year resolution for the first half of the Hallstatt plateau [49–51], although not for the second half, which coincides with the Danish urnfields. Recent studies using Bayesian chronological modelling have demonstrated that is possible to model case studies on ^14^C calibration plateaus, although applications tend to target materials whose dates are constrained by strong prior information based e.g. on stratigraphy [52, 53], floating tree-ring series [54, 55], or archaeogenetic evidence [56]. This study however employs relatively weak constraints, assuming that artefacts sharing certain characteristics (i.e. of the same type) are more likely to date close to each other than far apart, and if a representative number of samples are dated from each type, their currency can be modelled, i.e. the period a certain artefact type was in production and in primary use. There is no available prior information from traditional stratigraphy, e.g. constructional or depositional sequences, although urnfield site formation has been described using horizontal stratigraphy [57, 58].

A majority of published Bayesian chronological models assume that change occurred too rapidly to detect within the resolution of the ^14^C dating method. Although this is a useful working assumption and probably not grossly misleading in most cases [59], cultural change is an ongoing process and whether that is visible in the archaeological record depends on the temporal resolution of the available data. This is certainly true for the material record and Brainerd stated that”*Each type originates at a given time in a given place*, *is made gradually in increasing numbers as time goes on*, *then decreases in popularity until it becomes forgotten*, *never to reoccur in an identical form*” [27]. Brainerd’s statement has been demonstrated using known-age datasets [60, 61], but is not supported by the commonly used uniform distribution bounded-phase model, which assumes that archaeological activity began and ended abruptly [62]. The Bayesian community has been long been aware of this [63], but there was no better alternative available before Lee and Bronk Ramsey [64] developed a trapezoidal distribution model, which was introduced in OxCal v.4.2 [65]. They based it on earlier works by Karlsberg [63], who found that assuming different *a priori* information about the rate of deposition has a significant influence on the posterior density estimates and the archaeological interpretations thereof. The trapezoidal model splits the distribution into three parts: a gradual increase (introductory period), a period with a constant rate of activity (blooming period), and finally a gradual demise (period of decline). This distribution corresponds well with the currency model suggested by Trachsel based on his re-evaluation of Hallstatt chronology [30]. The trapezium model provides an alternative approach for modelling transitional processes, allowing the transition to have a duration without having prior knowledge of which dated cases belong in the introductory period, blooming period or period of decline [64, 66]. It remains a more demanding model in terms of computational time and number of dated cases required to reach useful posterior estimates, but the current study aims to apply it whenever it is suitable and possible.

A single ^14^C age can be associated with more items from the same context, e.g. the death of an individual, the decline of artefact type A, the blooming of artefact B and the introduction of artefact type C. Cross-referencing events between different elements in the model is a powerful tool that allows a single likelihood to be evaluated over a range of prior information [e.g. 67]. Extensive use of cross-referencing does however remain computationally challenging and it can be necessary to duplicate posterior estimates using the OxCal function Prior, which allows the same likelihood to be sampled multiple times [65].

### Early Pre-Roman Iron Age in Southern Scandinavia

In Southern Scandinavia, and in particular southern Jutland, the PRIA appears to be a hybrid cultural group sharing traits of material culture, house types, economy and funerary practices with the Jastorf core area in Schleswig-Holstein and Mecklenburg-Vorpommern [68], and the Lower Rhine area [69]. Around the start of the PRIA, iron began to be extracted locally in Denmark, substituting the previous dependence of imported bronze and limiting the import of artefacts with a wider European distribution [11]. Jutland is divided into three regional groups, based on differences in material culture and funerary practices: southern Jutland, middle Jutland and northern Jutland [1]. The Early PRIA (c.500-250 BC) has been described as a seemingly peaceful period [e.g. 70], although it coincides with a peak in deposition of weapons and human bodies in bogs [71, 72], the first fortified settlements [73], and the famous deposition of the Hjortspring boat or war canoe [74]. This instead indicate the presence of inter-personal or even religiously or societally initiated violence, raising doubt as to how peaceful the period really was. It is difficult to detect any social stratification in this period [75], but rather than being a truly egalitarian society, social hierarchy was likely expressed in ways that are not archaeologically recognisable. This argument is supported by an ongoing reorganisation of the cultural landscape in the Early PRIA, as demonstrated by the fortified settlements, the introduction of Celtic fields demarcating land ownership [76], and the pit zone alignments (Danish: hulbælter) with possible implications of fortification, control of movement of people and cattle [70].

#### The urnfield funerary tradition

In Southern Scandinavia, funerary practices changed from inhumation to cremation around the transition from Early to Late Bronze Age (c.1100 BC), although cremations appear earlier in some areas. In Thisted in north-western Jutland c.10% of all burials dated to Bronze Age per. II (1500–1300 BC) are cremations, and in Schleswig-Holstein cremation was introduced around 1300 BC [77]. Burials continued into the Early Iron Age to take place at the large Early Bronze Age burial mounds. In parallel with this, the urnfield funerary tradition was introduced in Southern Jutland at the beginning of the PRIA and the large collective burial grounds mark a fundamental break with earlier funerary practices [11]. The Danish urnfield tradition is a late part of a wider European tradition [2, 69] and comparable large urnfields dating to the Late Bronze Age-Early Iron Age are found in the neighbouring regions of Northern Germany, e.g. the urnfields Schwissel [58], Groβ Timmendorf [77] and Mang de Bargen [78] in Schleswig-Holstein, and Mühlen Eichsen [79] in Mecklenburg-West Pomerania. This paper however focusses on the Danish urnfield tradition.

The Danish urnfields were first described at the end of the 19^th^ century, when the small burial mounds were still visible in the landscape [4, 5]. By 1984, Madsen and Neergaard had excavated the central part of close to 500 urnfield graves from 10 sites in Southern Jutland and although they largely ignored the rest of the burial mounds, they concluded that the character of the urnfield were so homogenous that future excavations were unlikely to alter this picture [4]. More urnfields were however excavated during the first half of the 20^th^ century to obtain datable material for typo-chronological studies [1, 80, 81]. New urnfields continue to be recorded through development-led excavations, aerial photography and LiDAR remote sensing [e.g. 82] and to date, 66 certain and another 22 possible urnfields have been identified [15]. The urnfields are primarily situated in south-west Jutland, where the landscape is characterized by outwash plains with primarily nutrient-poor sandy soil. The topography is low-lying and smaller rivers, lakes and bogs are scattered across the landscape, which to the west is bordered by the Wadden Sea [83]. The urnfields are often constructed in the vicinity of Neolithic or Bronze Age burial mounds, continuing the Bronze Age tradition of demarcating major routes of transport by lines of burial mounds, but at the same time separate from contemporaneous settlements [77, 84].

**Fig 3:**
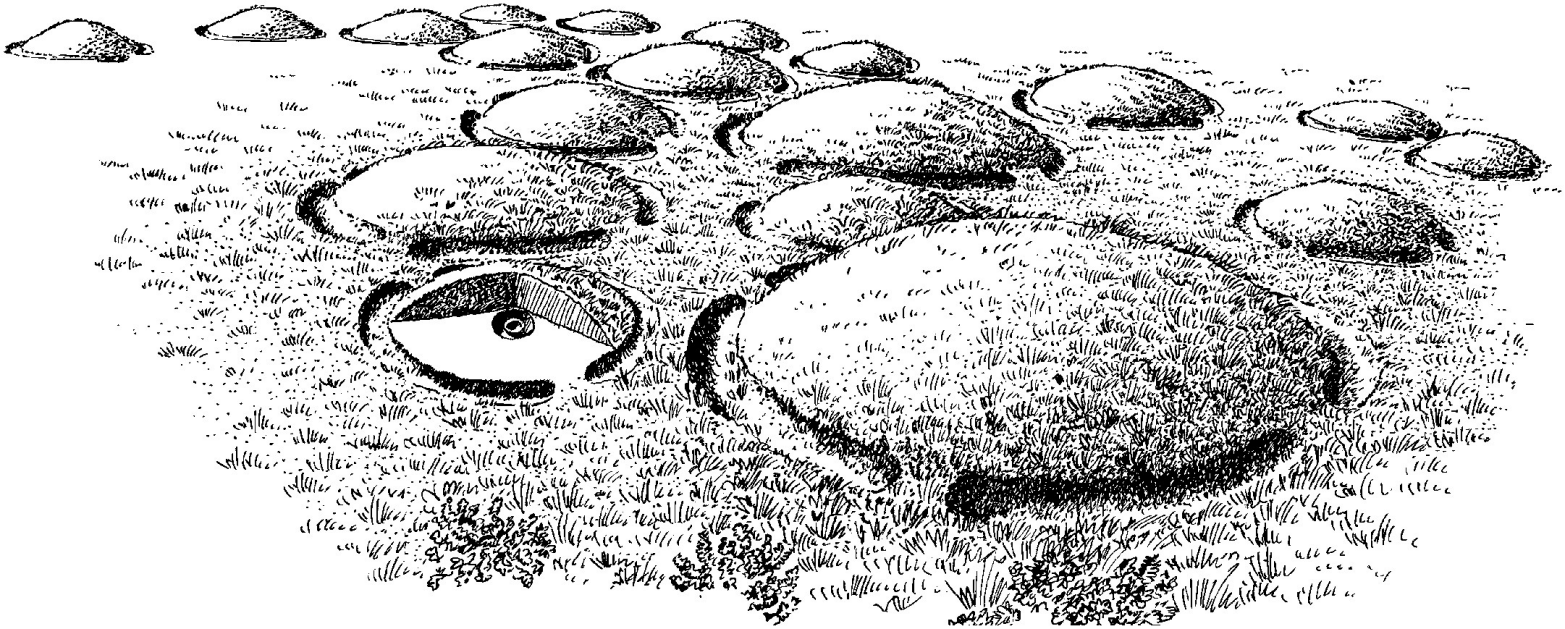
Reconstruction of urnfield in Kelstrup plantation forest, Southern Jutland. The small mounds are encircled by ditches with interruptions, cross-section of one mound to show the central urn grave (drawing by J Andersen, published under a CC BY license 4.0 with permission from original copyright holder Museum Sønderjylland.

Following the Danish urnfield tradition, the deceased were cremated, and fragments of calcinated bone were deposited in ceramic vessels along with metal objects and buried individually under a small burial mound or hillock (Danish: tuegrav; Fig 3). Approximately 1/3 of burials contained metal objects regarded as possible dress accessories. At some sites, a small part of the pyre debris (i.e. charcoal and other charred archaeobotanical remains) was also transferred to the urn. There is no evidence of cremation pyres near the urnfields and in fact, few prehistoric pyre sites are known from Denmark. Pyres can be preserved if they are covered shortly after the cremation event, e.g. by a burial mound, but even then, it can be difficult to identify them archaeologically, as evidenced by experimental cremation studies [85]. A smaller number of other grave types also occur, such as ‘bone-layer graves’ without a (discernible) container, ‘urn-bone layer graves’ as a hybrid burial form containing cremated bone and pottery sherds (grave types after [86]), and at some sites graves are covered by a stone paving rather than a hillock [87]. A key feature of the urnfield tradition is that each grave is encircled by a ditch with a varying number of interruptions, forming bridges into the central hillock [1]. The hillocks were created by using the soil from the circular ditch, and have estimated original heights of 1–2 m and diameters from a few meters up to 11 m. At some urnfields, the hillocks were further demarcated by kerbstones or wooden posts [88].

The urnfields vary from a few and up to around 1500 closely spaced graves. The graves are clearly demarcated by open circular ditches and generally do not inter-cut [88], making it difficult to determine any direct stratigraphic relationship between graves. Horizontal stratigraphy has however been observed at several urnfields [1, 7, 58, 88, 89].

#### Material record

Urnfield artefact assemblages only represents part of the PRIA material record, but the relative chronological framework of the period has historically been based on typo-chronological studies of these assemblages. There are no weapon graves from the period and all artefacts of metal are related to the personal dress attire, representing a limited range of dress accessories and other related adornments. Dress pins from the LBA are made in bronze, but with the onset of PRIA, the pins are primarily made of iron. Neck rings and certain pin types do however continue to be made of bronze. The repertoire of pins and belt equipment is similar to but more restricted than material from Schleswig-Holstein [9, 90–94]. A list of type names in English and Danish is provided in S1.1.1 Table in S1 File. According to the typo-chronology of Jensen [10], the earliest pin type has a coiled head and a bend in the upper part of the pin, just beneath the head. In period I.1b-I.2a, the bend moves down the needle, creating a neck beneath the head. Pins with circular head and bomb-shaped head are introduced in period I.2. In period II.1, Holstein pins, pins with grooved head, winged head pins and pins with rod-shaped head appear. Different types of iron belt equipment appear from period I.1 onwards, starting with iron rings with eyelets. In period I.1b, belt hooks with protruding clasps are introduced, initially with a tongue-shaped outline, followed by a triangular outline, before becoming narrow in outline by period II.1. Also occurring in period II.1 are iron rings with shank [10].

**Fig 4:**
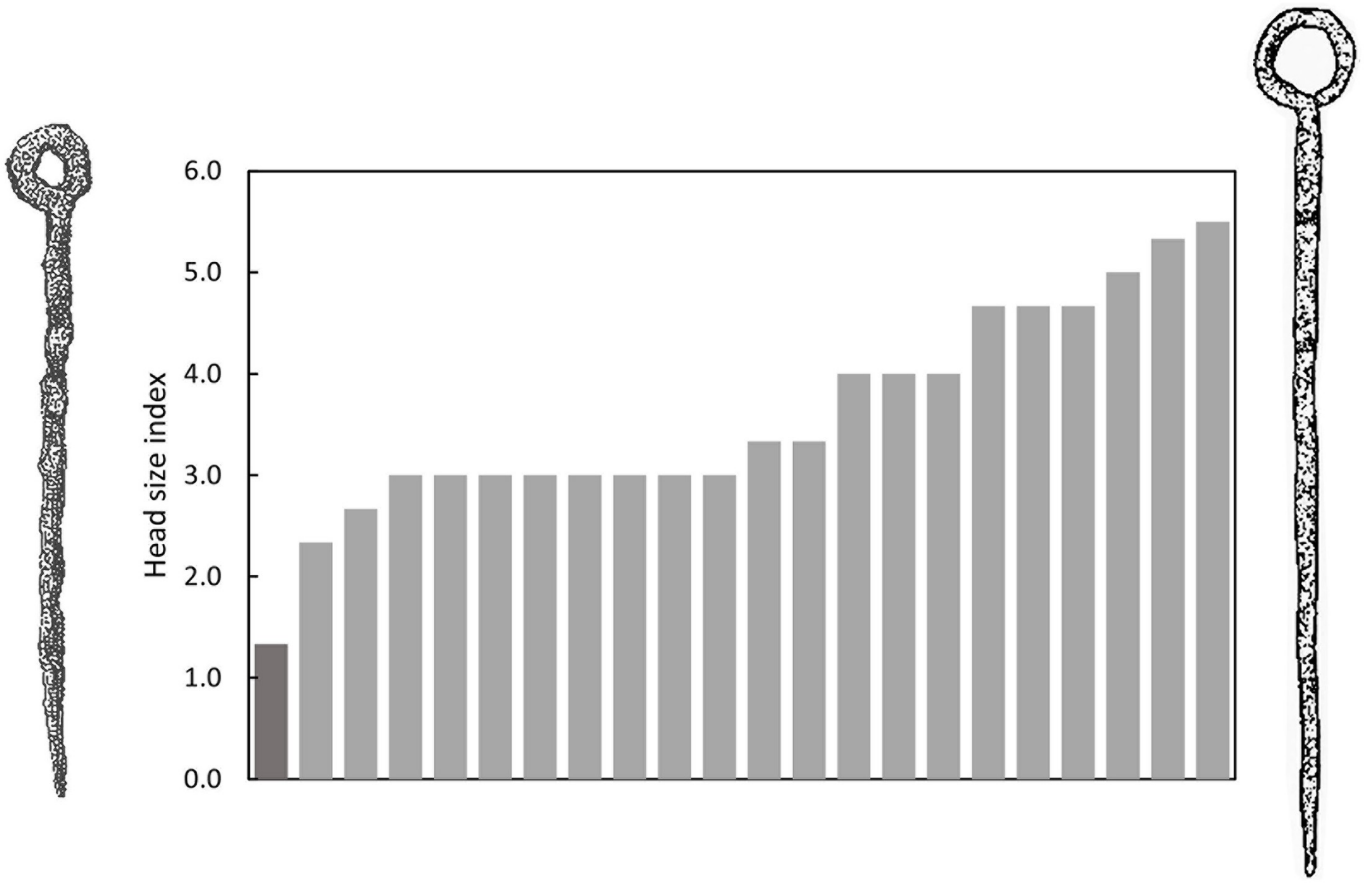
Distribution of head size index (inner head diameter divided by pin width) of 22 pins with circular head from 11 graves from Aarupgaard urnfield. Each column presents a single pin and only one pin from grave U123 qualifies as having a small head (darker grey column and depicted to the left of the plot). To the right of the plot is depicted the pin with the largest head size, from grave U500.

One exception from Jensen’s typology [10] is made regarding the subdivision of pins with circular head based on the size of the head. Pins with ‘large’ heads (diameter >2x pin width) are supposedly introduced a little earlier than pins with ‘small’ head (diameter <2x pin width). We however only found one pin, from Aarupgaard grave U123, with a small circular head following this definition. When the head size index (diameter of inner head/pin width) of all the pins with circular heads from Aarupgaard is plotted, they demonstrate a continuous distribution without any clear typological categories (Fig 4). Unfortunately it is not possible to explore whether the differences in the head sizes that we do observe might be linked to age, sex or gender of the deceased. This analysis only considers material from Aarupgaard, and warrants further investigation, but for the present study, we suggest that pins with circular head belong to a single typological group, and we will treat them as such in the following.

**Fig 5:**
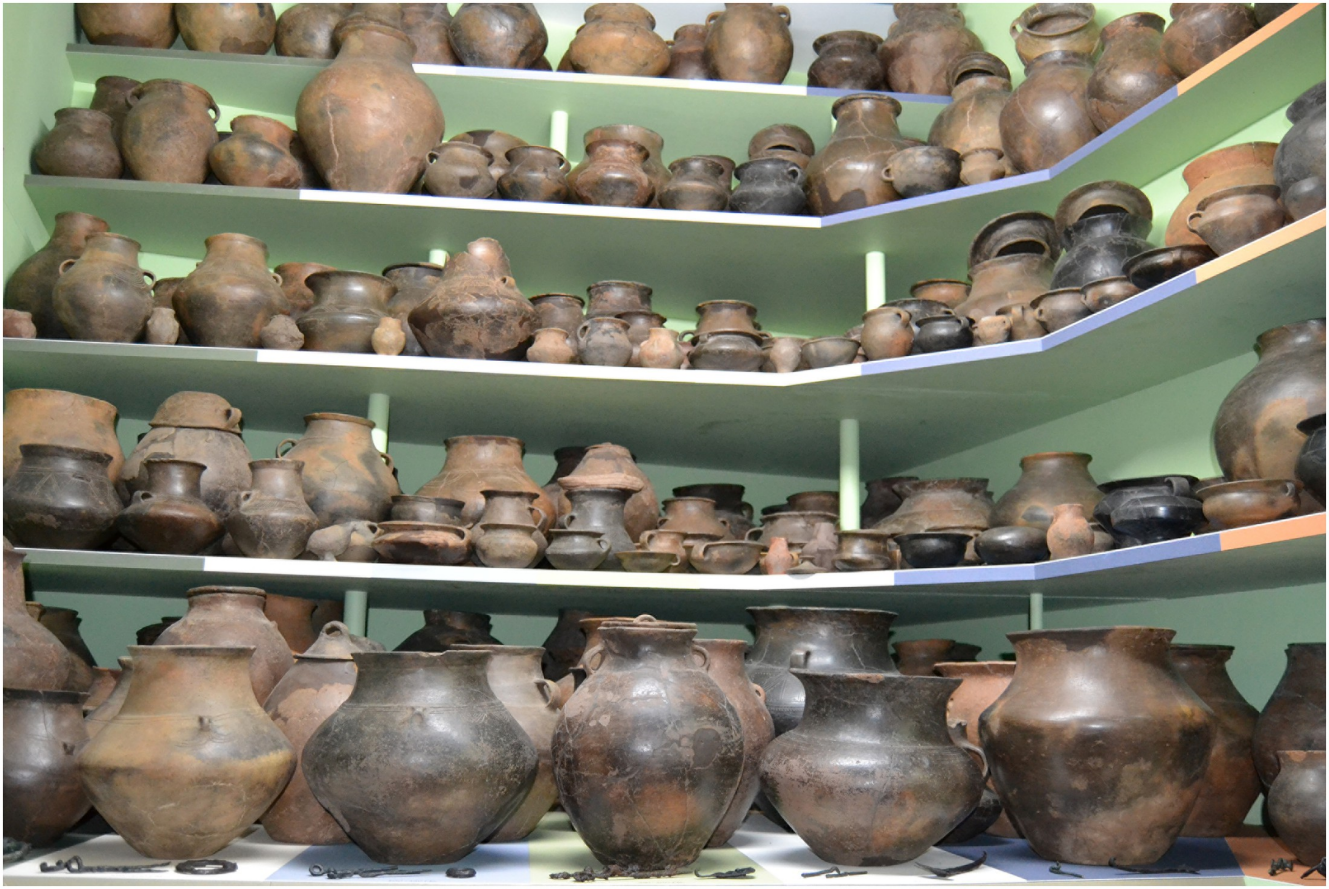
Pottery from Aarupgaard urnfield on exhibition at Haderslev Museum. Medium-large sized vessels are funerary urns, with and without handles and lids, small bowls and cups are accompanying vessels from either the burial pit or the circular ditches (photo: H.A. Rose).

The full pottery repertoire found at settlement sites includes a range of vessels in different sizes, but at burial sites, most pots are large-medium sized storage vessels used as funerary urns, and small bowls and cups used as accompanying vessels (Fig 5). Ornamentation is rare and although vessel rim shapes are chronologically sensitive [7] they have often been destroyed by agricultural activity, making them ill-suited for chronological studies. Instead, typological division is based on vessel proportions (height and width) [10]. Individual types are designated by a number referring to the shape of the vessel body, and a capital letter referring to the shape of the vessel neck (S1.1.1-S1.1.3 Tables in S1 File.). Some of the types remained in use for most of the Early PRIA and then disappeared at the transition to Late PRIA, when several new types were introduced. Vessel shapes 11, 12 and 13, with cylindrical or concave necks, were most prevalent in the Early PRIA, with early types 11A, 11D, 12A, 13A and 13C, and late types 13B, 13D, 15B, 15C and 18C. Vessel shapes 16, 17 and 20, with no necks, were most prevalent in Late PRIA [10].

#### Aarre urnfield

**Fig 6:**
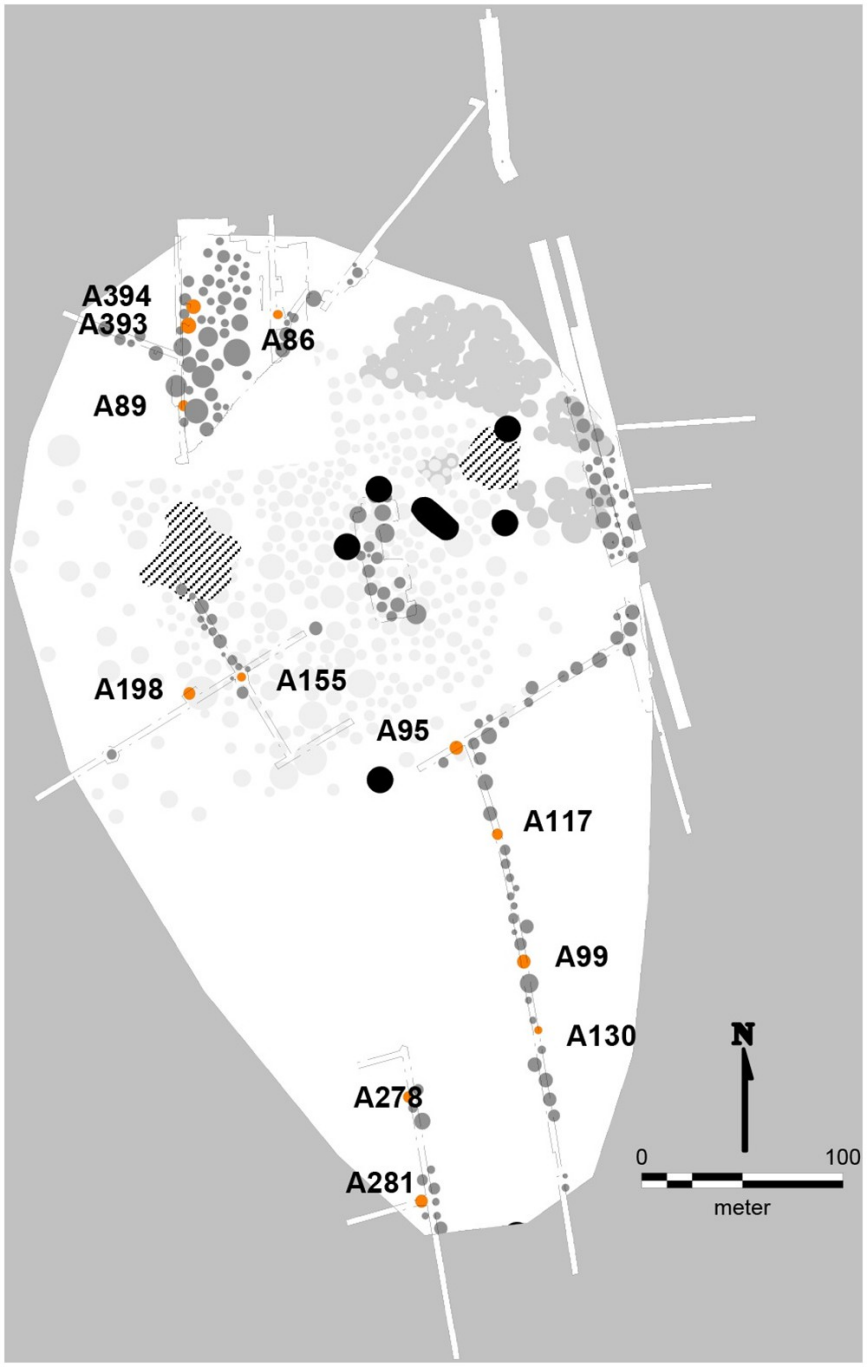
Layout of Aarre urnfield. ^14^C dated burials are marked in orange and with corresponding A numbers. Map created by L. C. Bentsen under the CC BY license 4.0.

Aarre urnfield is situated on Esbjerg Bakkeø, a low hill island surrounded by wetlands. The site was first investigated by antiquarians in the 1890s, and it came to play an important role in the definition of a chronological framework for the PRIA in Southern Scandinavia [1, 4, 10]. Following recent rescue excavations, it is estimated that approximately half of the original burial ground of c.10,000 m^2^ with up to 1000 burials has been archaeologically investigated, making it the second largest urnfield in Denmark (heritage registration: VAM 1600, ARV 113, ARV 115; Fig 6). Three main lines of large burial mounds converge at Aarre, and at least 10 Neolithic and Bronze Age burial mounds are located within the urnfield [95]. The urnfield burial organization has been described using horizontal stratigraphy, with the earliest urn burials located around a small group of these older burial mounds, and from this point, the urnfield expanded outwards in several directions [1]. Approximately 1/3 of the urn burials contained metal objects primarily in the form of dress pins of either iron or bronze, different types of belt hooks, chains and O-rings. The site is estimated to have been in use c.500-250 BC, based on metalwork typo-chronology [1, 95].

#### Søhale urnfield

**Fig 7:**
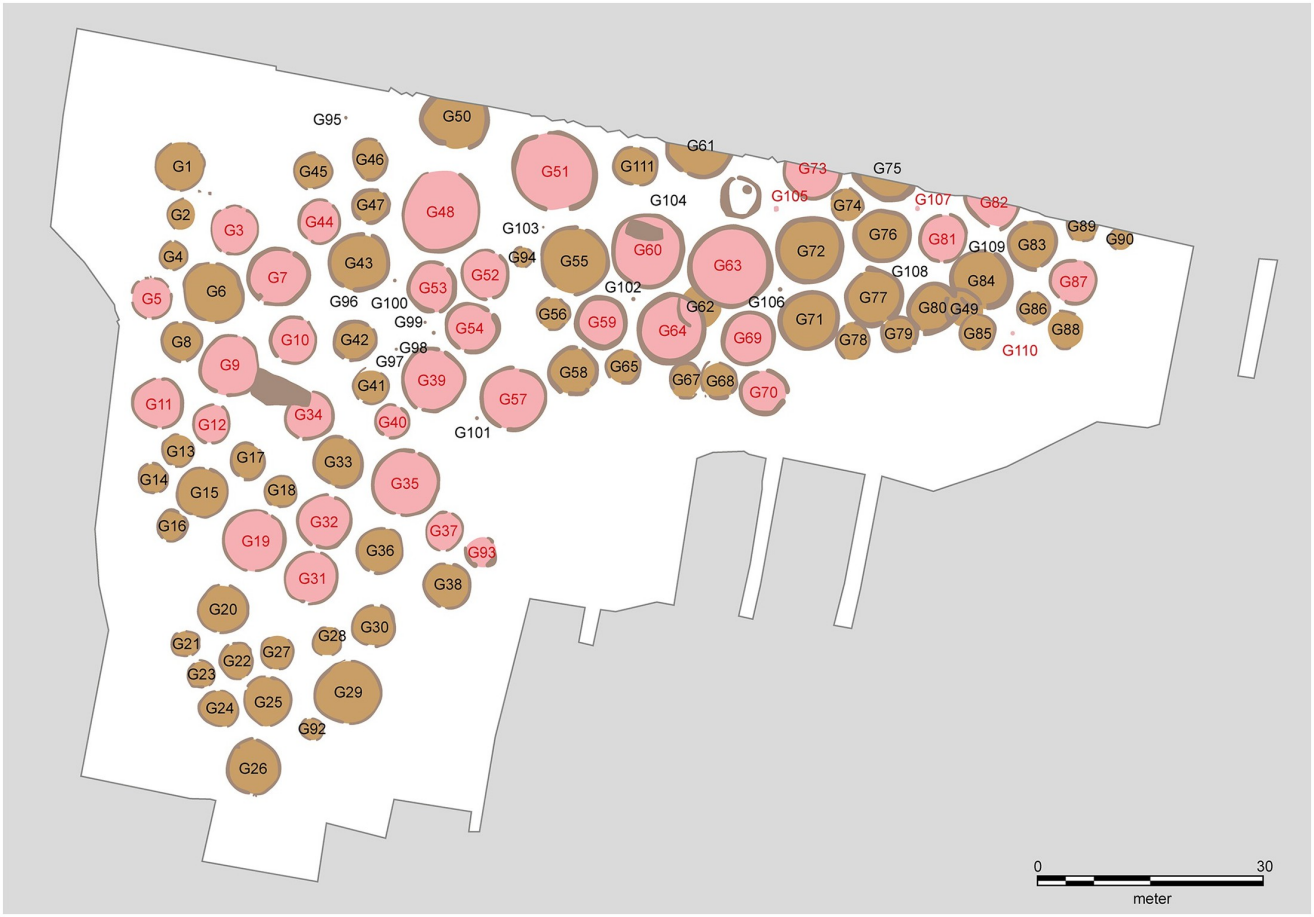
Layout of Søhale urnfield. Grave numbers of ^14^C dated burials in red. Map adapted by R. Potter from [15] under a CC BY license, with permission from N. A. Møller, original copyright 2020.

Søhale urnfield is situated 10 km inland on a plateau, delimited by streams and wetlands to the east, north and west. 700 m east of the site is a line of burial mounds demarcating a major transport route. Some 30 m northeast of the site are two large, older burial mounds that might have served as a point of origin, but a modern road prevents further investigation. The site was excavated in 1996 as part of a development-led excavation in advance of gravel extraction, and the extent of the burial ground to the west, south and east was documented, whereas a modern road cuts it to the north (heritage registration: ESM 2139; Fig 7). 93 urnfield mounds were recovered and 73 of these contained a centrally placed urn burial enclosed by a circular or slightly oval ditch with a diameter of 2–10 m. Besides the standard urn burial, the local burial tradition also includes bone-layer burials, urn-bone layer burials, and possible cenotaphs with and without mound and circular ditch [15]. According to Møller et al. [15], the cemetery started out as two separate burial groups differentiated by burial types, and although the burial groups merged over time, the differences remain, possibly reflecting different communities sharing the burial ground for three centuries (c.500-200 BC).

Approximately 1/3 of the urn burials contained a limited range of metal objects, primarily in the form of dress pins and different types of belt equipment. Following excavation, the recovered cremation urns were left in museum storage, with their contents still intact, but they have recently been computed tomography (CT)-scanned and analysed as part of a renewed investigation of the Danish urnfield tradition [15]. Based on diagnostic artefacts and 22 ^14^C dates on cremated bone from 21 burials, they find that Søhale was established in the later 6^th^ century BC and continued in use for about three centuries (c.530-250 BC).

#### Aarupgaard urnfield

**Fig 8:**
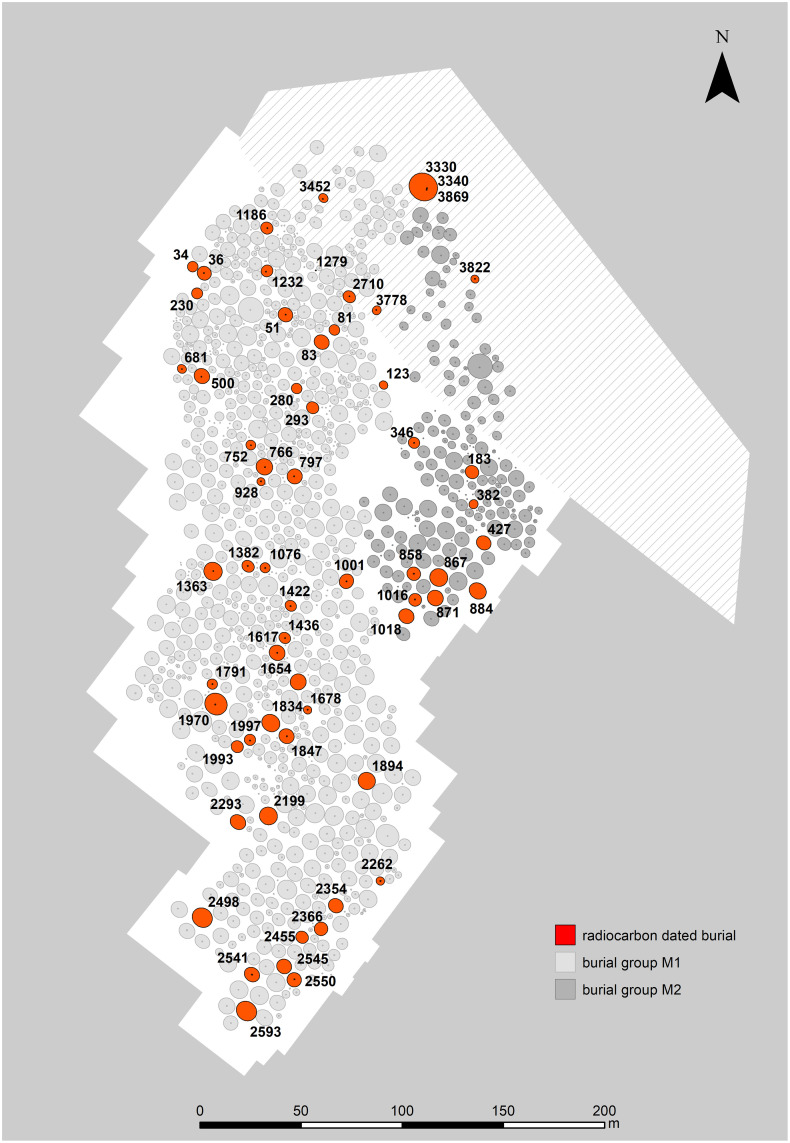
Layout of Aarupgaard urnfield. Grave numbers of ^14^C dated burials in red and the topmost large red circle is a Bronze Age burial mound containing three founding graves. Burial group M1 in light grey, burial group M2 in dark grey, the shaded area is protected under Danish heritage legislation. Map created by K. Göbel under the CC BY license 4.0.

Aarupgaard urnfield is situated on the western point of a low hill, bordered by two streams, Gram Å to the north and Gels Å to the south. The site was first registered in the late 19^th^ century and later totally excavated in 1970–72 (heritage registration: HAM 1070) [81, 88]). A Bronze Age burial mound serves as point of origin for up to 1500 urn burials, and although this mound was largely destroyed before excavation, within it were found three urns typologically dating to the transition period LBA-PRIA (burials U3330, U3342 and U3869). They are probably among the first burials at the site, and from their location the cemetery expanded southwards, which can best be described using horizontal stratigraphy. Aarupgaard is by far the largest urnfield in Denmark and covers an area of c.60.000 m^2^, measuring c.100-200 m across (E-W) and c.450 from north to south. It can be divided into a large western group (orange circles, Fig 8) and a smaller eastern group (light yellow circles, Fig 8), both spreading out from the Bronze Age burial mound (red circle, Fig 8) and in use throughout the chronological sequence of the site, albeit with differing burial tempi. Approximately 30% of the burials contained metalwork and based on diagnostic artefacts, horizontal stratigraphy and other architectural features of the burial monuments, it has been suggested that Aarupgaard urnfield was in use c.500-100 BC and that it can be divided into a number of phases [7, 75, 88].

#### Osteological profile of urnfield burials

Osteological analyses of cremated human remains have been carried out for 67 burials from Søhale urnfield [15] and 58 burials from Aarupgaard urnfield (report by Harvig from 2019). Another 130 burials from Aarupgaard were analysed in the 1970s by J. Balslev Jørgensen, but the unpublished report does not mention what methods were used. Burials from Aarupgaard were selected to include varying amounts of bone, degree of preservation and from the entire chronological sequence. Results show that bone is fully cremated with a white to greyish-white colour, reflecting consistent pyre temperatures around 800 °C. The homogenous material reveals that a highly standardized modus operandi of both cremation and post-cremation handling was in place at both sites. Each burial is anatomically representative of a single individual and individuals of both sexes and from all age groups were identified. The majority lived to full adulthood, but 28% of the burials from Søhale contained sub-adults, as did 20 out of 58 burials from Aarupgaard. This is remarkable, because sub-adult individuals are often poorly represented or even missing from archaeological contexts [96], although significant numbers of sub-adults in relation to adults from cemeteries dating to the PRIA have been noted elsewhere [e.g. 97]. Even though only part of the c.900 burials from Aarupgaard with preserved cremated human remains have been osteologically analysed (c.6% using modern osteological methods), whereas all available material was analysed from Søhale, the funerary practices appear to be comparable between the two sites. It is not possible to compare the demographic profiles, beyond noting the large proportion of sub-adults at both sites.

### Research objectives

Bayesian chronological modelling is used extensively in archaeology today to construct fine-grained regional chronologies [e.g. 98], and it enables the correlation of chronological frameworks across larger regions with no shared material culture. This has made it abundantly clear that the past is more complex than previously anticipated, e.g. the origin and spread of megaliths in Northern Europe [99, 100], but also that the rate of change has increased and decreased over time, questioning the uniformitarian nature of chronological frameworks [e.g. 101]. This study presents a dynamic chronological examination of PRIA artefact assemblages from Aarre, Søhale and Aarupgaard urnfields, all located within the core urnfield area in south-west Jutland, Denmark. It continues a long history of chronological research into the urnfields, but brings it up to date by applying a research approach that combines archaeological expert knowledge with ^14^C data in a Bayesian framework. We model the temporal distributions of a range of artefact types, i.e. their currencies, spanning more archaeological phases in order to identify possible periods with varying rates of change in the material culture. Building on this, we provide an absolute chronological framework for the PRIA, enabling correlation with chronologies from other regions.

This study is concerned with temporal changes in a regional material culture, but rather than viewing these as isolated incidents, they can be linked to changes in other spheres of society, indicating major turning points in prehistory. Without a detailed understanding of the chronological framework and the material culture on which it hinges, it is difficult to address overarching questions regarding for example social structure, economy and religion.

## Material and methods

### Sample selection

Ideally, we only wish to include urnfield graves containing mixed find assemblages of diagnostic pottery and metalwork, along with cremated human remains that can be ^14^C dated and thus provide an indirect date for the artefacts. Up until the 1980s, urnfield excavations generally produced well-preserved material, but cremated human remains were not routinely archived before the 1970s. Agricultural activity over the last four decades has had a severe impact on these shallow structures, and although new urnfields are continuously recorded, they are increasingly poorly preserved [15]. This leaves a narrow timeframe for urnfield excavations that could provide enough material for this study, which is even further limited by only about 1/3 of the burials containing metalwork. We identified three sites that met the quality criteria and gained permits for destructive sampling and ^14^C dating from ARKVEST—Arkæologi Vestjylland (Aarre urnfield), Sydvestjyske Museer (Søhale urnfield) and Museum Sønderjylland (Aarupgaard urnfield).

**Fig 9:**
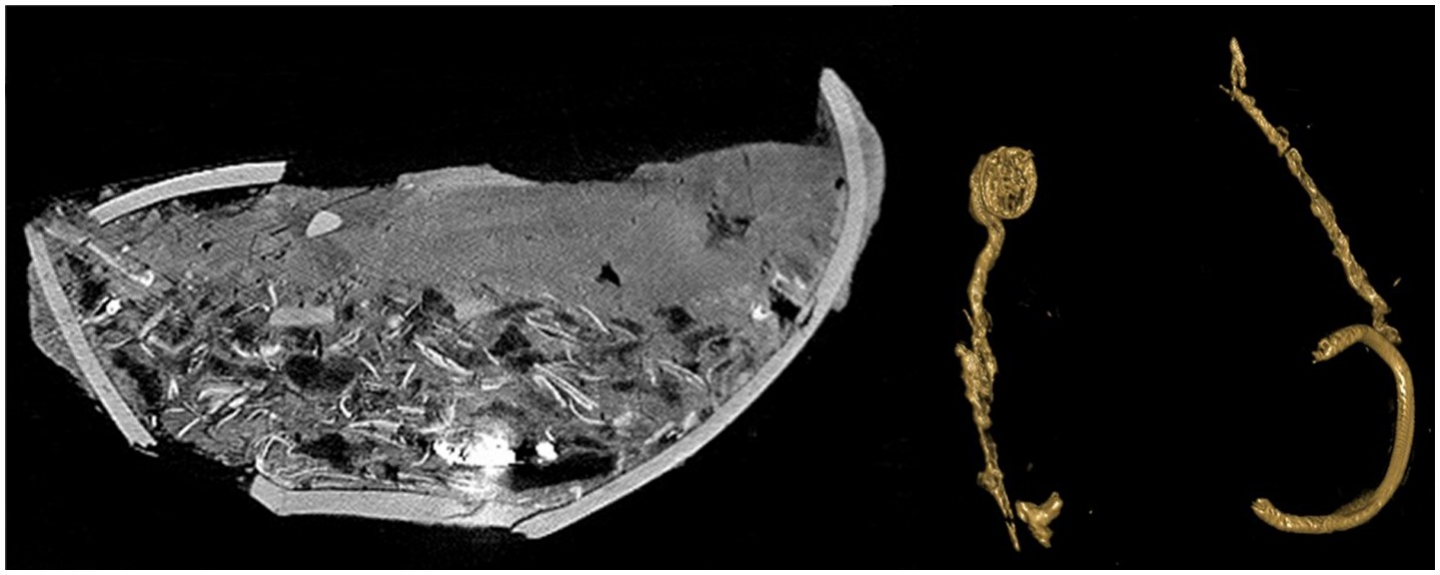
CT images of urn grave A278 from Aarre urnfield. To the left is a cross section of the fragmented urn that contain a bottom layer of cremated bone and some fragments of metal. To the right the metal has been isolated and it is possible to determine a bronze ring and two pins with disc-shaped head. Urn and artefacts are not to scale Modified by H. A. Rose and published under a CC BY license 4.0 with permission from original copyright holder Arkæologi Vestjylland).

Fragments of cremated bone were sampled from Aarre, Søhale and Aarupgaard urnfields, preferably cortical bone from diaphyses of major long bones (humerus, femur and tibia), but in cases of heavy fragmentation other skeletal elements were selected. Aarupgaard was excavated in the early 1970s and was the only site that provided well-preserved, mixed find assemblages. Aarre and Søhale urnfields were excavated more recently, and here pottery was preserved well enough to be included in only a few cases. Metalwork was also rather poorly preserved, but urns were micro-excavated in a controlled indoor environment after CT scanning. CT is in principle digital x-ray performed in 3D and it has in recent years been applied to prehistoric cremation urns more regularly [e.g. 102]. In this case, the CT images enabled the identification of even very fragmented artefacts (Fig 9).

### Laboratory analyses

Samples of white cremated bone (CB) were selected for dating. To confirm they were fully calcined, aliquots of powdered untreated CB were analysed by Fourier-transform infrared spectroscopy (FTIR). The crystallinity index (CI) was estimated as the splitting factor between the two absorption bands at ca. 603 and ca. 565 cm^–1^ (CI = (A_603_+A_565_)/A_valley_) [103, 104]. The samples were dated by the Leibniz-Laboratory (KIA-), Kiel, Germany, the Center for Isotope Research (GrM-), Groningen University, the Netherlands, and the Royal Institute for Cultural Heritage (RICH-), Brussels, Belgium. Pretreatment of samples of cremated bone varied between the laboratories, with Groningen using the traditional acetic acid treatment and Brussels and Kiel using variations of an acid-leaching treatment. A comparison study has however demonstrated these differences to have no influence on the results [105].

#### Pretreatment and combustion

Brussels leached ca. 30% by weight of each solid sample of cremated bone in 1% HCl, before it was powdered and treated with 1% acetic acid (24 hr) to remove calcite [106, 107]. Kiel crushed each sample before treating it with 0.6% (1M) acetic acid (5 ×30min) and leaching ca. 50% with 1% HCl [19]. Groningen treated the samples with 1.5% sodium hypochlorite (NaOCl, 48 hr, 20°C), followed by 6% (1M) acetic acid (24 hr, 20°C) [17, 108]. All extracts were reacted with phosphoric acid to produce CO_2_.

#### Graphitization and AMS measurement

Purified CO_2_ was reduced to graphite for AMS measurement. Kiel measured double targets on samples from 17 later burials from Aarupgaard urnfield to reduce measurements uncertainty; this does not equal full replication including new extractions, but we know that a large part of the final uncertainty comes from the combustion, graphitisation and uncertainty in the standards measured concurrently, and these errors are independent between the paired targets. Measurements in Brussels were performed on a Micadas (195.5 kV) AMS system [109], Kiel used a HVEE 3MV Tandetron 4130 AMS system [110], and Groningen used a Micadas (180 kV) AMS system [108]. All resulting ^14^C-contents was corrected for fractionation using the simultaneously AMS measured ^13^C/^12^C isotope ratios [111].

## Results

### Artefact frequencies

**Fig 10:**
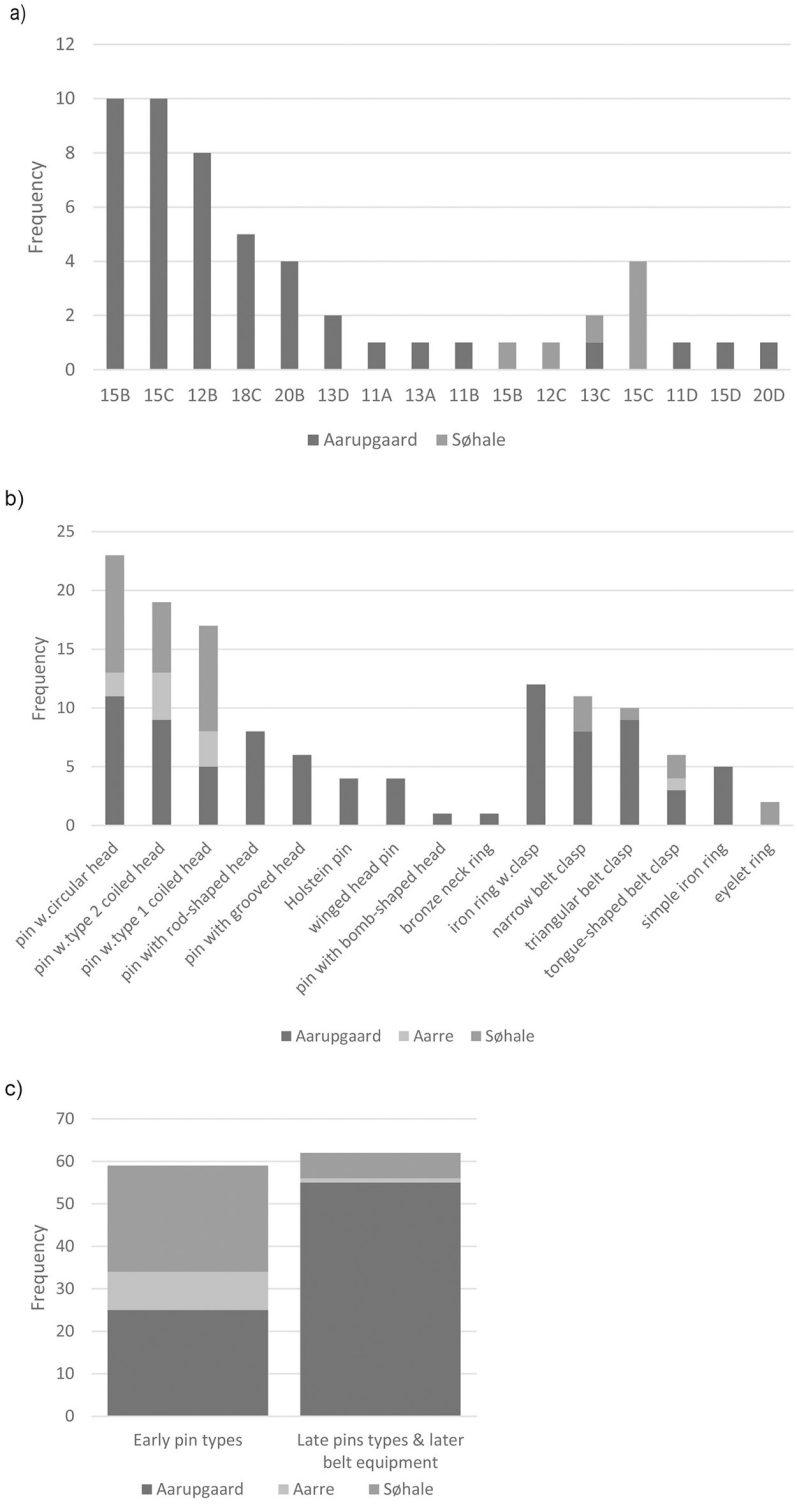
Frequency histogram of artefact types from Aarupgaard, Aarre and Søhale urnfields. Depicting types of a) pottery, b) metalwork, c) number of burials containing early pin types (pins with type 1 and 2 coiled head, pins with circular head), and later occurring pin types (Holstein pin, pin with bomb-shaped head, pin with rod-shaped head, pin with grooved head, winged-head pin) and belt equipment (iron ring with shanks, all types of belt clasps). Typology after Jensen [10].

Typological analysis of pottery and metalwork rely on published data on pottery by Jensen [10], registrations by Terkildsen (master thesis from 2005, Aarhus University), and supplemented by analyses carried out by the first author. Some of the burials from Aarupgaard contain small accompanying vessels, but for this study, we focus on the large funerary vessels. Frequencies of metalwork (n = 129) and pottery (n = 53) from Aarre, Søhale and Aarupgaard urnfields are sorted by type and illustrated in Fig 10a and 10b. The study includes 15 types of pottery with 14 types at Aarupgaard and 4 types at Søhale, and 15 types of metalwork with 14 types at Aarupgaard, 4 types at Aarre and 7 types at Søhale. There are many more instances of metalwork than pottery, because burials from all three sites contain metalwork, but only at Aarupgaard was pottery preserved well enough to be typologically diagnostic.

The dataset is dominated by burials from Aarupgaard urnfield with more metalwork types and almost all pottery types only coming from this site. This is partly explained by Aarupgaard having a longer duration than Aarre and Søhale, but also better preservation. The differential site contributions have implications for the representations of particularly younger burials in the dataset, as illustrated by comparing frequencies of early pin types (n = 59; pin with type 1 coiled head, pin with type 2 coiled head, pin with circular head) with later occurring types of pins and belt equipment (n = 62; Holstein pin, pin with bomb-shaped head, pin with rod-shaped head, pin with grooved head, winged-head pin, tongue-shaped belt clasp, triangular belt clasp, narrow belt clasp, iron ring with clasp; Fig 10c). Søhale and Aarupgaard contribute with near equal number of burials containing early pin types, whereas the later types of pins and belt equipment, and thereby the dataset, are dominated by burials from Aarupgaard.

### Radiocarbon results

We report 66 new AMS ^14^C ages from Aarupgaard, Aarre and Søhale urnfields, which are compiled in a dataset with previously published ^14^C ages from the same sites, giving a total of 170 ^14^C dates on 111 urnfield burials (Aarre: 12 burials, 46 ^14^C dates; Søhale: 37 burials, 41 ^14^C dates; Aarupgaard: 62 burials, 83 ^14^C dates; Table 1) [15, 22, 105]. The burials are primarily dated on samples of cremated bone, with additional dates on archaeobotanical remains associated with 10 burials from Aarre urnfield. Samples of cremated bone dated in Kiel, Groningen and Brussels have acceptable CI values >5, indicating they were fully cremated and suitable for ^14^C dating. Excepted from this is Aarupgaard U884 (KIA-55391) with CI value 4.8, but because the ^14^C date does not contradict the archaeological prior information we choose to accept the date regardless. There are no available CI values on cremated bone dated in Aarhus and it was not possible to replicate any of the dates. There does however not appear to be no systematic difference between the dates measured in Aarhus and the dates measured in Kiel, Groningen and Brussels. The cremated bone samples have mean values of δ^13^C (Aarre mean = -23.4 ± 2.1 δ^13^C; Søhale mean = -24.8 ± 2.2 δ^13^C; Aarupgaard mean = -23.5 ± 3.0 δ^13^C), and %C (Aarre mean = 0.18 ± 0.09%C; Søhale mean = 0.23 ± 0.13%C; Aarupgaard mean = 0.18 ± 0.10%C) that fall within expected ranges. However, all δ^13^C values are measured by AMS and results will be affected by fractionation during acid extraction, graphitization and AMS measurement, and thus are not comparable between laboratories. Equally, %C can vary between laboratories, because pretreatment methods vary and %C is calculated at different steps in the process [105].

**Table 1:** Radiocarbon results and associated artefacts from Aarupgaard, Aarre and Søhale urnfields. Replicate measurements have been tested for consistency and combined following Ward and Wilson [112]. Osteological information on age and sex for Aarupgaard is based on an unpublished report by L. Harvig from 2019, and for Søhale on Møller et al. [15].

Lab code	Sample ID	Material	Description	Entrances / burial group	CI	%C of extract	Corrected pMC	AMS δ13C (‰VPDB)^1^	^14^C Age (BP)	Reference
**Aarupgaard urnfield cemetery (HAM 1070)**										
KIA-51892	U34x34-1	Cremated bone (human)	17-20y, sex unknown	>2 entrances, M1_CC2,	6.4	0.24	74.68 ± 0.26	-24.4	2346 ± 28	[this study]
			Urn type 20B, 2 pins with type 2 coiled heads							
KIA-51893	U36x36-1	Cremated bone (human)	Teenager/young adult, sex unknown	>2 entrances, M1_CC2	5.9	0.23	73.81 ± 0.19	-24.6	2439 ± 21	[this study]
			Urn type 20B, 2 pins with type 2 coiled heads							
GrM-15072	U51x51-1	Cremated bone (human)	Urn type 15B, 2 pins with coiled heads and incomplete neck bends	>2 entrances, M1_CC1	5.9	0.11	73.77 ± 0.26	-30.3 ± 0.4	2445 ± 20	[105]
KIA-52339	U51x51-1	Cremated bone (human)	-	-	-	0.38	73.73 ± 0.24	-26.8 ± 0.3	2448 ± 26	[105]
	*Weighted mean U51x51-1*: *T’ = 0*.*0*, *T’ (5%) = 3*.*8*, *v = 1*, *2446 ± 16 BP*									
GrM-14589	U81x81-1	Cremated bone (human)	Urn type 12B, 2 pins with type 2 coiled heads, iron chain corroded together with pins	>2 entrances, M1_CC1	5.9	0.06	73.93 ± 0.20	-27.5	2425 ± 30	[105]
KIA-52819	U81x81-81	Replicate of GrM-14589; apatite pretreated at CIO, CO2 extracted and dated at KIA.	-	-	-	0.19	74.37 ± 0.20	-23.0	2379 ± 22	[105]
	*Weighted mean U81x81-81*: *T’ = 1*.*5*, *T’ (5%) = 3*.*8*, *v = 1*, *2395 ± 18 BP*									
GrM-15078	U83x83-1	Cremated bone (human)	Urn type 12B, 2 pins with type 2 coiled heads, pin of unknown type	>2 entrances, M1_CC1	6.0	0.06	73.42 ± 0.30	-28.1	2485 ± 30	[105]
KIA-52825	U83x83-1	Replicate of GrM-15078; apatite pretreated at CIO, CO2 extracted and dated at KIA.	-	-	-	0.17	74.09 ± 0.25	-20.7	2409 ± 27	[105]
	*Weighted mean U83x83-1*: *T’ = 3*.*5*, *T’ (5%) = 3*.*8*, *v = 1*, *2443 ± 21 BP*									
KIA-51894	U123x123-1	Cremated bone (human)	17-24y, sex unknown	>2 entrances, M1_CC2	6.4	0.29	75.32 ± 0.25	-22.6	2277 ± 26	[this study]
			Urn type 20B, triangular belt clasp, pin with circular head							
KIA-51895	U183x183-1	Cremated bone (human)	Urn type 12B, tongue-shaped belt clasp, 2 pins with circular heads	2 entrances, M2_CC1-3	6.6	0.21	75.64 ± 0.21	-23.4	2243 ± 23	[this study]
GrM-14704	U230x230-1	Cremated bone (human)	Urn type 12B, 2 pins with circular heads	>2 entrances, M1_CC2	5.4	0.07	72.83 ± 0.12	-21.7	2546 ± 19	[105]
KIA-52826	U230x230-1	Replicate of GrM-14704; apatite pretreated at CIO, CO2 extracted and dated at KIA.	-	-	-	0.19	74.53 ± 0.23	-20.9	2362 ± 25	[105]
	*Weighted mean U230x230-1*: *T’ = 34*.*1*, *T’ (5%) = 3*.*8*, *v = 1*, *2480 ± 16 BP*									
GrM-14588	U280x280-1	Cremated bone (human)	Urn type 15B, 2 pins with type 2 coiled heads	>2 entrances, M1_CC2	6.2	0.08	74.12 ± 0.14	-25.1	2405 ± 20	[105]
KIA-52820	U280x280-1	Replicate of GrM-14588; apatite pretreated at CIO, CO2 extracted and dated at KIA.	-	-	-	0.27	74.16 ± 0.21	-22.7	2402 ± 22	[105]
	*Weighted mean U280x280-1*: *T’ = 0*.*0*, *T’ (5%) = 3*.*8*, *v = 1*, *2404 ± 15 BP*									
KIA-51896	U293x293-1	Cremated bone (human)	Urn type 20B, 3 pins with circular heads	>2 entrances, M1_CC2	6.9	0.22	74.38 ± 0.21	-22.8	2378 ± 23	[this study]
KIA-51897	U346x346-1	Cremated bone (human)	Urn type 15C, tongue-shaped belt clasp	2 entrances, M2_CC1-3	6.3	0.18	75.61 ± 0.22	-21.9	2246 ± 24	[this study]
GrM-14596	U382x382-1	Cremated bone (human)	Large child/adult, sex unknown	2 entrances, M2_CC1-3	7.3	0.08	75.29 ± 0.14	-24.8 ± 0.2	2280 ± 20	[105]
			Urn type 15C, narrow belt clasp							
KIA-52827	U382x382-1	Replicate of GrM-14596; apatite pretreated at CIO, CO2 extracted and dated at KIA.	-	-	-	0.27	75.77 ± 0.24	-22.5 ± 0.3	2229 ± 25	[105]
	*Weighted mean U382x382-1*: *T’ = 2*.*5*, *T’ (5%) = 3*.*8*, *v = 1*, *2260 ± 16 BP*									
KIA-55388	U427x427-1	Cremated bone (human)	Older adult, sex unknown	2 entrances, M2_CC1-3	6.2	0.26	75.66 ± 0.22	-21.0	2241 ± 17	[this study]
			Holstein pin, triangular belt clasp, tweezer							
KIA-51898	U500x500-1	Cremated bone (human)	Almost adult/adult, sex unknown	>2 entrances, M1_CC3	7.6	0.21	75.30 ± 0.21	-26.7	2278 ± 23	[this study]
			Urn type 12B, 3 pins with circular heads							
GrM-14705	U681x681-1	Cremated bone (human)	Urn type 13D, 2 pins with type 2 coiled heads, simple iron ring	>2 entrances, M1_CC3	6.0	0.08	75.01 ± 0.12	-22.3	2310 ± 19	[105]
KIA-53094	U681x681-1	Replicate of GrM-14705; apatite pretreated at CIO, CO2 extracted and dated at KIA.	-	-	-	0.24	75.05 ± 0.19	-22.5	2305 ± 20	[105]
	*Weighted mean U681x681-1*: *T’ = 0*.*0*, *T’ (5%) = 3*.*8*, *v = 1*, *2308 ± 14 BP*									
RICH-25343	U752x752-1	Cremated bone (human)	Adult, possible male	>2 entrances, M1_CC3	5.7	0.28	74.94	-24.2	2317 ± 26	[this study]
			Urn type 12B, 2 pins with circular heads							
GrM-14589	U766x766-1	Cremated bone (human)	Urn type 15B, 3 pins with circular heads	>2 entrances, M1_CC3	5.5	0.16	75.24 ± 0.14	-26.4	2285 ± 20	[105]
KIA-52821	U766x766-1	Replicate of GrM-14589; apatite pretreated at CIO, CO2 extracted and dated at KIA.	-	-	-	0.39	75.55 ± 0.21	-25.2	2252 ± 23	[105]
	*Weighted mean U766x766-1*: *T’ = 1*.*2*, *T’ (5%) = 3*.*8*, *v = 1*, *2271 ± 16 BP*									
RICH-24151	U797x797-1	Cremated bone (human)	Adult, sex unknown	>2 entrances, M1_CC3	6.7	0.03	75.53	-27.9	2255 ± 28	[this study]
			Urn type 13C, 2 pins with circular heads, narrow belt clasp							
KIA-51899	U858x858-1	Cremated bone (human)	7-16y, sex unknown	2 entrances, M2_CC4	6.2	0.41	75.49 ± 0.21	-21.5	2258 ± 23	[this study]
			Urn type 15B, pin with rod-shaped head, narrow belt clasp							
KIA-55389	U867x867-1	Cremated bone (human)	Urn type 15D, winged head pin	2 entrances, M2_CC4	6.0	0.28	75.93 ± 0.23	-17.9	2219 ± 12	[this study]
KIA-55390	U871x871-1	Cremated bone (human)	Pin with grooved head, iron ring with shank	2 entrances, M2_CC4	5.7	0.35	75.63 ± 0.22	-21.5	2239 ± 17	[this study]
KIA-55391	U884x884-1	Cremated bone (human)	Urn type 15D, winged head pin	2 entrances, M2_CC4	4.8	0.22	75.95 ± 0.21	-21.7	2211 ± 16	[this study]
RICH-24150	U928x928-1	Cremated bone (human)	Almost adult/adult, sex unknown	2 entrances, M1_CC3	7.3	0.05	75.13	-20.6	2297 ± 25	[this study]
			Urn type 15B, pin with type 2 coiled head, pin with circular head							
GrM-14599	U1001x1001-1	Cremated bone (human)	Adult, male	2 entrances, M1_CC4	6.2	0.08	75.71 ± 0.14	-23.0	2235 ± 20	[105]
			Urn types 18C and 15B, pin of unknown type, iron ring with shank							
KIA-53095	U1001x1001-1	Replicate of GrM-14599; apatite pretreated at CIO, CO2 extracted and dated at KIA.	-	-	-	0.22	75.54 ± 0.19	-20.1	2253 ± 21	[105]
	*Weighted mean U1001x1001-1*: *T’ = 0*.*4*, *T’ (5%) = 3*.*8*, *v = 1*, *2244 ± 15 BP*									
KIA-51900	U1016x1016-1	Cremated bone (human)	Adult, sex unknown	2 entrances, M1_CC4	6.7	0.23	75.85 ± 0.24	-23.7	2220 ± 25	[this study]
			Urn type 15B, iron ring with shank							
KIA-55392	U1018x1018-1	Cremated bone (human)	Urn type 15B, winged head pin, iron ring with shank	2 entrances, M1_CC4	5.3	0.21	75.63 ± 0.22	-17.6	2242 ± 17	[this study]
GrM-14592	U1076x1076-1	Cremated bone (human)	16-20y, sex unknown	2 entrances, M1_CC4	6.4	0.08	75.47 ± 0.14	-27.8	2260 ± 20	[105]
			Urn type 18C, narrow belt clasp, bronze neck ring							
KIA-52822	U1076x1076-1	Replicate of GrM-14592; apatite pretreated at CIO, CO2 extracted and dated at KIA.	-	-	-	0.18	76.05 ± 0.28	-25.3	2199 ± 29	[105]
RICH-25340	U1076x1076-1	Cremated bone (human)	-	-		0.12	76.06 ± 0.26	-25.5	2198 ± 27	[105]
	*Weighted mean U1076x1076-1*: *T’ = 4*.*8*, *T’ (5%) = 6*.*0*, *v = 2*, *2229 ± 15 BP*									
GrM-14597	U1186x1186-1	Cremated bone (human)	Urn type 13A, 2 pins with type 1 coiled heads, simple iron ring	>2 entrances, M1_CC1	5.8	0.12	73.58 ± 0.13	-24.7	2465 ± 20	[105]
KIA-53096	U1186x1186-1	Replicate of GrM-14597; apatite pretreated at CIO, CO2 extracted and dated at KIA.	-	-	-	0.27	74.10 ± 0.18	-24.1	2408 ± 20	[105]
	*Weighted mean U1186x1186-1*: *T’ = 4*.*1*, *T’ (5%) = 3*.*8*, *v = 1*, *2437 ± 15 BP*									
RICH-25332	U1232x1232-1	Cremated bone (human)	Urn type 15B, 2 pins with type 1 coiled heads	>2 entrances, M1_CC1	5.8	0.16	73.78	-21.4	2443 ±27	[this study]
RICH-24143	U1279x1279-1	Cremated bone (human)	30+, possible female	>2 entrances, M1_CC1	5.7	0.21	73.56	-19.6	2467 ± 26	[this study]
			Urn type 13A, 2 pins with type 2 coiled heads							
GrM-14602	U1363x1363-1	Cremated bone (human)	Adult, possible male	2 entrances, M1_CC4	6.5	0.07	75.79 ± 0.14	-30.3	2225 ± 20	[105]
			Urn type 15B, triangular belt clasp							
KIA-52828	U1363x1363-1	Replicate of GrM-14602; apatite pretreated at CIO, CO2 extracted and dated at KIA.	-	-	-	0.17	76.09 ± 0.23	-27.9	2195 ± 24	[105]
	*Weighted mean U1363x1363-1*: *T’ = 0*.*9*, *T’ (5%) = 3*.*8*, *v = 1*, *2213 ± 16 BP*									
RICH-25335	U1382x1382-1	Cremated bone (human)	Urn type 18C, 2 pins with circular heads, triangular belt clasp	2 entrances, M1_CC4	6.8	0.10	76.01	-26.2	2204 ± 27	[this study]
RICH-25357	U1422x1422-1	Cremated bone (human)	Urn type 13D, pin with grooved head, narrow belt clasp	2 entrances, M1_CC4	5.9	0.11	76.18	-23.5	2186 ± 26	[this study]
RICH-25333	U1436x1436-1	Cremated bone (human)	Urn type 15C, narrow belt clasp	2 entrances, M1_CC4	5.9	0.11	75.55	-25.3	2252 ± 28	[this study]
RICH-25334	U1617x1617-1	Cremated bone (human)	Urn type 18C, 2 pins with circular heads, bronze pin with grooved head, triangular belt clasp	2 entrances, M1_CC5	5.4	0.14	76.39	-25.6	2163 ± 28	[this study]
KIA-55393	U1654x1654-1	Cremated bone (human)	Pin with grooved head, iron ring with shank	2 entrances, M1_CC5	5.9	0.31	76.22 ± 0.24	-22.0	2183 ±14	[this study]
KIA-52340	U1678x1678-1	Cremated bone (human)	Urn type 15C, narrow belt clasp	2 entrances, M1_CC5	5.4	0.32	75.64 ± 0.23	-22.3	2243 ± 25	[this study]
GrM-15074	U1791x1791-1	Cremated bone (human)	Urn type 15C, triangular belt clasp	2 entrances, M1_CC5	5.7	0.07	75.78 ± 0.26	-30.0	2230 ± 25	[105]
KIA-52341	U1791x1791-1	Cremated bone (human)	-	-	-	0.29	76.35 ± 0.23	-29.63	2167 ± 25	[105]
	*Weighted mean U1791x1791-1*: *T’ = 3*.*2*, *T’ (5%) = 3*.*8*, *v = 1*, *2199 ± 18 BP*									
RICH-24144	U1834x1834-1	Cremated bone (human)	25-50y, possible male	2 entrances, M1_CC5	6.5	0.09	74.89	-19.8	2322 ± 25	[this study]
			Urn type 12B, tongue-shaped belt clasp							
KIA-55394	U1834x1834-1	Cremated bone (human), separate sample from RICH-24144	-	-	5.1	0.18	75.91 ± 0.23	-25.7	2216 ± 18	[this study]
	*Weighted mean U1834x1834-1*: *T’ = 11*.*9*, *T’ (5%) = 3*.*8*, *v = 1*, *2253 ± 15 BP*									
GrM-15075	U1847x1847-1	Cremated bone (human)	Urn type 15C, triangular belt clasp	2 entrances, M1_CC5	6.1	0.08	75.53 ± 0.20	-25.4	2255 ± 20	[105]
KIA-52342	U1847x1847-1	Cremated bone (human)	-	-	-	0.28	75.78 ± 0.24	-28.9	2228 ± 25	[105]
	*Weighted mean U1847x1847-1*: *T’ = 0*.*7*, *T’ (5%) = 3*.*8*, *v = 1*, *2244 ± 16 BP*									
KIA-55395	U1894x1894-1	Cremated bone (human)	Pin with rod-shaped head, iron ring with shank	2 entrances, M1_CC5	5.1	0.24	75.94 ± 0.21	-21.1	2211 ± 14	[this study]
KIA-52343	U1970x1970-1	Cremated bone (human)	Urn type 15C, bronze pin with grooved head, narrow belt clasp	2 entrances, M1_CC5	5.9	0.28	75.61 ± 0.22	-22.8	2246 ± 23	[this study]
KIA-55396	U1993x1993-1	Cremated bone (human)	Pin with grooved head, iron ring with shank	2 entrances, M1_CC5	5.1	0.35	75.82 ± 0.25	-21.6	2228 ±15	[this study]
KIA-52344	U1997x1997-1	Cremated bone (human)	Urn type 18C, bronze pin with grooved head, triangular belt clasp	2 entrances, M1_CC5	6.5	0.38	76.36 ± 0.26	-30.6	2167 ± 27	[this study]
KIA-55397	U2199x2199-1	Cremated bone (human)	Holstein pin	2 entrances, M1_CC6	5.3	0.15	75.59 ± 0.26	-20.4	2244 ± 17	[this study]
GrM-14593	U2262x2262-1	Cremated bone (human)	Large child/young adult, sex unknown	2 entrances, M1_CC6	6.5	0.07	75.53 ± 0.16	-20.9	2255 ± 25	[105]
			Urn type 11D, narrow belt clasp							
KIA-52823	U2262x2262-1	Replicate of GrM-14593; apatite pretreated at CIO, CO2 extracted and dated at KIA.	-	-	-	0.24	75.92 ± 0.26	-19.0	2214 ± 28	[105]
	*Weighted mean U2262x2262-1*: *T’ = 1*.*2*, *T’ (5%) = 3*.*8*, *v = 1*, *2237 ± 19 BP*									
KIA-55398	U2293x2293-1	Cremated bone (human)	Urn type 15D, pin with rod-shaped head, iron ring with shank	2 entrances, M1_CC6	6.7	0.05	76.19 ± 0.31	-22.3	2185 ± 25	[this study]
KIA-55399	U2354x2354-1	Cremated bone (human)	Urn type 15C, pin with rod-shaped head, iron ring with shank	2 entrances, M1_CC6	7.9	0.12	75.70 ± 0.23	-22.6	2237 ± 18	[this study]
KIA-55400	U2366x2366-1	Cremated bone (human)	Urn type 20B, pin with rod-shaped head, simple iron ring	2 entrances, M1_CC6	5.8	0.22	75.75 ± 0.24	-18.9	2232 ± 15	[this study]
KIA-55401	U2455x2455-1	Cremated bone (human)	Pin with rod-shaped head, simple iron ring	2 entrances, M1_CC6	7.4	0.05	75.66 ± 0.30	-21.5	2240 ± 25	[this study]
KIA-55402	U2498x2498-1	Cremated bone (human)	Urn type 15D, winged head pin	2 entrances, M1_CC6	7.7	0.08	76.37 ± 0.31	-24.5	2165 ± 25	[this study]
RICH-24145	U2541x2541-1	Cremated bone (human)	Adult, sex unknown	2 entrances, M1_CC6	5.9	0.06	75.89	-22.7	2216 ± 26	[this study]
			Urn type 20D, Holstein pin, pin with flat pierced head, iron ring with shank,							
KIA-55403	U2545x2545-1	Cremated bone (human)	Pin with rod-shaped head, pin with small pierced head	2 entrances, M1_CC6	6.1	0.15	76.11 ± 0.24	-24.9	2194 ± 18	[this study]
RICH-24146	U2550x2550-1	Cremated bone (human)	Large child/adult, sex unknown	2 entrances, M1_CC6	5.9	0.08	75.18	-22.3	2291 ± 26	[this study]
			Urn type 15D, iron ring with shank							
KIA-55404	U2593x2593-1	Cremated bone (human)	Urn type 17D, Holstein pin, iron ring with shank	2 entrances, M1_CC6	6.9	0.15	75.84 ± 0.23	-21.9	2222 ± 17	[this study]
RICH-25355	U2710x2710-1	Cremated bone (human)	Urn type 11A, pin with bomb-shaped head, possibly second pin of same type, unknown pin type with bend neck	>2 entrances, M1_CC1	5.5	0.12	74.07	-18.9	2411 ± 27	[this study]
GrM-14594	U3330x3330-1	Cremated bone (human)	Almost adult/adult, sex unknown	Founding burial	6.8	0.10	72.96 ± 0.13	-21.0	2535 ± 20	[105]
			Urn type 15? Iron pin with coiled head and no bend neck							
KIA-52824	U3330x3330-1	Replicate of GrM-14594; apatite pretreated at CIO, CO2 extracted and dated at KIA.	-	-	-	0.29	73.52 ± 0.24	-21.5	2471 ± 26	[105]
KIA-51901	U3330x3330-1	Cremated bone (human), separate sample from KIA-52824 and GrM14594	-	-	-	0.28	73.23 ± 0.24	-21.9	2503 ± 27	[105]
	*Weighted mean U3330x3330-1*: *T’ = 3*.*9*, *T’ (5%) = 6*.*0*, *v = 2*, *2509 ± 14 BP*									
RICH-25354	U3341x3341-1	Cremated bone (human)	Urn of Bronze Age period VI type, side urn of pre-Roman Iron Age style, swan neck pin	Founding burial	5.7	0.35	73.47	-21.5	2477±27	[this study]
RICH-24147	U3452x3452-1	Cremated bone (human)	Adult, possible male	>2 entrances, M1_CC1	6.2	0.12	73.03	-19.1	2525 ± 25	[this study]
			Urn type 15B, 2 pins with type 1 coiled heads							
RICH-24148	U3778x3778-1	Cremated bone (human)	Urn type 12B, 2 pins with type 2 coiled heads, simple iron ring	>2 entrances, M1_CC1	5.4	0.12	73.7	-18.1	2452 ± 25	[this study]
KIA-52345	U3822x3822-1	Cremated bone (human)	Urn type 11B, 2 pins with type 1 coiled heads	>2 entrances, M2_CC1-3	5.5	0.26	73.87 ± 0.24	-23.9	2433 ± 26	[this study]
GrM-15076	U3869x3869-1	Cremated bone (human)	Almost adult/adult, sex unknown	Founding burial	6.0	0.09	72.87 ± 0.16	-21.6	2540 ± 20	[105]
			Urn, open with no neck. Iron pin with round head and no neck bend							
KIA-52346	U3869x3869-1	Cremated bone (human)	-	-	-	0.21	73.66 ± 0.23	-25.9	2456 ± 25	[105]
RICH-24152	U3869x3869-1	Cremated bone (human)	-	-	-	0.20	73.22 ± 0.24	-25.8	2504 ± 26	[105]
	*Weighted mean U3869x3869-1*: *T’ = 6*.*9*, *T’ (5%) = 6*.*0*, *v = 2*, *2507 ± 14 BP*									
**Aarre urnfield cemetery (VAM 1600)**										
KIA-53941	A86x339 (urn)	*Acer* sp. trunk wood charcoal (Ø >10 cm)	-	-	-	63.96	73.60 ± 0.23	-25.3	2463 ± 25	[22]
KIA-53942	A86x340 (urn)	Cremated bone (human)	-	-	5.4	0.35	74.37 ± 0.24	-19.7	2379 ± 26	[22]
KIA-53942	A86x340 (urn)	Cremated bone (human), replicate	Urn of unknown type, 2 pins with type 2 coiled heads	-	-	-	74.31 ± 0.23	-22.8	2385 ± 25	[22]
	*Weighted mean A86x340*: *T’ = 0*.*0*, *T’ (5%) = 3*.*8*, *v = 1*, *2382 ± 19 BP*									
RICH-25356	A89x311 (urn)	Cremated bone (human)	Urn type 13, 2 pins with type 1 coiled heads	-	6.4	0.16	-	-21.18	2464 ± 27	[this study]
KIA-53984	A95x368 no.1 (urn)	*Quercus* sp. twig charcoal (Ø <0.3 cm)	-	-	-	70.68	74.45 ± 0.23	-28.4	2370 ± 25	[22]
KIA-53985	A95x368 no.3 (urn)	*Acer* sp. twig charcoal (Ø <0.5 cm)	-	-	-	70.49	73.90 ± 0.23	-27.7	2430 ± 26	[22]
RICH-25342	A95x369 (urn)	Cremated bone (human)	Urn type 16?	-	7.8	0.11	73.90 ± 0.25	-24.3	2428 ± 27	[22]
RICH-25071	A99x65 no.2 (pit)	*Alnus* sp. trunk wood charcoal (Ø 8–10 cm)	-	-	-	60.00	75.39 ± 0.28	-33.3	2269 ± 29	[22]
RICH-25067	A99x65 no.3 (pit)	*Quercus* sp. trunk wood charcoal (Ø <10 cm)	-	-	-	61.00	67.85 ± 0.26	-31.6	3115 ± 31	[22]
GrM-16774	A99x345 (urn)	Cremated bone (human)	Urn of unknown type, pin with type 1 coiled head	-	6.5	0.06	75.50 ± 0.17	-26.5	2255 ± 20	[22]
RICH-25069	A99x346 no.1 (urn)	*Alnus* sp. twig charcoal (Ø <0.3 cm)	-	-	-	53.50	77.14 ± 0.28	-31.8	2085 ± 29	[22]
RICH-25066	A99x346 no.27 (urn)	Alnus sp. trunk wood charcoal (Ø > 10 cm)	-	-	-	61.60	75.56 ± 0.28	-35.3	2251 ± 30	[22]
GrM-14604	A117x762 (urn)	Cremated bone (human)	Urn type 12, elaborate set of chain and pins	-	6.0	0.10	73.75 ± 0.13	-25.7	2445 ± 20	[105]
KIA-53098	A117x762 (urn)	Replicate of GrM-14604; apatite pretreated at CIO, CO2 extracted and dated at KIA.	-	-	-	0.28	74.03 ± 0.19	-22.1	2416 ± 20	[105]
	*Weighted mean A117x762*: *T’ = 1*.*1*, *T’ (5%) = 3*.*8*, *v = 1*, *2431 ± 15 BP*									
KIA-53943	A117x769 (urn)	*Quercus* sp. charcoal (Ø >10 cm) 1 annual ring sampled	-	-	-	31.21	73.72 ± 0.23	-23.6	2449 ± 25	[22]
KIA-53944	A117x774 (urn)	*Quercus* sp. charcoal (Ø >10 cm)	-	-	-	60.58	73.30 ± 0.22	-25.0	2495 ± 24	[22]
KIA-53945	A130x82 no.1 (urn)	Charred grass stem	Pin of unknown type, tongue-shaped belt clasp,	-	-	68.30	72.49 ± 0.23	-25.1	2585 ± 25	[22]
KIA-53946	A130x82 no.2 (urn)	*Triticum* cf. *aestivum*, charred cereal	-	-	-	64.19	76.46 ± 0.23	-28.5	2156 ± 24	[22]
KIA-53947	A130x217 (urn)	Cremated bone (human)	-	-	5.8	0.24	75.57 ± 0.23	-20.9	2250 ± 25	[22]
KIA-53947	A130x217 (urn)	Cremated bone (human), replicate	-	-	-	-	75.52 ± 0.23	-21.7	2255 ± 25	[22]
	*Weighted mean A130x217*: *T’ = 0*.*0*, *T’ (5%) = 3*.*8*, *v = 1*, *2253 ± 18 BP*									
KIA-53948	A155x127 no.1 (urn)	cf. *Triticum* sp., charred cereal	-	-	-	50.00	73.31 ± 0.22	-26.4	2494 ± 24	[22]
KIA-53949	A155x127 no.2 (urn)	*Arrhenatherum elatius* ssp. *Bulbosum*, charred grass bulb	-	-	-	65.52	73.56 ± 0.22	-28.2	2466 ± 24	[22]
KIA-53950	A155x281 (urn)	Cremated bone (human)	Pin with circular head	-	6.1	0.26	74.55 ± 0.23	-23.6	2359 ± 25	[22]
KIA-53950	A155x281 (urn)	Cremated bone (human), replicate	-	-	-	-	74.41 ± 0.23	-24.1	2374 ± 25	[22]
	*Weighted mean A155x281*: *T’ = 0*.*2*, *T’ (5%) = 3*.*8*, *v = 1*, *2367 ± 18 BP*									
KIA-53951	A198x338 (urn)	*Alnus* sp. trunk wood charcoal (Ø >10 cm)	-	-	-	57.02	69.12 ± 0.21	-24.9	2967 ± 24	[22]
KIA-53952	A198x338 CB (urn)	Cremated bone (human)	Urn of unknown type, 2 pins with type 2 coiled heads	-	5.9	0.28	74.89 ± 0.24	-24.9	2323 ± 26	[22]
KIA-53952	A198x338 CB (urn)	Cremated bone (human), replicate	-	-	-	-	74.82 ± 0.29	-25.9	2330 ± 35	[22]
	*Weighted mean A198x338 CB*: *T’ = 0*.*0*, *T’ (5%) = 3*.*8*, *v = 1*, *2325 ± 21 BP*									
GrM-14707	A281x484 (urn)	Cremated bone (human)	Urn type 15, 2 pins with circular heads	-	6.2	0.10	74.91 ± 0.13	-28.0	2320 ± 20	[105]
KIA-53100	A281x484 (urn)	Replicate of GrM-14707; apatite pretreated at CIO, CO2 extracted and dated at KIA.		-	-	0.11	75.37 ± 0.19	-23.5	2271 ± 20	[105]
	*Weighted mean A281x484*: *T’ = 3*.*9*, *T’ (5%) = 3*.*8*, *v = 1*, *2296 ± 15 BP*									
KIA-53953	A278x782 no.1 (urn)	Charred grass stem	-	-	-	74.57	74.17 ± 0.23	-27.5	2400 ± 25	[22]
KIA-53954	A278x782 no.2 (urn)	Charred grass, stem and root fragment	-	-	-	67.11	73.76 ± 0.23	-26.2	2445 ± 25	[22]
KIA-53955	A278x783 (urn)	Cremated bone (human)	Urn of unknown type, 2 pins with disc-shaped heads, bronze ring	-	5.5	0.20	73.47 ± 0.24	-22.7	2477 ± 26	[22]
KIA-53955	A278x783 (urn)	Cremated bone (human), replicate	-	-	-	-	73.71 ± 0.23	-22.9	2450 ± 25	[22]
	*Weighted mean A278x783*: *T’ = 0*.*6*, *T’ (5%) = 3*.*8*, *v = 1*, *2463 ± 19 BP*									
RICH-25068	A393x568 no.1 (pit)	*Triticum dicoccum*, charred cereal	-	-	-	61.10	69.69 ± 0.28	-29.9	2901 ± 32	[22]
RICH-25070	A393x568 no.2 (pit)	*Hordeum vulgare nudum*, charred cereal	-	-	-	44.30	69.58 ± 0.28	-27.2	2914 ± 32	[22]
RICH-25341	A393x561 CB (urn)	Cremated bone (human)	Urn of unknown type, 2 pins with type 1 coiled heads	-	6.4	0.16	73.90 ± 0.25	-25.3	2480 ± 27	[22]
KIA-52411	A393x561 no.1 (urn)	*Hordeum vulgare nudum*, charred cereal	-	-	-	54.67	67.70 ± 0.21	-24.0	3134 ± 25	[22]
KIA-52412	A393x561 no.3 (urn)	*Hordeum vulgare nudum*, charred cereal	-	-	-	32.35	67.57 ± 0.22	-21.9	3150 ± 27	[22]
KIA-52413	A393x561 *Quercus* (urn)	*Quercus* sp. trunk wood charcoal (Ø >10 cm)	-	-	-	24.78	72.25 ± 0.24	-25.7	2611 ± 27	[22]
KIA-52414	A394x556 no.1 (urn)	*Alnus* sp. charcoal from branch sapwood (Ø 3–5 cm)	-	-	-	26.74	70.77 ± 0.23	-24.9	2778 ± 27	[22]
KIA-53983	A394x781 no.9 (urn)	*Acer* sp. trunk wood charcoal (Ø > 10 cm)	-	-	-	66.85	68.58 ± 0.21	-27.0	3029 ± 24	[22]
GrM-14708	A394x785 CB (urn)	Cremated bone (human)	Urn of unknown type, 2 pins with type 2 coiled heads	-	6.3	0.10	73.57 ± 0.11	-21.2	2465 ± 18	[105]
KIA-53099	A394x785 CB (urn)	Replicate of GrM-14708; apatite pretreated at CIO, CO2 extracted and dated at KIA.	-	-	-	0.14	73.97 ± 0.19	-22.7	2422 ± 20	[105]
	*Weighted mean A394x785 CB*: *T’ = 2*.*6*, *T’ (5%) = 3*.*8*, *v = 1*, *2446 ± 14 BP*									
KIA-52415	A394x785 no.1 (urn)	*Triticum aestivum*, charred cereal	-	-	-	37.76	70.19 ± 0.23	-23.0	2843 ± 26	[22]
KIA-52416	A394x785 no.4 (urn)	*Alnus* sp. heartwood charcoal (Ø 2–3 cm)	-	-	-	28.07	70.82 ± 0.23	-27.9	2772 ± 26	[22]
KIA-52417	A394x786 no.1 (urn)	*Quercus* sp. trunk wood charcoal (Ø >10 cm)	-	-	-	51.01	71.29 ± 0.24	-25.2	2719 ± 27	[22]
**Søhale urnfield cemetery (ESM 2139)**										
AAR-25251	G3x22-III	Cremated bone (human)	Almost adult/adult, sex unknown	Multiple entrances	-	-	75.94 ± 0.26	-25	2211 ± 27	[15]
			Urn of unknown type, unknown iron object							
AAR-25250	G5x21-III	Cremated bone (human)	Adult (25-40y), possible female	Multiple entrances	-	-	75.5 ± 0.25	-27	2258 ± 27	[15]
			Belt clasp?							
KIA-53937	G7x37-III	Cremated bone (human)	Adult (>40y), possible female	NNE-SSW entrances	5.1	0.38	75.86 ± 0.23	-21.9	2220 ± 20	[15, this study]
			Urn type 13C, pin with circular head							
KIA-53936	G9x19-II	Cremated bone (human)	Nearly adult/adult, possible male	NNE-SSW entrances	6.2	0.23	75.43 ± 0.24	-25.8	2265 ± 26	[15, this study]
			2 pins with large circular heads							
RICH-26501	G10x23-II	Cremated bone (human)	Adult, possible male	NNE-SSW entrances	-	0.10	74.97 ± 0.23	-22.8	2314 ± 24	[15, this study]
			Urn of unknown type, 2 pins with circular head							
AAR-25249	G11x18-III	Cremated bone, (human)	Child (6-16y), sex unknown	N-S entrances	-	-	76.19 ± 0.26	-28	2185 ± 27	[15]
			Pin of unknown type, narrow belt clasp							
AAR-25248	G12x17	Cremated bone (human)	Large child/adult, sex unknown	NNE-SSW entrances	-	-	75.46 ± 0.37	-26	2262 ± 40	[15]
			No burial goods							
AAR-25243	G19x10-II	Cremated bone, (human)	Child (2-10y), sex unknown	NNE-SSW entrances	-	-	75.93 ± 0.29	-24	2212 ± 30	[15]
			Urn of unknown type							
AAR-25244	G31x12/x25A	Cremated bone (human)	Almost adult/adult, sex unknown	NNE-SSW entrances	-	-	76.22 ± 0.36	-29	2181 ± 38	[15]
			No burial goods							
AAR-25245	G31x12/x25B	Cremated bone (human)	-	-	-	-	75.74 ± 0.32	-24	2232 ± 34	[15]
	*Weighted mean G31x12*: *T’ = 1*.*0*, *T’ (5%) = 3*.*8*, *v = 1*, *2209 ± 26 BP*									
AAR-25246	G32x14	Cremated bone (human)	Adult (>16y), sex unknown	NNE-SSW entrances	-	-	74.74 ± 0.25	-23	2339 ±26	[15]
			Urn type 15C							
GrM-16770	G34x40-II	Cremated bone (human)	Child (6-14y), sex unknown	?-SSW entrances	-	0.10	75.79 ± 0.18	-23.5	2227 ± 19	[15, this study]
			Urn of unknown type, pin with circular head							
RICH-26493	G35x28-II	Cremated bone (human)	No info on age or sex	NNE-SSW entrances	-	0.26	75.62 ± 0.25	-26.3	2245 ± 27	[15, this study]
			Urn of unknown type, tongue-shaped belt clasp							
AAR-25253	G37x27-I	Cremated bone (human)	Large child/adult (>12y), sex unknown	NNE-S entrances	-	-	75.7 ± 0.28	-24	2237 ± 30	[15]
			No burial goods							
RICH-26494	G39x31-II	Cremated bone (human)	Large child/adult, sex unknown	NNE-SSW entrances	-	0.10	74.76 ± 0.25	-24.6	2337 ±27	[15, this study]
			Urn type 15? Pin with circular head, pin with bend neck							
AAR-25254	G40x30-III	Cremated bone (human)	Large child/adult (>15y), sex unknown	NNE-SSW entrances	-	-	74.9 ± 0.26	-26	2322 ± 28	[15]
			Eyelet ring, triangular belt clasp							
RICH-26495	G44x38-II	Cremated bone (human)	Adult (>25y), possible female	NNE-SSW entrances	5.9	0.06	75.54 ± 0.24	-28.8	2254 ± 26	[15, this study]
			Urn type 15, narrow belt clasp							
KIA-53938	G44x38-II	Replicate of RICH-26495	-	-	-	0.22	76.12 ± 0.25	-21.5	2192 ± 27	[this study]
	*Weighted mean*: *G44x38-II*: *T’ = 2*.*7*, *T’ (5%) = 3*.*8*, *v = 1*, *2224 ± 19 BP*									
AAR-25257	G48x35-II	Cremated bone (human)	Adult (>20y), possible male	N entrance	-	-	73.54 ± 0.24	-26	2469 ± 26	[15]
			Urn type 12C							
AAR-25258	G51x47-III	Cremated bone (human)	Adult (>40y), possible male	NNW-S entrances	-	-	73.98 ± 0.25	-19	2421 ± 27	[15]
			Urn type 15, 2 pins with type 1 coiled heads							
AAR-25256	G52x33-II	Cremated bone (human)	Adult (>35y), possible male	NNE-SSE entrances	-	-	74.61 ± 0.25	-24	2353 ± 27	[15]
			Urn of unknown type							
RICH-26502	G53x34-II	Cremated bone (human)	Adult, possible female	NNE-SSE entrances	-	0.37	74.71 ± 0.24	-25.8	2342 ± 26	[15, this study]
			Urn of unknown type, 2 pins with circular head							
AAR-25255	G54x32-II	Cremated bone (human)	Adult (>35y), sex unknown	NNE-SSW entrances	-	-	74.15 ± 0.27	-29	2403 ± 29	[15]
			Urn of unknown type, 2 pins with circular head							
GrM-16771	G57x41-V	Cremated bone (human)	Adult (>25y), possible male	NNE-SSW entrances	-	0.09	74.46 ± 0.19	-25.8	2370 ± 20	[15, this study]
			Urn 13C, narrow belt clasp, 1 pin with circular head, 1 pin of unknown type							
GrM-16772	G59x44-II	Cremated bone (human)	Adult, possible male	No entrances	5.8	0.09	73.60 ±0.20	-26.5	2465 ±20	[15, this study]
			2 pins with coiled head, type 1 and 2							
KIA-53939	G59x44-II	Replicate of GrM-16772	-	-	-	0.34	73.55 ± 0.23	-24.1	2468 ± 25	This study
	*Weighted mean G59x44-II*: *T’ = 0*.*0*, *T’ (5%) = 3*.*8*, *v = 1*, *2466 ± 16 BP*									
GrM-16773	G60x49-III	Cremated bone (human)	Adult (>35y), sex unknown	No entrances	-	0.15	73.92 ±0.16	-25.5	2425 ±19	[15, this study]
			Urn of unknown type, pin with type 1 coiled head, bronze ring with eyelet							
AAR-25260	G63x51-IV	Cremated bone (human)	Adult (>35y), possible male	No entrances	-	-	73.8 ± 0.25	-25	2440 ± 27	[15]
			Urn type 15C, pin with type 1 coiled head, pin of unknown type							
KIA-53431	G64x50-II	Cremated bone (human)	Adult, possible male	No entrances	5.5	0.29	73.82 ±0.19	-21.9	2438 ±21	[15, this study]
			Urn type 15B, pin with type 2							
			coiled head, fragments of a pin of unknown type							
AAR-25261	G69x52-II	Cremated bone (human)	Large child/adult (12-18y), sex unknown	No entrances	-	-	74.01 ± 0.28	-21	2418 ± 31	[15]
			Urn type 15C, pin with type 1 coiled head, minimum 2 pins with neck bend							
AAR-25262	G70x55-II	Cremated bone (human)	Adult (>35y), possible male	N-E entrances	-	-	74.68 ± 0.26	-26	2345 ± 27	[15]
			Urn type 13							
KIA-53435	G73x92-III	Cremated bone (human)	Adult (>25y), possible male	No entrances?	6.0	0.39	73.93 ±0.19	-24.2	2427 ±21	[15, this study]
			Urn of unknown type, pin with type 1 coiled head, pin with bend neck and head, imitation of pin x92-I?							
AAR-25263	G81x65-V	Cremated bone (human)	Adult (>35y), possible female	N-S entrances	-	-	74.97 ± 0.26	-18	2314 ± 28	[15]
			Urn of unknown type, 2 pins with slightly coiled heads, iron ring with shank							
AAR-25265	G82x93-II	Cremated bone (human)	Adult (>25y), possible male	?-S entrance(s)	-	-	74.3 ± 0.27	-24	2387 ± 29	[15]
			Urn type 15, 2 pins, hereof at least one with type 2 coiled head							
AAR-25264	G87x69-IV	Cremated bone (human)	Adult (>25y), possible male	NNE-SSW entrances	-	-	75.08 ± 0.27	-26	2303 ± 28	[15]
			Urn of unknown type, pin with circular head, pin with neck bend							
AAR-25252	G93x26-III	Cremated bone (human)	Large child/adult (>15y), sex unknown	Multiple entrances	-	-	75.32 ± 0.36	-20	2277 ± 38	[15]
			Pin with small circular head, tongue-shaped belt clasp							
KIA-53434	G105x76-II	Cremated bone (human)	Child (7-11y), sex unknown	No burial mound	6.0	0.24	73.86 ±0.19	-29.0	2434 ±21	[15, this study]
			Urn type 15C, pin with type 2 coiled head, pin with type 1 coiled head							
KIA-53940	G105x76-II	Replicate of KIA-53434	-	-	-	0.21	73.90 ± 0.24	-27.4	2429 ± 26	[this study]
	*Weighted mean G105x76-II*: *T’ = 0*.*0*, *T’ (5%) = 3*.*8*, *v = 1*, *2432 ± 17 BP*									
KIA-53433	G107x74-II	Cremated bone (human)	Child (10-14y), sex unknown	No burial mound	5.9	0.33	74.42 ±0.19	-22.6	2373 ±21	[15, this study]
			Urn of unknown type, pin with type 2 coiled head, pin with type 1 coiled head							
KIA-53432	G110x72-VII	Cremated bone (human)	Almost adult/adult, sex unknown Urn of unknown type, flat iron ring, 2 pins with type 2 coiled heads	No burial mound	6.4	0.44	74.34 ±0.19	-23.1	2382 ±21	[15, this study]
AAR-25259	G111x48-II	Cremated bone (human)	Almost adult/adult, sex unknown Urn of unknown type	N-S entrances	-	-	73.62 ± 0.27	-26	2460 ± 30	[15]

Replicate measurements are available on 17 sets of double targets and on separate extractions of samples from 26 burials. The 17 sets of double targets are all highly statistically consistent with mean T-value 0.01 [112]. 21 paired results on separate extractions are statistically consistent at 95% confidence and another two paired results are close to being statistically consistent and we chose to accept them (Aarupgaard 1186: (T = 4.1, T’ (5%) = 3.8, v = 1); Aarre A281: (T = 3.9, T’ (5%) = 3.8, v = 1; Table 1). The three available dates on Aarupgaard U3869 are not in agreement (T = 6.9, T’ (5%) = 6.0, v = 1), although the two earlier dates and the two later dates are, respectively. It is difficult to conclude whether this is caused by a slight underestimation of errors, of if there is a real difference between the 14C ages of the extracts [105]. We cannot reject any of the dates and choose to accept the combined date regardless. The two remaining paired results are highly statistically inconsistent (Aarupgaard U230: (T = 34.1, T’ (5%) = 3.8, v = 1); Aarupgaard U1834: (T = 11.9, T’ (5%) = 3.8, v = 1), but there is no reason to dismiss any of the dates based on values of CI, %C yield or δ^13^C. Instead, we evaluate the results based on the expected time range of the accompanying artefacts. Aarupgaard U230 contains pins with circular head and Aarupgaard U1834 contains a tongue-shaped belt clasp. In both cases, we reject the earlier date (GrM-14704 and RICH-24144, respectively) because they are considerably earlier than other dates on these artefact types. Results from Aarupgaard U1834 were measured on separate bone fragments, which might have different wood-age offsets due to differential uptake of carbon from the burning atmosphere [22], although the presence of multiple individuals in the grave cannot be excluded, as observed in Late Bronze Age cremation burials in Belgium [113]. The laboratories have participated in more intercomparison studies, demonstrating results to be reproducible and comparable [105, 114].

## Chronological modelling

All dates are calibrated in OxCal v4.4 using the IntCal20 calibration curve [51, 65]. All cremated bone samples gave ^14^C ages between c.2550 and 2150 BP, equivalent to c. six centuries in the middle of the first millennium BC [51, 65]. The ^14^C dataset consists predominantly of dates measured on cremated bone, which have been demonstrated to be susceptible to wood-age offsets [19–20, 22, 115]. Modelling the cremated bone dates as simple *terminus post quem* (*TPQ)* is unhelpful and instead a Cremation Outlier_Model (OM) is applied to all dates on cremated bone [22]. The Cremation OM is based on an existing OxCal OM for charcoal [116, 117], but the parameter values are changed to incorporate a minimum offset, a slightly faster exponential decay and a reduction of sub-decadal offsets. Chronological models discussed in the following are provided in S2 File using the exact code in OxCal v4’s Chronological Query Language [65].

### Modelling burial activity

Initial modelling of burial activity at Aarre, Aarupgaard and Søhale urnfields imposes no constraints of the order of dated samples. Excepted from this are dates from Aarre urnfield on charcoal with potentially significant intrinsic age that are used as *TPQ*s of the associated burial, and where cremated bone dates from burials A95, A130 and A278 are combined with dates on contemporaneous, short-lived samples [22]. Legacy dates from LBA periods V and VI [118] are modelled in two contiguous phases and the end boundary of period VI is used as *TPQ* to constrain the start of the urnfield model. The initial **urnfield model A** is compatible with the ^14^C results (A_overall_ = 80.5), and it estimates urnfield burials started *713–529 cal BC* (95.4% probability) and ended *212 cal BC– 47 cal AD* (95.4% probability). The model provides multimodal solutions for many burials, and estimated a much shorter use-life at Søhale than is indicated by typo-chronology. Based on this we reject the initial urnfield model A.

There are very limited stratigraphic relationships between urnfield burials that can be applied as dating constraints in a chronological model, although it has been discussed if urnfields might be described using horizontal stratigraphy [7, 88]. To check the plausibility of these interpretations, we modified the basic structure of model A by adding the inferred site formation processes as dating constraints (urnfield model B). Søhale urnfield are modelled in three potentially overlapping bounded phases, based the orientation of interruptions of the circular ditches, which was thought to have changed over time: from no interruptions, followed by N-S orientated interruptions to NNE-SSW orientated interruptions [15]. This interpretation was at least partly based on ^14^C dating and we refrain from defining the sequence of entrance groups to avoid circular reasoning. Burials from Aarupgaard urnfield are modelled in a sequence based on typo-chronology with an initial ‘founding phase’ with burials U3330, U3341 and U3869 that were interred within an existing Bronze Age mound. Their funerary urns resemble LBA pottery, and although the pins are made of iron, they are typologically closer to LBA types. Remaining burials are modelled based on observations that the number of interruptions of the circular ditches changed over time [75, 88], starting with a phase with multiple interruptions, followed by a final phase with two interruptions. The transition between the latter two phases is modelled with trapezium priors, allowing it to have a duration. There is no architectural prior information available on the internal sequence of the burials from Aarre urnfield.

**Fig 11:**
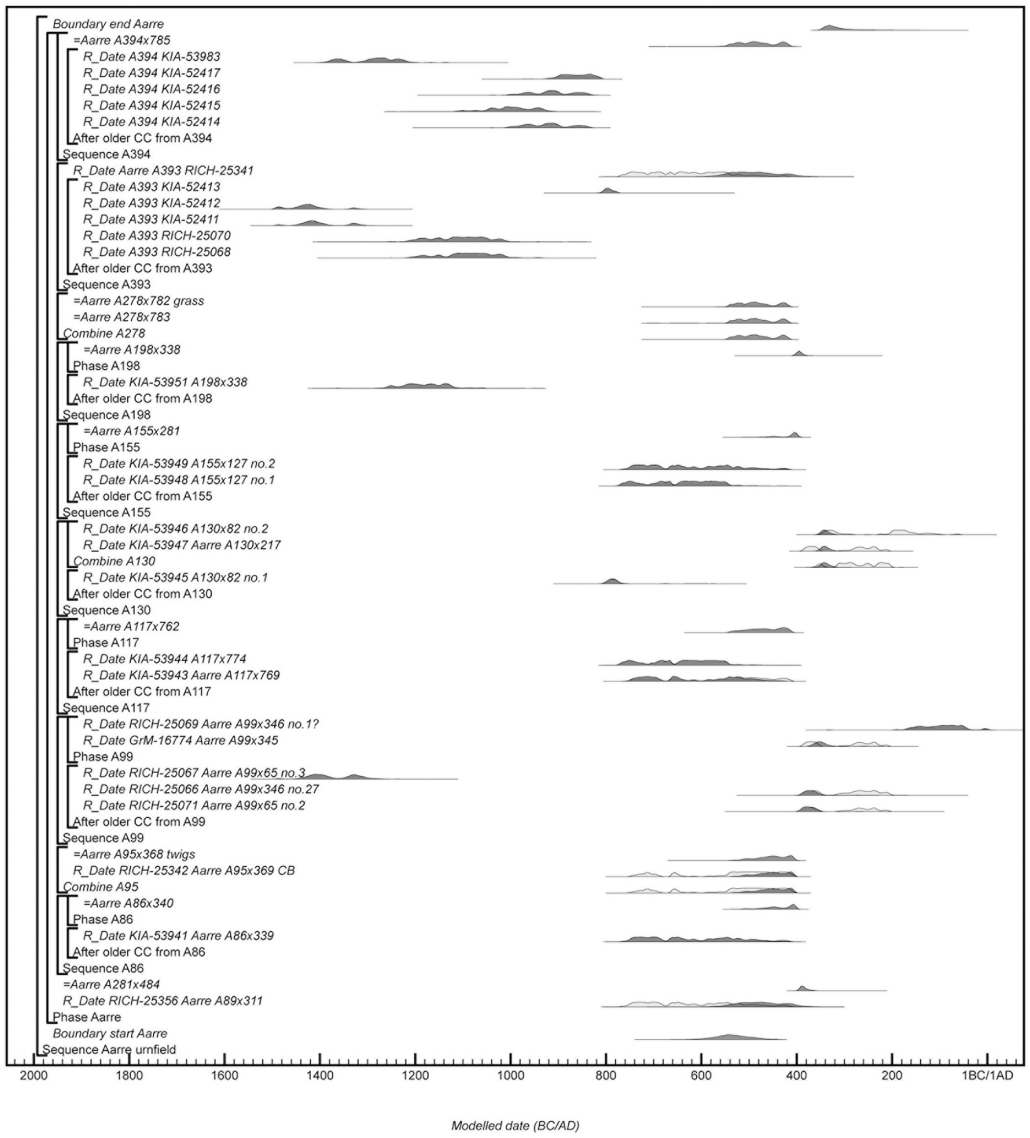
Chronological model of Aarre urnfield, based on the preferred urnfield model B.

**Fig 12:**
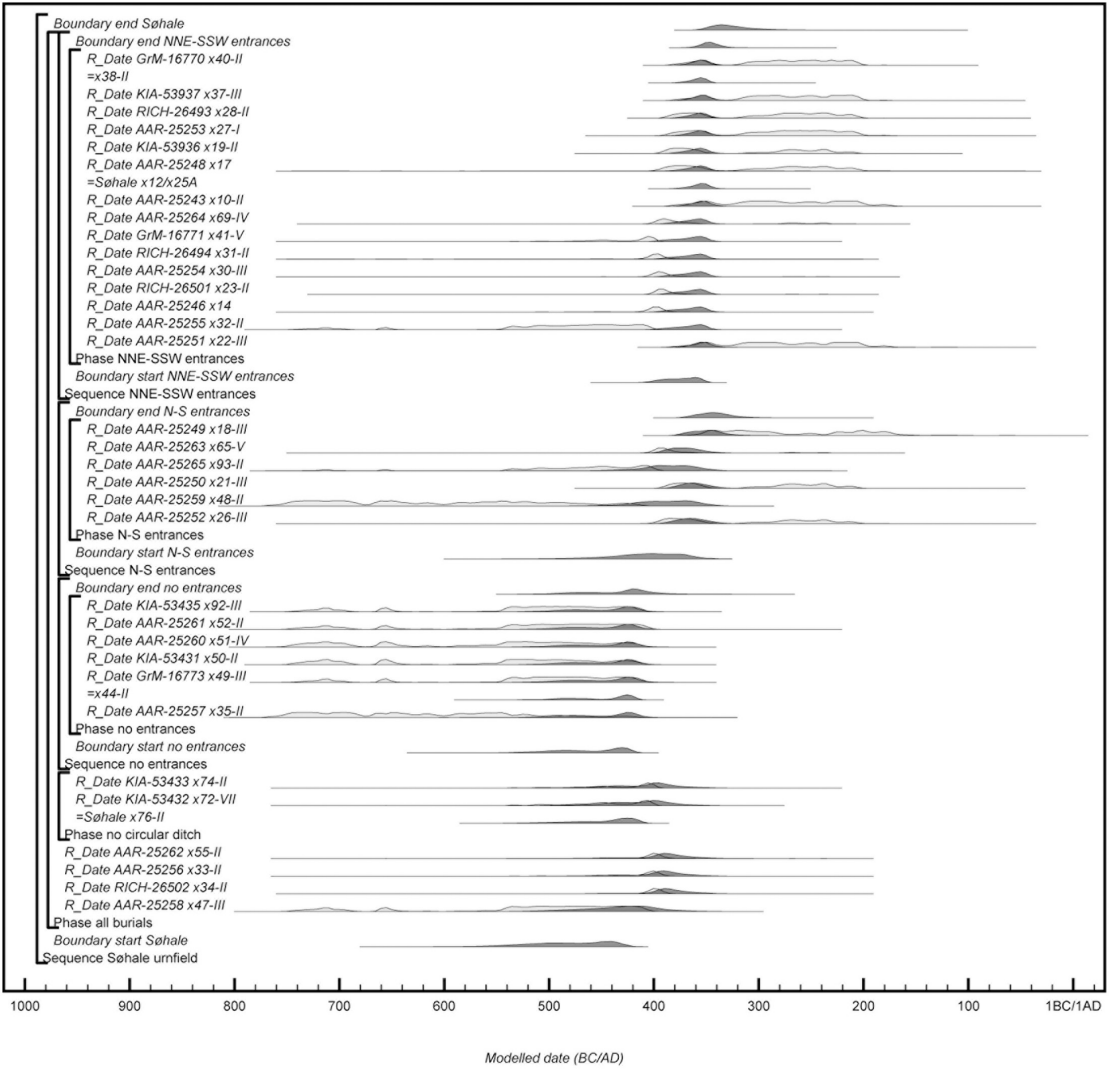
Chronological model of Søhale urnfield, based on the preferred urnfield model B.

**Fig 13:**
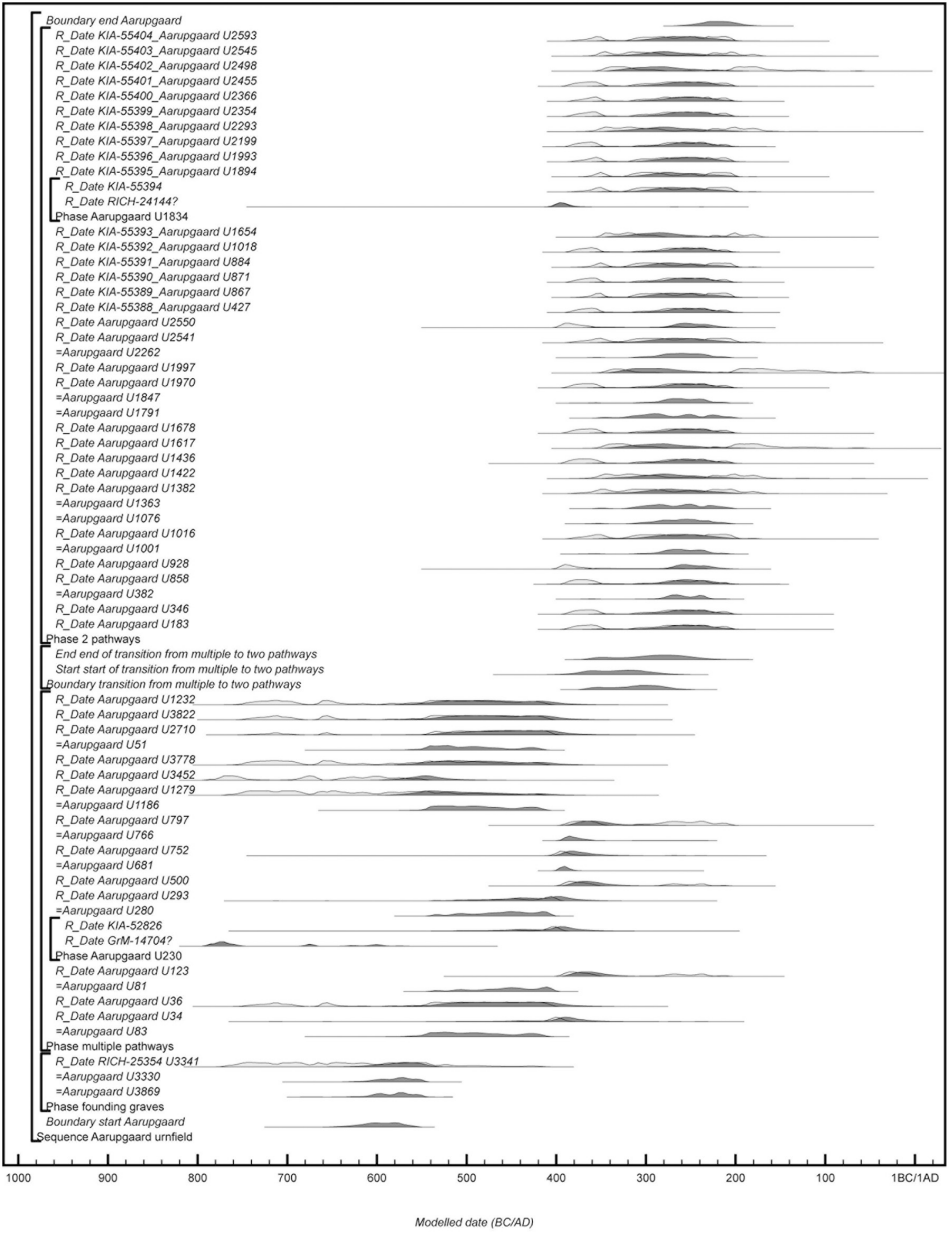
Chronological model of Aarupgaard urnfield, based on the preferred urnfield model B.

The **urnfield model B** is acceptable (A_overall_ = 67.6), and it estimates urnfield burial activity to have started *709–571 cal BC* (95.4% probability), probably *662–588 cal BC* (68.3% probability), and to have ended *260–125 cal BC* (95.4% probability), *probably 234–114 cal BC* (68.3% probability). The cremation OM *estimates 1-60yr offsets* (95.4% probability), *probably 1-24yr* (68.3% probability). Aarre urnfield is estimated to be in use for 158-264yr (68.3% probability), starting *617–457 cal BC* (95.4% probability), or *572–499 cal BC* (68.3% probability), and ending *356–214 cal BC* (95.4% probability), or 348–307 cal BC (68.3% probability) (Fig 11, S1.2.1 Fig in S1 File). The use period of Aarre urnfield is credible, although, given the limited number of samples, it cannot be excluded that the cemetery might have lasted longer. Søhale urnfield is estimated to have been in use for *92-186yr* (68.3%), starting *555–423 cal BC* (95.4% probability), or *510–432 cal BC* (68.3% probability), and ending *360–277 cal BC* (95.4% probability), or *351–314 cal BC* (68.3% probability) (Fig 12, S1.2.2 Fig in S1 File). The use period of Søhale urnfield is longer than estimated by urnfield model A, and now more consistent with the archaeological expectation. Aarupgaard urnfield is estimated to have been in use *344-404yr* (68.3% probability), starting *642–556 cal BC* (95.4% probability), or *616–571 cal BC* (68.3% probability), and ending *257–186 cal BC* (95.4% probability), or *238–201 cal BC* (68.3% probability) (Fig 13, S1.1.2.3 Fig in S1 File). The use period of Aarupgaard urnfield is longer than that of the other urnfields, but this is in agreement with the occurrence of both earlier and later artefact types at Aarupgaard than at Aarre and Søhale. The model is able to constrain the end boundary and provides unimodal solutions for nearly all burial dates. Urnfield model B is our preferred chronological model for modelling burial activity at Aarre, Aarupgaard and Søhale urnfields, because it evaluates more prior information.

#### Spatio-temporal development

**Fig 14:**
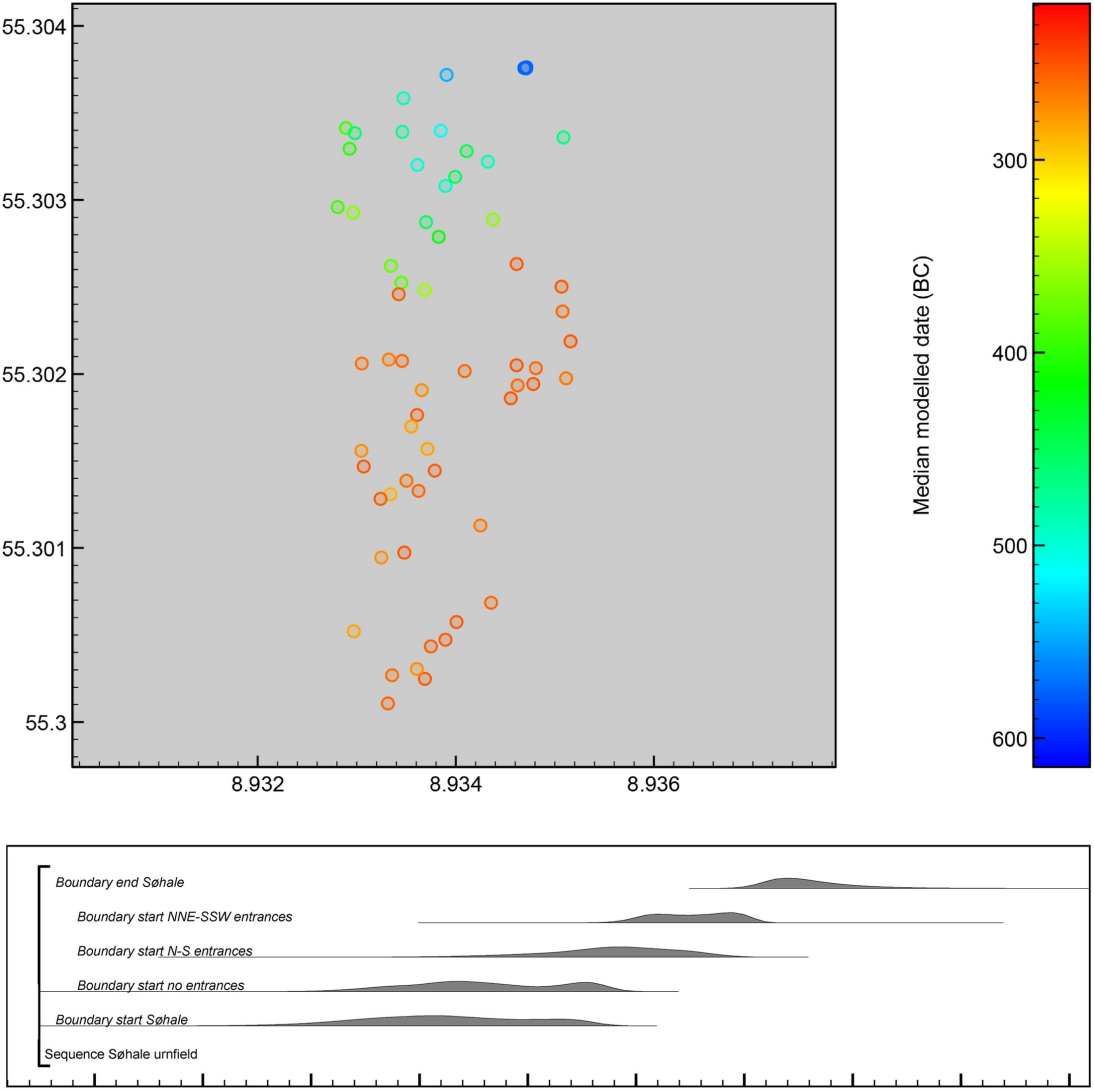
Alternative chronological models. Upper panel: Spatio-temporal plot of Aarupgaard urnfield based on horizontal stratigraphy (model C). Lower panel: Søhale urnfield, based on the orientation of the interruptions of the circular ditches.

An **alternative site model C** of Aarupgaard urnfield is based on earlier observations of horizontal stratigraphy [7, 88]. Burials are divided into horizontal groups of c. 200 individuals based on distance to the founding burials in the northern end of the urnfield (H1-H6) and are further divided into a main western burial group (M1), and smaller eastern burial group (M2). The starts of the M1 horizontal groups are constrained to follow the sequence: M1-H1, M1-H2, M1-H3, M1-H4, M1-H5, M1-H6. Model C makes no assumptions about the sequence of M1 horizontal group ends, in order to allow for later infilling of burials. There are fewer burials in burial group M2, making it necessary to merge horizontal groups 1–3, and there are no burials beyond horizontal group 4. The start and end of the M2 horizontal groups are constrained to follow the sequence: M2-1-3, M2-4. The other components of the model (i.e. dated burials at Aarre and Søhale) correspond to the preferred urnfield model B. The alternative model C is acceptable (A_overall_ = 83.7), confirming that site formation at Aarupgaard urnfield followed a north-southbound trajectory best described using horizontal stratigraphy (Fig 14 upper panel, S1.3.1–2 Figs in S1 File). The first 200 burials taking twice as long as the next 200 burials, indicating an increasing burial rate in the initial phase of the urnfield. Model C estimates burial activity to have continued slightly later when compared to urnfield model B, but it also estimates more fluctuating burial rates (S1.3.3 Fig in S1 File). Urnfield model B does not depend on these arbitrary divisions into horizontal groups, and it remains our preferred model.

#### Sensitivity testing

Sensitivity testing is a central part of Bayesian chronological modelling and it can be used to test alternative models using different prior information. To validate the preferred site model B, we test the influence of selected model constraints on posterior estimated output in an alternative site model D. We also test the reproducibility of the posterior estimated start and end of burial activity, because it coincides with a major plateau in the ^14^C calibration curve c.750-400 cal BC, and an inversion c.320-200 cal BC.

In the preferred urnfield model B for Søhale, burials are grouped based on the orientation of interruptions of the circular ditches, but the three groups are not placed in any sequence. We test the chronological sensitivity of these groups in an **alternative site model D** by constraining the start of entrance groups to follow the sequence: no interruptions, N-S, NNE-SSW. The remaining part of model D equals urnfield model B. Model D is acceptable (A_overall_ = 84.6), permitting the possibility that the orientation of interruptions of circular ditches is time dependent, following the order as proposed by Møller et al. based on ^14^C ages (Fig 14 lower panel) [15]. Constraining the order of introduction has however, no influence on the posterior estimated output and the alternative model is therefore rejected in favour of the less constrained urnfield model B.

**Fig 15:**
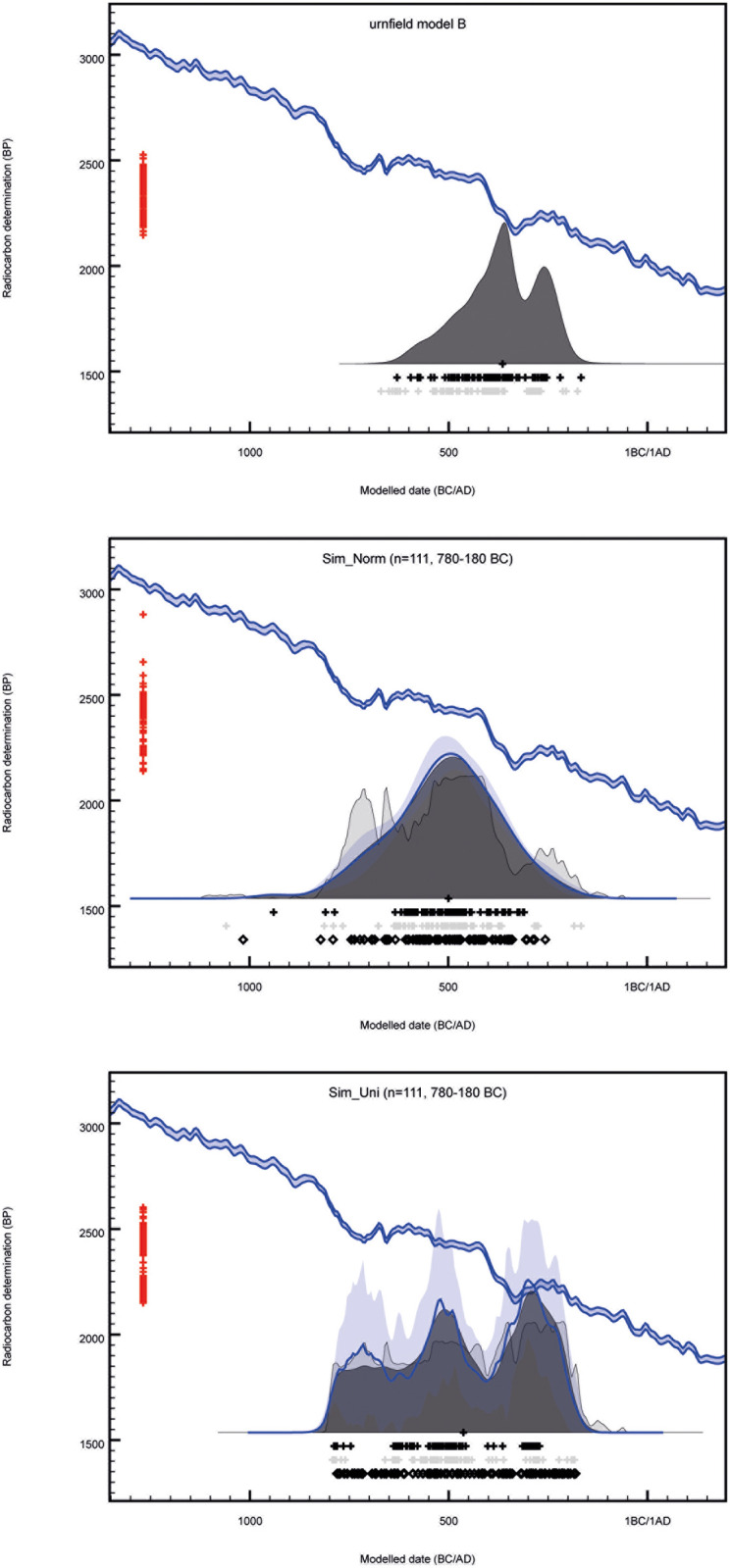
Kernel density estimates of actual and simulated burial activity. Upper panel: KDE plot summarizing urnfield burial activity as estimated by the preferred urnfield model B [119]. Simulating burial activity of 111 simulated ^14^C dates between 780–180 BC using the OxCal function KDE_Model with defaults parameter values [119]. Middle panel: simulated ^14^C dates drawn from a normal distribution (Sim_Norm). Lower panel: simulated ^14^C dates drawn from a uniform distribution (Sim_Uni). Red crosses (left) show median uncalibrated ^14^C ages, black crosses (below) show median modelled calibrated dates, grey crosses (below) show median of simple calibrated dates and diamonds (below) show known calendar ages. The relevant section of the IntCal20 calibration curve is shown for references [64].

Bronk Ramsey [119] has shown that realistic density distributions of uniformly distributed dates can be estimated using the default KDE_Model provided in OxCal. Kernel density estimates (KDE) can also be used to visualize burial activity and changes in burial rates and a KDE plot of all posterior estimated burials from preferred urnfield model B has two peaks around the time of an inversion of the ^14^C calibration curve c. 320–200 cal BC (Fig 15 upper panel). KDE plots of the individual urnfields all have a single peak towards the end of their use-life, but at different points in time (S1.2.1–3 Figs in S1 File), which combined produce the two peaks. We test if the observed peaks in burial activity are mere artefacts of the ^14^C calibration curve by creating two synthetic datasets, each with 111 simulated ^14^C dates between 780–180 BC, drawn from a normal distribution (Sim_Norm) and a uniform distribution (Sim_Uni), respectively and summarized using the KDE_Model function (normal distribution: Fig 15 middle panel, uniform distribution: Fig 15 lower panel) [119]. The KDE of the synthetic normally distributed dataset depicts the expected bell curve but bears little resemblance to the KDE of the urnfield burials. The KDE of the synthetic uniformly distributed dataset is not able to retrieve the known uniform distribution but gives a false trough in the 5th century and a false peak at c.300 BC, which are not seen in the real data, while the 4th and 3rd century peaks in the real data are not visible in the simulation and can therefore be considered as reliable [120].

We test the influence of the ^14^C calibration inversion c.320-200 cal BC on the precision of the posterior estimated output by adding synthetic dates with +-25yr uncertainties to the real Aarupgaard dataset, using the OxCal function C_Simulate. These simulated dates are not affected by the shape of the ^14^C calibration curve. Adding simulated calendar dates between 250–200 does not shift the end boundaries compared to those obtained under preferred urnfield model B, probably due to the uniform distribution favoured by the model. This implies that the latest real burials must date at least to around 200 BC, but not if they might be even later. We explore this by adding simulated calendar dates between 200–150 BC, but even though this shifts the estimated end boundary later, it still estimates the youngest simulated burials to be too early (S1.4.1 Fig in S1 File). By repeated modelling of younger dates with slightly varying calendar dates and sampling intensity (decadal or sub-decadal) it becomes clear that adding only a few dates between 200–150 BC produces posterior estimates around 200 BC, whereas adding at least a handful of dates later than 200 BC will produce a slightly later end boundary. To recover the true calendar dates the youngest simulated dates must be later than c.150 BC, but also have a certain sampling density.

Burial U1617 from Aarupgaard has the youngest ^14^C age BP (2163 ± 28) of the entire dataset. Calibration with IntCal20 gives a slightly later calibrated date in comparison to IntCal13, but both produce bimodal solutions with close to equal posterior probabilities on either side of the ^14^C inversion (IntCal20: 350–305 cal BC at 31.6% probability, 208–156 at 36.7% probability). The archaeological prior information does not allow us to reject either possibility and because the urnfields were abandoned around this time, it would be difficult to sample even later burials. Our preferred urnfield model B estimates the burial date of U1617 to *322–257 cal BC* (68.3% probability), i.e. centred on the early-middle part of the ^14^C inversion.

Based on this, we conclude that the latest real burials probably do date to around 200 BC, as also estimated by the preferred urnfield model B. We cannot reject the possibility that a few burials date to the first half of the second century BC, but we would then expect different material culture (e.g. fibulae and knives) to be present at Aarupgaard, and we therefore find this to be unlikely.

### Modelling artefact currencies

**Fig 16:**
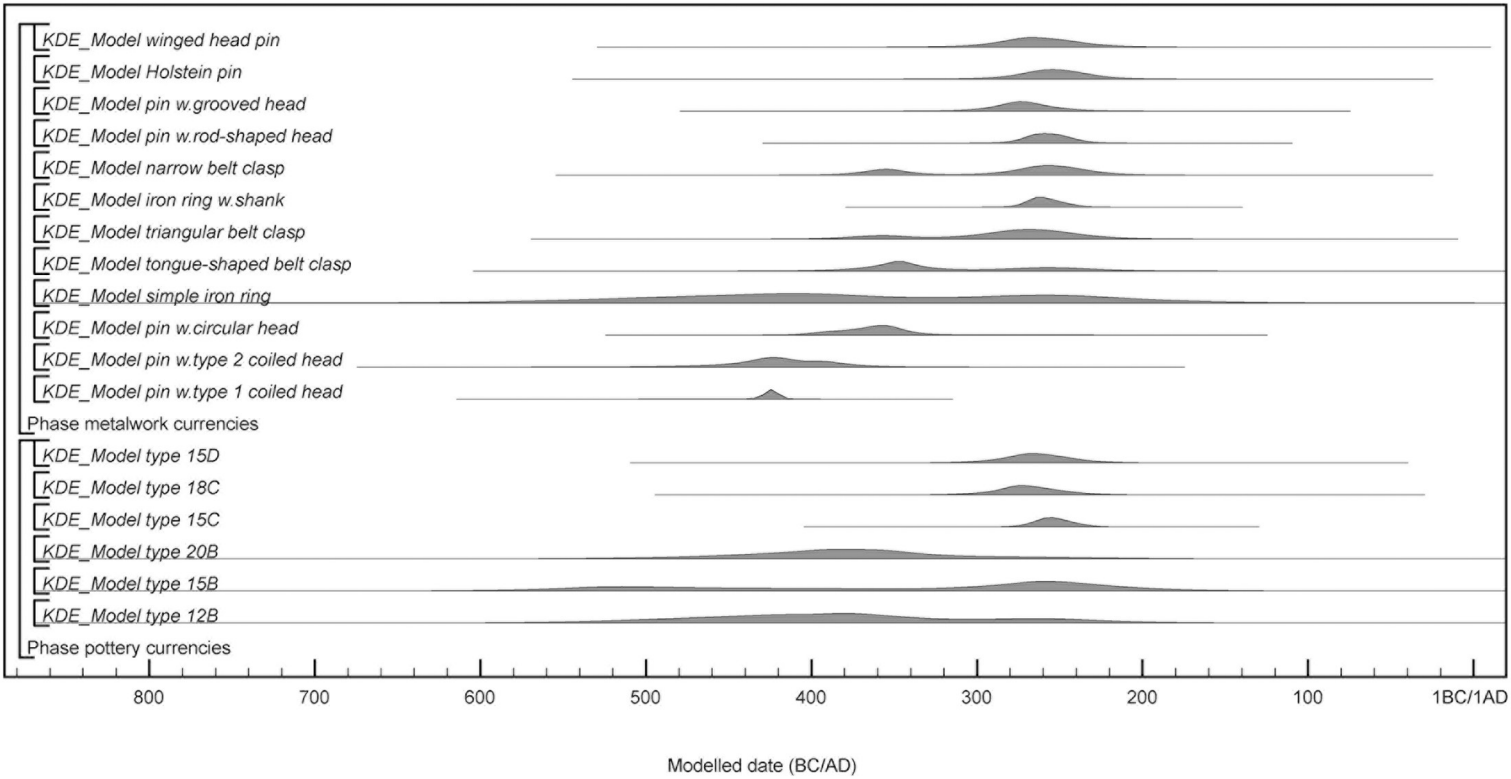
Artefact currencies summarized using the default KDE_Model function in OxCal.

The artefact currencies are modelled in a separate step. The posterior estimated burial dates from urnfield model B are saved (using the OxCal function Prior) and the currencies of types with minimum four dated cases are summarized using the default KDE_Model function in OxCal (Fig 16) [119]. Though providing a first overview of the currency distributions, this is inconsistent with the archaeological expectations because, aside from the simple iron ring, there is no metalwork dating to the early 5^th^ century BC. Overall, these KDE models also estimated that the currencies ended earlier than expected.

The posterior estimated burial dates (OxCal Priors) are instead modelled as dates in two separate bounded phases of pottery and metalwork currencies, which are constrained to start after the end boundary of period VI, similar to urnfield model B. Currencies dated by 1–3 cases are modelled in unbounded phases, currencies dated by 4–9 cases in uniform bounded phases, and currencies dated by ≥10 cases are modelled using trapezium boundaries [64, 66]. We test and find that posterior estimates on pottery are unaffected by constraining their order of introduction as defined by correspondence analyses by Jensen [10] (starting order: 15B, 12B, 20B, (15C & 18C)). It is therefore preferable not to include prior information on pottery sequences. It is however not possible to model the metalwork currencies without constraining their order of introduction (pin sequence: pin with type 1 coiled head, pin with type 2 coiled head, pin with circular head, (pin with grooved head and pin with rod-shaped head), (Holstein pin and winged head pin); sequence of belt equipment: simple iron ring, (tongue-shaped belt clasp and triangular belt clasp), (iron ring with shank and narrow belt clasp)). We note that artefact currencies are limited by the use-life of the urnfields, and some types might well have been in use longer elsewhere.

To identify possible heirlooms with residence-time offsets, we apply OxCal’s default General Outlier_Model to the currency model, assigning a prior probability of 5% that each burial date is not applicable to the currencies of the associated pottery and metalwork [116]. The posterior probabilities of being an outlier are higher than 5% for 24 artefacts from 22 graves, of which 13 artefacts from 12 graves have posterior outlier probabilities >10% (S1.5.1 Table and S1.5.1 Fig in S1 File). Most of these cannot have been heirlooms, because the currency model favours a production date later than the burial date. Four outliers are caused by the model being unable to distinguish between the early and late legs of the ^14^C calibration inversion c. 320–200 cal BC. Seven artefacts with outlier probabilities >10% from as many graves are interpreted as heirlooms, however, because their currency-model dates are earlier than the respective burial dates. Heirlooms can also be identified when graves contain artefacts of multiple types, such as U1617 from Aarupgaard, where a circular pin is estimated by the currency model to be earlier than the burial date, whereas the modelled dates of the remaining artefacts are consistent with each other and with the burial date, clearly demonstrating that the pin must be an heirloom with a considerable residence time. We subsequently remove the General OM and instead apply individual residence-time offsets of 50 ± 25yr to the seven identified heirlooms (bold red font in S1.5.1 Table in S1 File). The currency model is acceptable (A_overall_ = 104.3), and all heirlooms have acceptable indices of agreement with a mean residence offset of 67yr.

#### Pottery currencies

**Fig 17:**
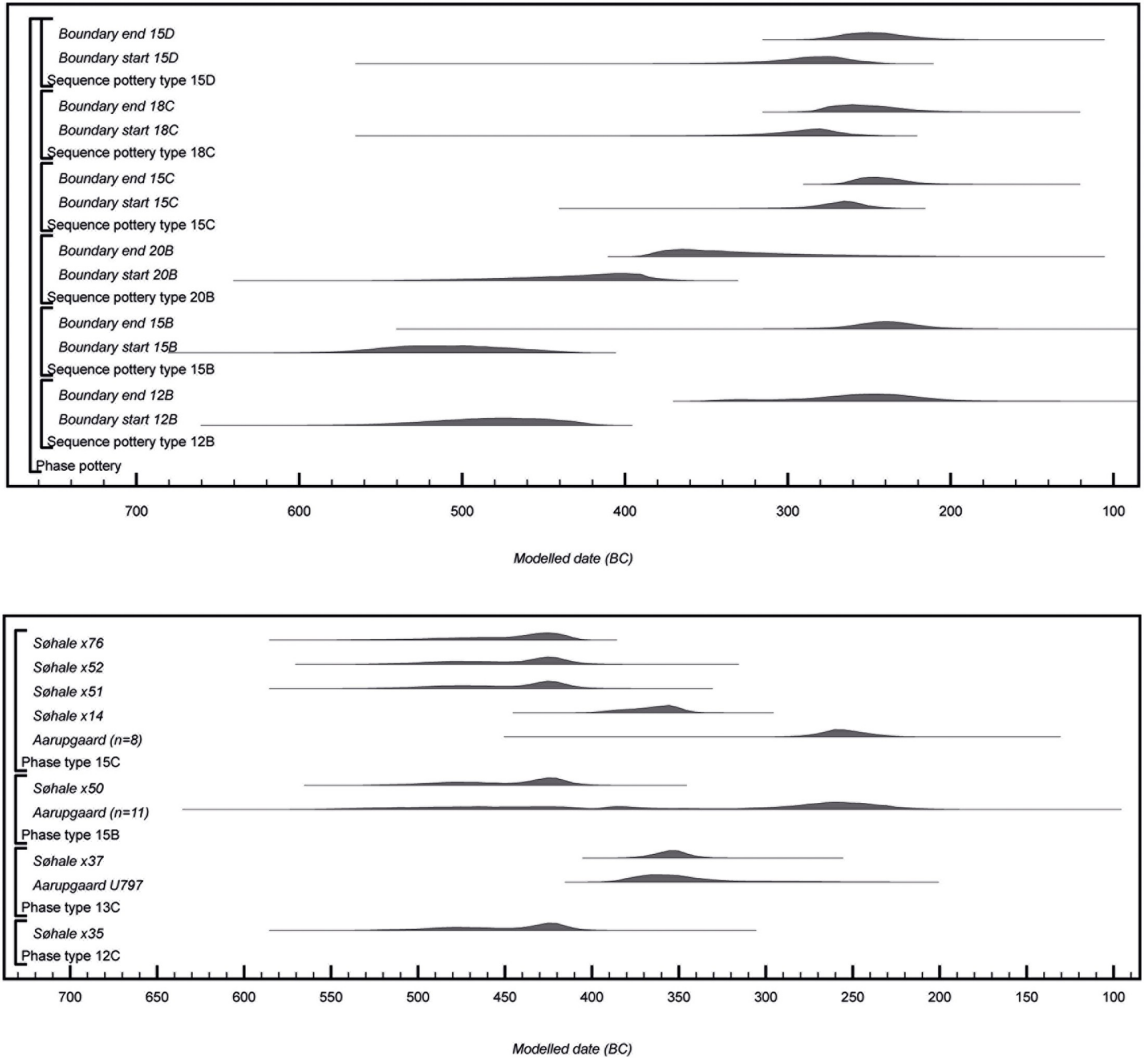
Posterior estimated dates on pottery from Aarupgaard and Søhale urnfields. Upper panel: posterior estimated start and end of pottery currencies from Aarupgaard with minimum four dated specimens per type. Lower panel: comparing pottery currencies from Aarupgaard to posterior estimated burial dates from graves from Søhale containing pottery.

In total, 14 types of pottery from Aarupgaard urnfield are modelled: five of these in bounded phases and one using trapezoidal phase boundaries (Fig 17 upper panel, S1.5.2 Fig in S1 File). The modelled currencies have a mean duration of 62yr ranging from 33y to 313yr (68.3% probability). Pottery from Søhale urnfield is not included in the modelling, but results from the two urnfields are compared below. Individual currency models are provided in S1.5.3-S1.5.8 Figs in S1 File. The currencies evidently have large overlaps, but their order of introduction is estimated to follow the sequence: 15B, 12B, 20B, 18C, (15C & 15D), in agreement with the order given by Jensen [10]. He dated type 20B to the Late PRIA, however, but the ^14^C evidence evidently supports an earlier introduction.

Seven burials from Søhale urnfield contained four types of diagnostic pottery and we compare the posterior estimated burial dates of these to the estimated currencies based on burials from Aarupgaard (Fig 17 lower panel). Burial x50 contained a type 15B vessel and its date is comparable to the earlier instances of this type at Aarupgaard, four burials contained type 15C vessels that all date significantly earlier than at Aarupgaard, but burial x37 that contained a type 13C vessel is contemporary with another similar type vessel from Aarupgaard. Burial x35 contained the only type 12C in the dataset. It might be suggested that pottery types appeared at Søhale earlier than at Aarupgaard, but the present dataset is too small to reveal different temporal patterns between the urnfields.

#### Metalwork currencies

**Fig 18:**
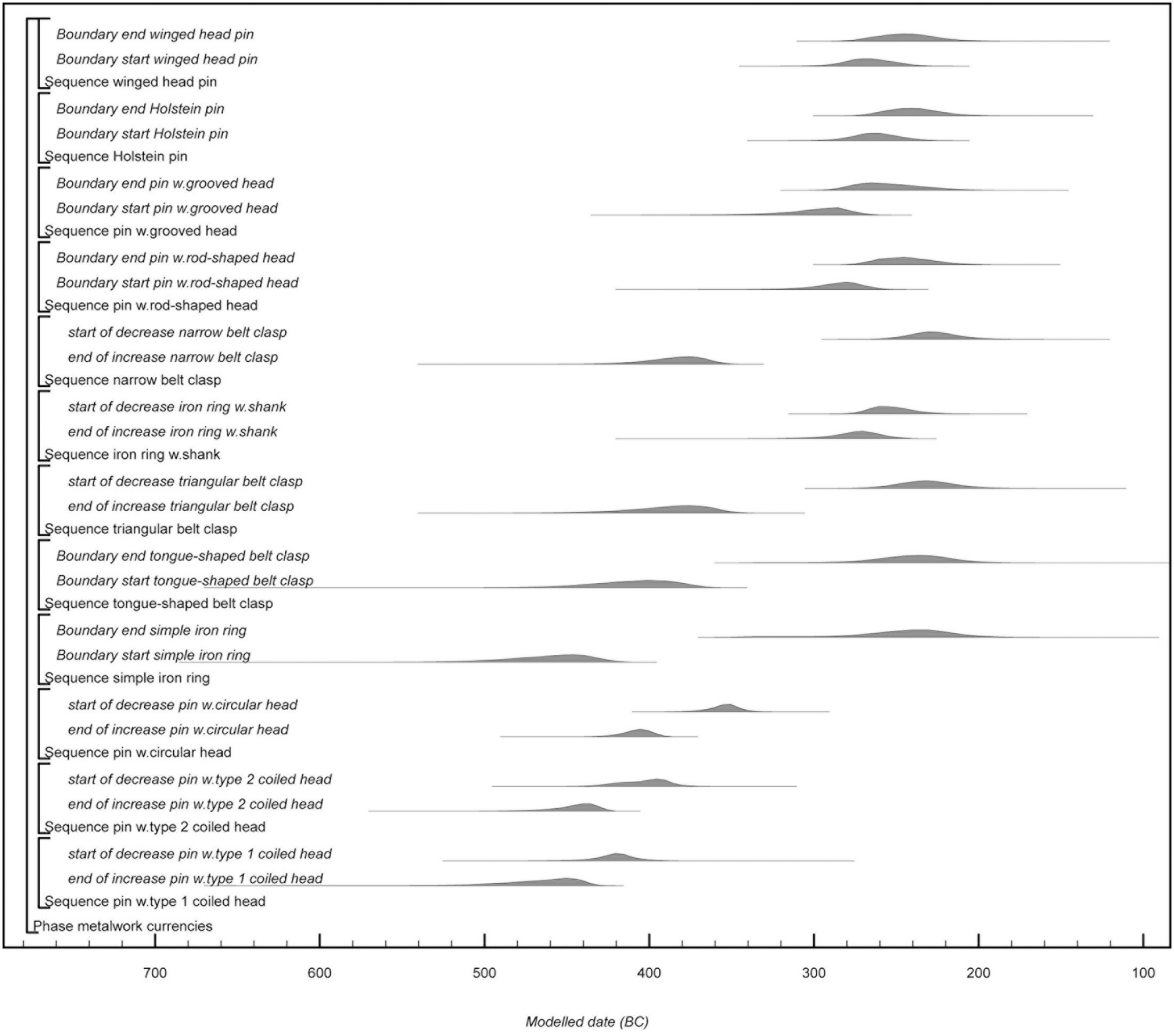
Posterior estimated metalwork currencies with minimum four dated specimens per type.

In total 15 types of metalwork are modelled, and 12 of these are modelled in bounded phases with predefined order of introduction (Fig 18, S1.5.9 Fig in S1 File). Individual currency models are provided in S1.5.10-S1.5.21 Figs in S1 File. The modelled currencies have a mean duration of 48yr ranging from 30y to 217yr (68.3% probability). The currency model estimates a high degree of overlap between types. Bomb head pin, eyelet ring and bronze neck ring are only present in a few burials, but are estimated to date to the 5^th^ century, first half of the 4^th^ century and to the 3^rd^ century, respectively. Simple iron ring was probably a generic type used throughout the PRIA. Earlier, we rejected the typological differentiation between small and large pins with circular heads. This is further supported by the ^14^C evidence that fails to show a dependency between mean ages estimated by the currency model and head size index (S1.5.22 Fig in S1 File).

## Discussion

Chronology construction, or situating archaeological material within space and time, is a time-honoured tradition in archaeology. It is also a prerequisite for most archaeological studies and in turn influences our resulting narrative of the past, e.g. if two key phenomena are overlapping or if one leads to the other? A good chronological resolution enables investigations not only into the material record and burial practices, but also allows us to address overarching questions regarding e.g. social structures or religious and cultic practices. The range of individual chronologies is decided by the presence of shared material culture, but can be extended or correlated by cross dating imports. In the absence of imports chronological frameworks can instead be synchronized using absolute dating. The same approach can be used to correlate different find materials, such as the many archaeological chronologies that are traditionally based on metal-based typologies, whereas ceramic types are only subsequently attributed. This is also the case for PRIA in Denmark where there are demonstrated difficulties correlating typo-chronologies of metalwork and pottery [7, 8]. Here metal artefacts are practically absent from settlement contexts and without a detailed chronology for pottery, it is difficult to compare settlement and funerary evidence.

Chronological frameworks assume change happened continuously and consequently divide the past into non-overlapping units of time, but the application of absolute dating now challenges the validity of this notion. Instead, prehistory is made up of long-term transformations, i.e. from bronze to iron technology, but any of these transformations entail a multitude of smaller-scale changes. Whether the smaller-scale changes are visible in the archaeological record depends on the resolution of the available data, but also on adopting an appropriate research approach that permits non-uniform output.

### Insight into a dynamic material culture

The main new contribution of the study is the modelling approach that provides a detailed insight into the temporal dynamics of artefact currencies without *a priori* imposing the rigid boundaries of a chronological framework. Artefact typology is often used as prior information in Bayesian chronological models aiming to establish site chronologies and absolute chronological frameworks [e.g. 121], but typologies are rarely the intended focus of such investigations. Important exceptions are the seminal study on British Bronze Age Metalwork [122], the ‘Dating Celtic Art’ project [123], and the ‘Anglo-Saxon Graves and Grave Goods’ project [98]. Common for these are that currencies are assumed to have a uniform distribution, i.e. dated artefacts are equally likely to date to any point in between a rapid introduction and a later equally rapid abandonment. It might rightly be questioned how realistic such a scenario is and it is now possible to model artefact distributions in OxCal using the trapezium prior model, which is well suited for modelling non-instantaneous cultural changes, particular where phases are expected to be overlapping and transitional periods to have a duration [64, 66]. A trapezium model divides a distribution into three parts, first an introductory period; followed by a period of maximum use; before a period of decline [27, 63]. Seriation of artefacts from PRIA show individual currencies to have significant increase and decrease ‘tails’ and a large degree of overlap between types [10], leading us to model seven currencies using trapezoidal boundaries. We decided to focus on types with min. 10 dated cases because the model would otherwise introduce large uncertainties compared to a bounded phase model. This only includes a single pottery type, but the dynamics of type 15B appears to be comparable to the metalwork types. The periods of introduction and abandonment are estimated to last a couple of centuries, respectively, although a bimodal distribution for narrow belt clasp cannot be rejected, which leads to a longer estimated introduction period (S1.6.1a Fig in S1 File). We find that letting the introduction and abandonment of the currencies have a duration, rather than being instantaneous, is a better fit with the actual artefact frequencies observed by Jensen [10].

**Fig 19:**
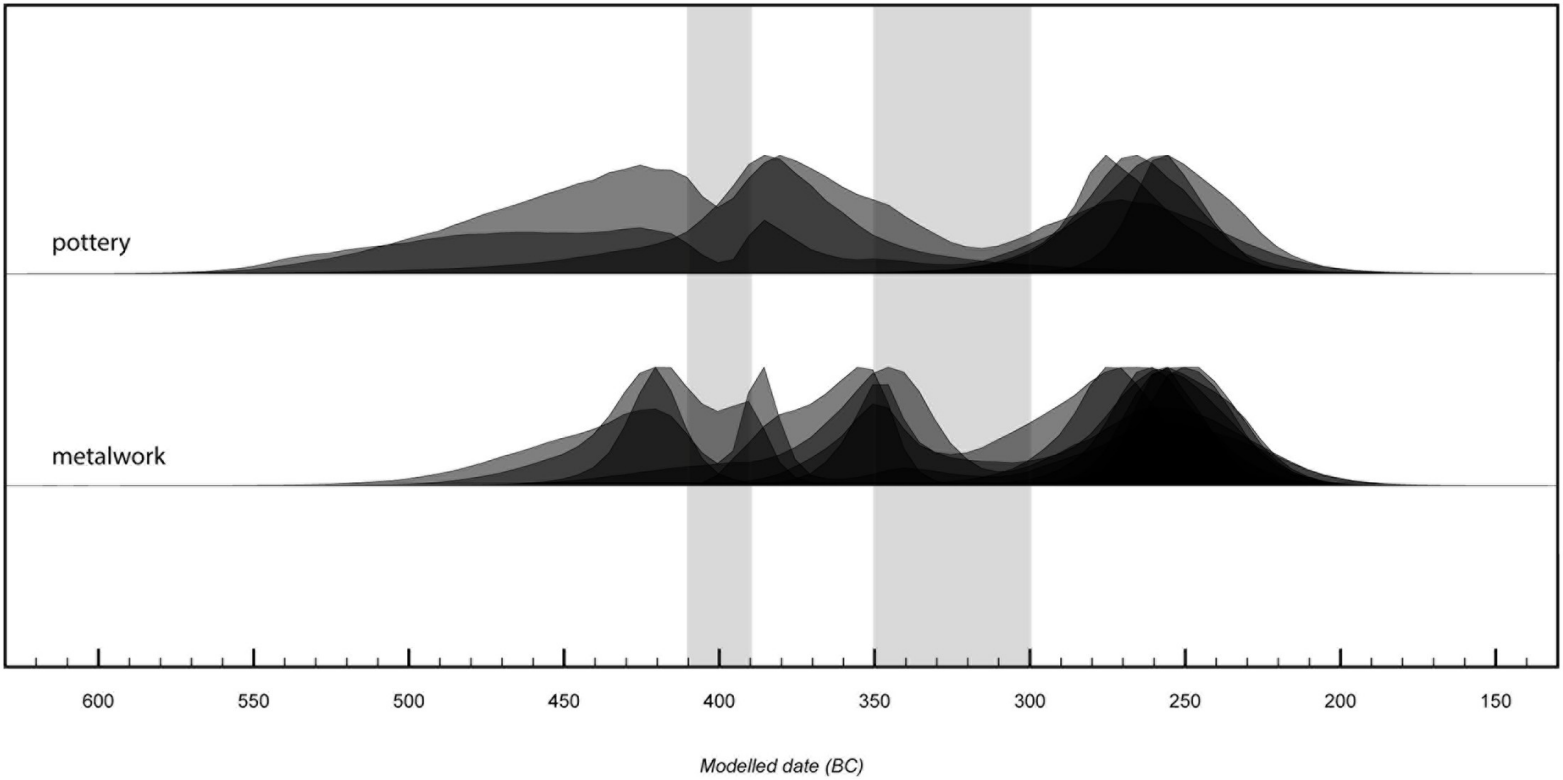
Currencies of pottery and metalwork are stacked, respectively, and periods with higher rate of change in material culture is marked with yellow bars.

The currency models estimate very variable currency durations (pottery 33-313yr, metalwork 30-217yr (68.3% probability); S1.6.1b Fig in S1 File). Pottery types 12B and 15B were in use significantly longer than the other types, probably throughout the EPRIA, which is contextually supported by associated artefacts spanning most of the typological sequence of metalwork. The remaining three pottery types were probably in use for 2–4 generations (mean = 34yr). It has been suggested that vessel types used in the public domain are more likely to change fast over time, whereas other types appear to have remained largely unchanged for longer periods [124]. The large storage vessels often reused as funerary urns probably belong in the latter category, but it is possible that currencies of smaller vessels not included in this study are more dynamic. The early pin types (pin with type 1 and type 2 coiled head and pin with circular head) were likely in use for 2–3 generations (mean = 50yr), but it is possible that several later metalwork types (iron ring w. shank, pin w. rod-shaped head, pin w. grooved head, Holstein pin and winged head pin) were in use for only 1–2 generations (mean = 19yr). The relatively long estimated durations of tongue-shaped, triangular and narrow belt clasps are at least partly caused by them coinciding with the ^14^C calibration inversion c.320-200 cal BC where even relatively precise ^14^C ages can produce calibrated date ranges spanning the whole inversion period Modelling currencies dynamically has the great advance that it becomes possible to identify periods with lower and higher rates of change during the PRIA (Fig 19). Both pottery and metalwork have considerable overlap of consecutive currencies and when combining these a pattern with peaks and valleys emerges. The decades around the start of the 4^th^ century BC (c.410-390 BC) have a high rate of change with more types going out of use while new types are being introduced. The remaining first half of the 4^th^ century BC (c.350-300 BC) has a low rate of change as these types continue to be in use, before the rate increases in the second half of the century when types are again abandoned while new types are being introduced. Followed by a period with lower rate of change in the first half of the third century BC. The start and end of the investigated period are probably also periods of rapid change, but it is difficult to demonstrate, as these are not constrained by earlier or later occurring currencies.

Modelling periods of introduction, main use and decline offers a new and dynamic perspective on artefact currencies. The individual artefact types do not themselves introduce large changes to the society, but by documenting small-scale changes and demonstrating how these are concentrated in periods with a higher rate of change, it becomes clear how the redefinition of material culture is actively used in an ongoing negotiation of a dynamic society. The majority of types are introduced rather rapidly and are not in circulation long after the production ends, which demonstrates a readiness to adopt new designs of existing object forms. Particularly the metalwork types were in use for shorter periods, whereas the pottery can be divided into shorter lasting types and generic types that were in use throughout the period. Typological analyses of comparable EPRIA cemeteries from Schleswig-Holstein have demonstrated pottery to have a limited chronological sensitivity compared to metalwork [9]. The Danish material share the same tendency, although it is less distinct. The difference between the Danish and German material might be caused by comparing absolute and relative dated currencies, but there are so far no large ^14^C dataset available on German material.

### Heirlooms and residence time

The production date and deposition date of an artefact are separate events that can be greatly removed from each other, i.e. the artefact has a residence time. If older individuals are buried with artefacts that they had received at a young age those artefacts will have a considerable residence time, which has led Trachsel [30] to caution against assigning currency durations shorter than a human lifetime. There is no available information on the life expectancy in the PRIA or at what age individuals would receive dress accessories such as pins and belt claps. If the artefacts were instead passed down as heirlooms, the residence time would increase significantly. Another important factor when discussing residence time of artefacts is durability of the material in question. Metalwork has a high durability is therefore considered likely to have a residence time. Pottery is more fragile and less likely to accumulate significant residence times. All urns from Søhale urnfield have considerable wear of the bottom and they must have been used for some time prior to being re-used as funerary urns. No experimental studies have been conducted on the use wear of PRIA pottery, but it is plausible that the bottom of a vessel would show signs of use after even a few years. It is demonstrated that some of the pottery currencies have been produced over long periods, but this does not contradict individual vessels having a short or even negligible residence time. There is a however a risk of metalwork having a residence time.

**Fig 20:**
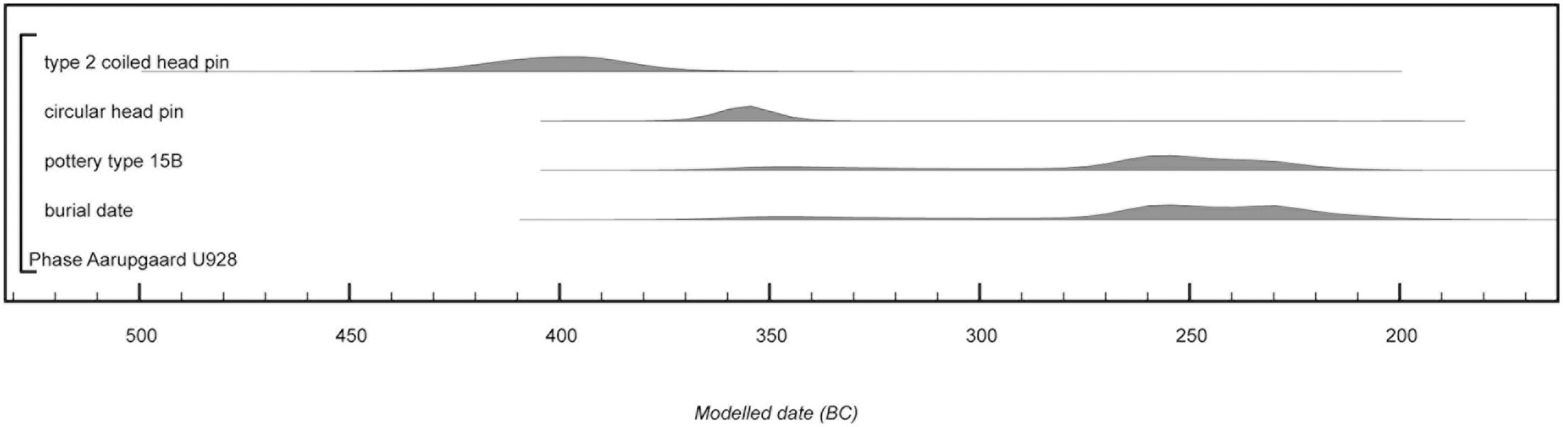
Burial U928 from Aarupgaard urnfield. The significant residence time of the pin with type 2 coiled head in relation to the burial date makes it a likely heirloom.

We estimate the residence times of identified heirlooms (objects initially detected as improbably recent outliers in currency models) by calculating the difference between the posterior estimate of the burial date and the posterior estimate of the production date of the earliest dating artefact in the grave. We assumed that the residence offset will usually be less than century (normal distribution 50 ± 25yr). We find residence offsets up to 102y*r* (68.3% probability), with a mean of 62yr (S1.6.2 Fig in S1 File). There is no available information on the age of the individuals buried with heirlooms, and it cannot be ruled out that some of them were older individuals buried with an object they had acquired early in life, similar to Trachsel’s suggestions regarding a 40+ aged individual from the central chamber of the ‘Magdalenenberg’ [30]. The difference between the burial date of Aarupgaard U928 and the production date of a pin with a type 2 coiled head and is however larger than a single individual’s lifetime (Fig 20). The burial date and a circular head pin from the same grave are estimated c.350 and c.250 BC, respectively, which correspond to before and after the ^14^C calibration inversion. Based upon the grave placement in the urnfield (horizontal group M1-H3), the earlier solution is probably more likely, which would incidentally make the pin with type 2 coiled head less of an outlier in the burial context.

### Absolute chronology

The archaeological literature contains a vast number of chronological frameworks for all time periods and regions across the globe and correlating any of these is a major task only achievable through international collaborations [e.g. 3], and in many cases not possible without using absolute dating, such as ^14^C dating [53, 121], or wiggle-matching dendrochronologies [125]. The start of the Iron Age in Southern Scandinavia is believed to coincide Ha D in Central Europe, but the argument therefore rests on a very limited number of imports. Instead, the existing relative chronological framework is determined on typo-chronologies, largely unconstrained by absolute dating (although see [15]). Recent studies have however demonstrated that the Iron Age chronology for Central Europe and possibly also Southern Scandinavia, might be in need of revisions [53, 125]. In the following, we provide an absolute chronological framework of the Early Iron Age in Denmark based on posterior estimated probabilities saved as priors from the urnfield model B and the currency model.

**Fig 21:**
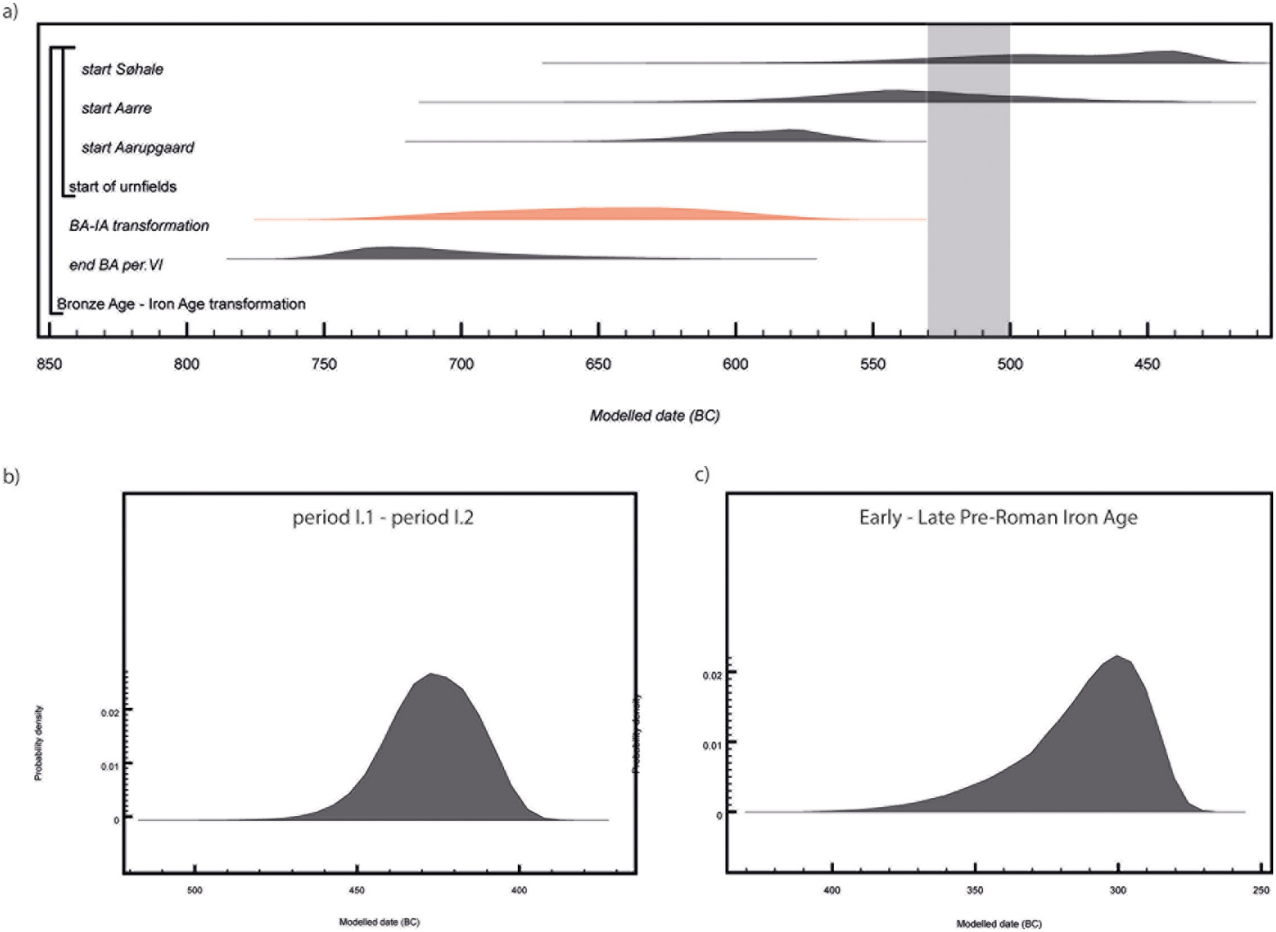
Chronological transitions of the Pre-Roman Iron Age, as defined by the typo-chronology of Jensen [10]. a) chronological model of the Bronze-Iron Age transformation, the red distribution is the estimated transitional period between the end of Bronze Age per. VI (690–604 cal BC at 68.2% probability) and the start of burial activity at Aarupgaard, Aarre and Søhale urnfields, the grey area marks the conventional transformation, b) per. I.1-I.2 (438–410 cal BC at 68.2% probability), c) Early-Late Pre-Roman Iron Age (325–286 cal BC at 68.2% probability).

The transformation from Bronze Age to Iron Age is usually assumed to occur around 500 BC [10, 126], or possibly in the later 6^th^ century BC [1, 15, 127]. It has been suggested that the transformation coincides with the Ha D1-D2 transition in Central Europe [13, 127], which following Rieckhoff and Biel occurs 550 BC [128]. In northern Germany the start of the Pre-Roman Iron Age is relatively dated to the late 6^th^ century BC [3, 78, 129, 130]. The transformation is not defined by the introduction of iron technology alone, but by a combination of small-scale changes in material culture, introduction of urnfields as a new funerary tradition and society structure changing from being kinship based towards more family and village orientated. Settlement structures and economy are often described as being stable before and after the transition [127, 131], although it coincides with a reorganisation of the cultural landscape and new ways of claiming ownership of the land [76].Following these observations, we model a transition period following the end of Bronze Age period VI and before burial activity at Aarupgaard, Aarre and Søhale urnfields started (Fig 21a). The model shows the transition occurred in the 7^th^ century BC (*690–604 cal BC* at 68.2% probability), c.50-150yr earlier than usually assumed and earlier than the current date of the Ha D1-D2 transition in Central Europe. In another study [53], we found that the Ha C-D transition in Southern Germany took places some decades before the traditional 620 BC date [132], which incidentally extends per. D1 and possibly shifts the D1-D2 transition earlier and in the direction of the estimated Bronze-Iron Age transformation in Southern Scandinavia. This leaves little time for Bronze Age per. VI, when a new spectrum of artefact types was introduced, clearly separating it from the previous period V [130, 133]. In opposition, to the continuation of artefact types across the Bronze-Iron Age transformation [10]. The earlier transition date contributes to an ongoing debate, whether per. VI should instead be understood as a transitional phase between the Bronze and Iron Ages, or even if it might be incorporated into the Iron Age [10, 127, 130, 133, 134]. Another possibility is overlapping phases with different geographical distributions, comparable to what has been suggested for the Hallstatt-La Téne transition in Central Europe [29], but this will need further investigation.

The typo-chronological system of Jensen [10] defines the transition between Early PRIA periods I.1 and I.2 as occurring after the introduction of pins with type 1 and type 2 coiled head and pottery type 12B, but before the introduction of pin with circular head and pottery type 18C. A chronological model following this construction shows the transition to occur in the late half of the 5^th^ century (*438–410 cal BC* at 68.2% probability, Fig 21b). This is a few decades earlier than the previously suggested transition c.400 BC [15], but it is not contradicted by the archaeological information.

The transformation from Early to Late PRIA coincides with a clear break in material culture [10], and the abandonment of settlements and cemeteries across most of western Denmark [135]. It is usually assumed to occur around 250–200 BC [10, 89], although Becker [1] has suggested it to occur around 300 BC and Møller et al. [15] have suggested the early-mid 3^rd^ century BC. The typo-chronological system of Jensen [10] defines the transformation as occurring after the introduction of pin with circular head, triangular belt clasp and pottery type 15C, and coinciding with the introduction of iron ring with shank, pin with winged head, pin with grooved head and Holstein pin. A chronological model following this construction shows that the transformation from Early to Late PRI probably occurred in the late 4^th^-early 3^rd^ century BC (*325–286 cal BC* at 68.2% probability, Fig 21c). This is considerably earlier than usually assumed, but in agreement with suggestions by Becker and Møller et al. (cf. Fig 2), and the date is additionally supported by the transformation coinciding with the abandonments of Aarre and Søhale urnfields in the late half of the 4^th^ century BC (cf. Figs 11 and 12).

This study demonstrates the need to adjust relative chronological frameworks using absolute dating, including the chronological framework of the Late Bronze Age and Early Iron Age in Southern Scandinavia. The introduction of the urnfield phenomenon in Denmark is a cultural marker of the start of the Iron Age and we estimate this to occur already in the 7^th^ century BC. There is little absolute dating evidence from urnfields in Schleswig-Holstein (although see [77]), but comparable material from the Netherlands is dated to Late Bronze Age-Early Iron Age, where the latter start c.800 BC [136]. Urnfields in Belgium are primarily a Bronze Age phenomenon, although ^14^C dating has also here challenged the traditional chronological framework [e.g. 137]. We estimate the Danish urnfields were in use for *376-537yr* (68% probability), with Aarupgaard urnfield as the only sites that continued to be in use in the first part of the Late PRIA. There is no available absolute evidence to contradict shifting the Early PRIA chronology by c.150yr, with per.I.1 lasting above two centuries, and per. I.2 lasting above one century. Part of the shift might however be explained by considerable temporal differences in the adoption of cultural development and further investigations of the absolute chronological framework need to pay particular attention to other regions in Denmark and northern Germany.

## Conclusions

Since the Danish Pre-Roman Iron Age was defined as an archaeological period in the 1890s, it has largely relied on relative typo-chronological analyses of artefact assemblages from urnfields. This study continues a long research history, but also presents the first large-scale ^14^C investigation of regional material culture from three urnfields in Jutland. By adopting a dynamic modelling approach, we can provide a dynamic perspective on the material culture, showing when certain artefact types were in production and primary use, how quickly types were taken up and later abandoned, and demonstrating periods with faster and slower change. We provide the first absolute correlation of metalwork and pottery artefact typologies, without which it is difficult to compare burial and settlement data.

We present the first absolute chronology for the period and demonstrate that the Bronze-Iron Age transformation took place already in the 7^th^ century BC. This is significantly earlier than the previously assumed c.530-500 BC and revives the discussion of whether the final Bronze Age per. VI might be interpreted as a transitional phase to the Iron Age, or if per. VI and the start of the PRIA are overlapping periods, possibly with different geographical distributions. It is now possible to correlate the absolutely dated start of the Iron Age in Denmark with Central European chronologies, providing the basis for future studies addressing overarching questions regarding for example social structure, economy and religion.

The last millennium BC have previously been avoided by ^14^C researchers due to the Hallstatt plateau c.750-400 cal BC (although see [15]), but by providing start and end boundaries of the urnfields within c.40-80yr (68.3% probability), and artefact currencies within c.30-70yr (68.3% probability), we successfully demonstrate that calibration plateaus are no longer a ‘catastrophe’ for archaeological chronologies [53, 56]. We are however surprised to find that the inversion of the calibration curve c.320-200 cal BC poses a comparable challenge. Although the IntCal20 calibration dataset has an annual resolution back to 5000 cal BP it does not include high-resolution calibration data for the last centuries BC [51].

## Supporting information

S1 File Figs and Tables. Archaeological data and chronological modelling output.
(DOCX)

S2 File Bayesian chronological models. (DOCX)
