# Supplementary material for: Dividing time—An absolute chronological study of material culture from Early Iron Age urnfields in Denmark
Source: PLoS One. 2024 May 28;19(5):e0300649. doi: 10.1371/journal.pone.0300649 (PMC11132521; doi:10.1371/journal.pone.0300649)
Supplement: S1 File — Archaeological data and chronological modelling output. (DOCX) [file pone.0300649.s001.docx]

**Dividing time – an absolute chronological study of material culture from Early Iron Age urnfields in Denmark**

Helene Agerskov Rose^1,2^* ORCID id 0000-0003-1061-3129

John Meadows^1,3^ORCID id 0000-0002-4346-5591

# Supplementary Information

# S1.1. Material record

**Table S1.1.1. Metalwork types in English and Danish.**

| English term | Danish term |
| --- | --- |
| Pin with coiled head | Krølhovednål |
| Pin with circular head | Ringhovednål |
| Pin with bomb-shaped head | Bombehovednål |
| Holstein pin | Holstensk nål |
| Pin with grooved head | Rillehovednål |
| Winged head pin | Vingehovednål |
| Pin with rod-shaped head | Stavhovednål |
| Iron ring with eyelet | Øskenring |
| Tongue-shaped belt clasp | Tungeformet bæltehage |
| Triangular belt clasp | Trekantet bæltehage |
| Narrow belt clasp | Smal bæltehage |
| Iron ring with attached shank | Jernring med tvinge |

**Table S1.1.2. Pottery typology, descriptions of vessel neck shapes, after Jensen [1].**

| Code | Shape of vessel neck |
| --- | --- |
| A | Cylindrical |
| B | Conical |
| C | Concave |
| D | Rim, but no neck |
| E | Conical rim, but no neck |

**Table S1.1.3. Pottery typology, descriptions of vessel body shapes, after Jensen [1].**

| Code | Shape of vessel body |
| --- | --- |
| 11 | Double conical shape, widest point in the lower part of the vessel |
| 12 | Double conical shape, widest point in the central part of the vessel |
| 13 | Evenly curved vessel |
| 14 | Domed, widest point in the central part of the vessel |
| 15 | Domed, widest point in the upper part of the vessel |
| 16 | Domed lower body, straight upper body, widest point in the upper part of the vessel |
| 17 | Domed lower body, conical upper body, widest point in the upper part of the vessel |
| 18 | Domed lower body, widest point in the absolute upper part of the vessel |
| 19 | Slightly domed – lenticular shape |
| 20 | Domed, widest point in the central part of the body – almost round corpus shape |


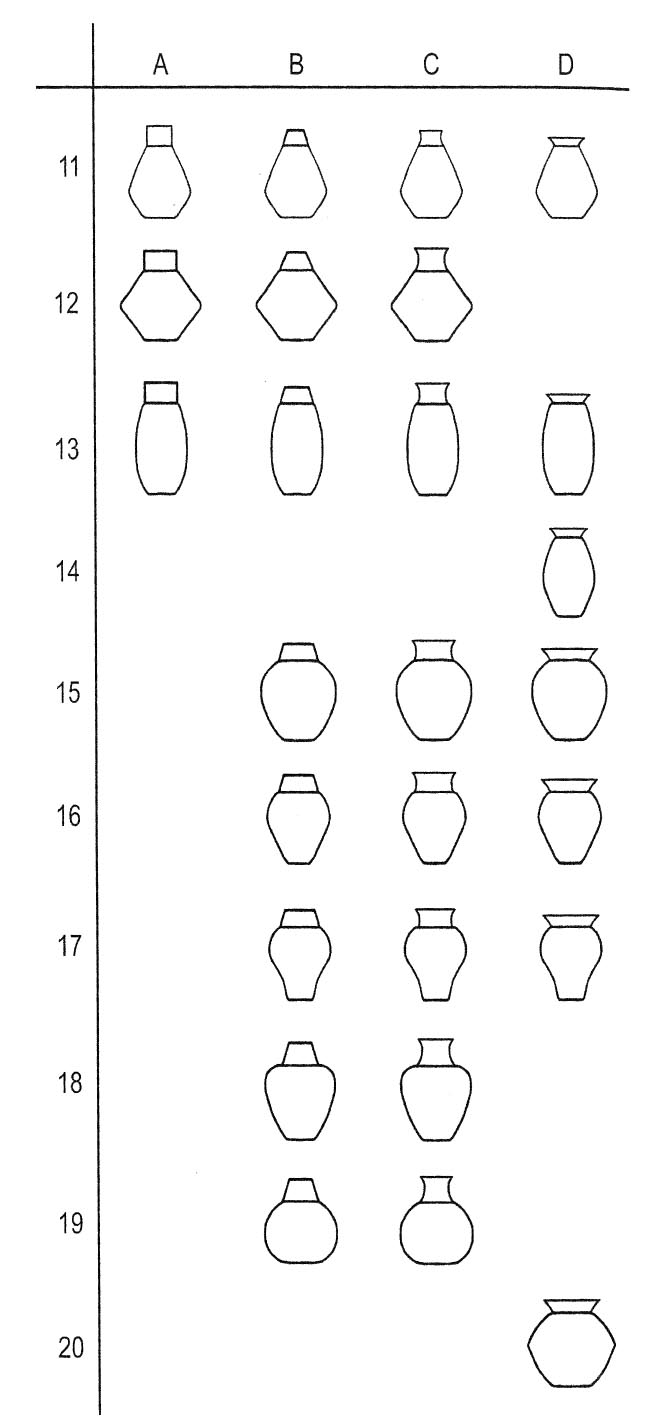


**Fig S1.1.1. Pottery typology after Jensen [1].**

# S1.2. Modelling burial activity


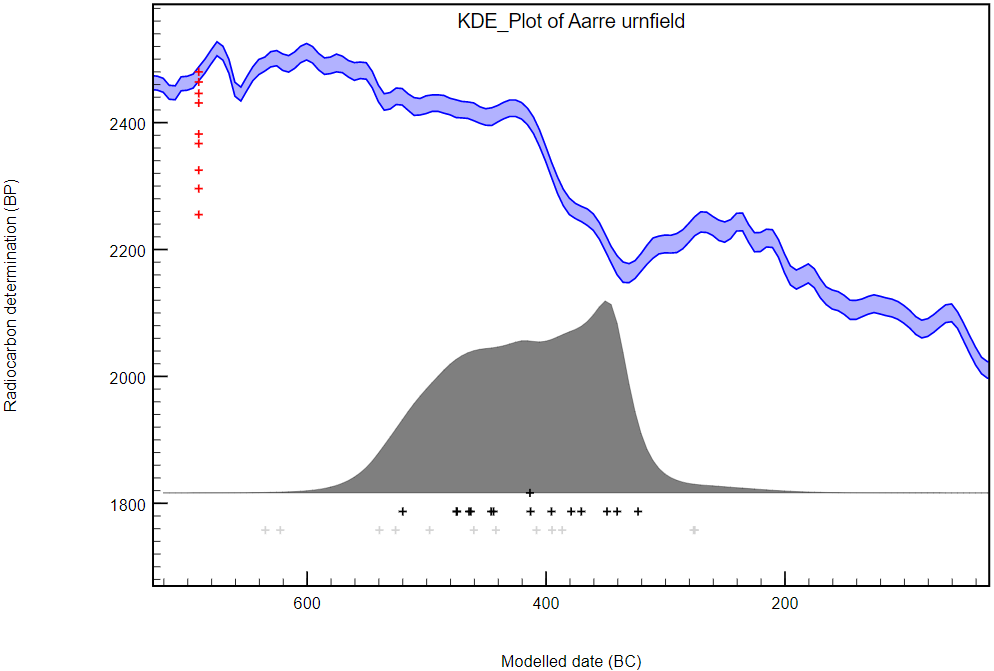


**Fig S1.2.1. Kernel density plot of Aarre urnfield.** Estimated by the preferred urnfield model B.


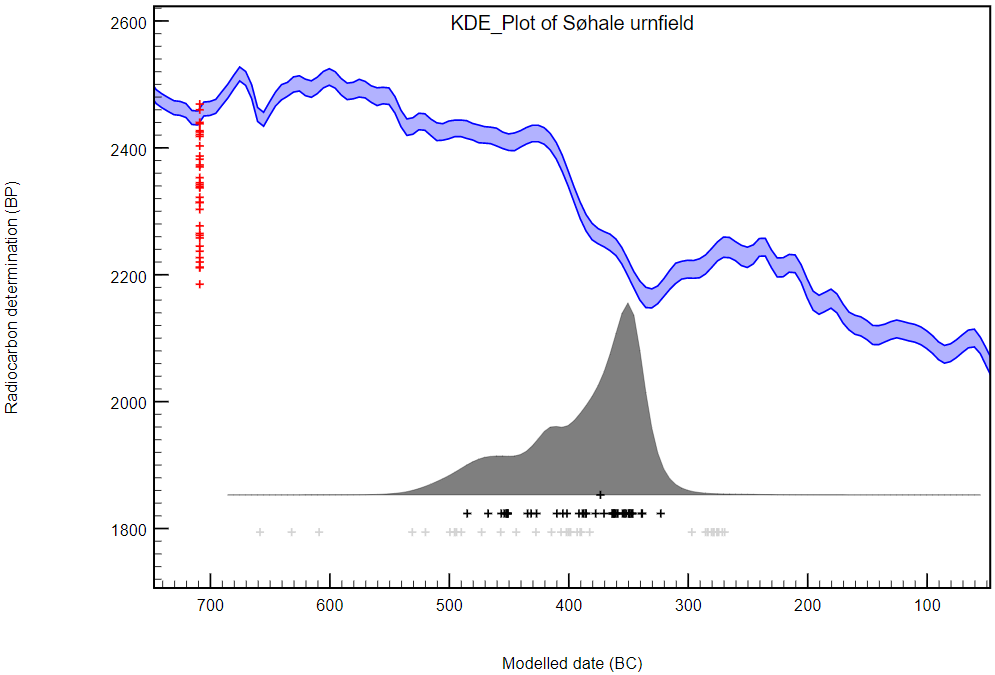


**Fig S1.2.2. Kernel density plot of Søhale urnfield.** Estimated by the preferred urnfield model B.


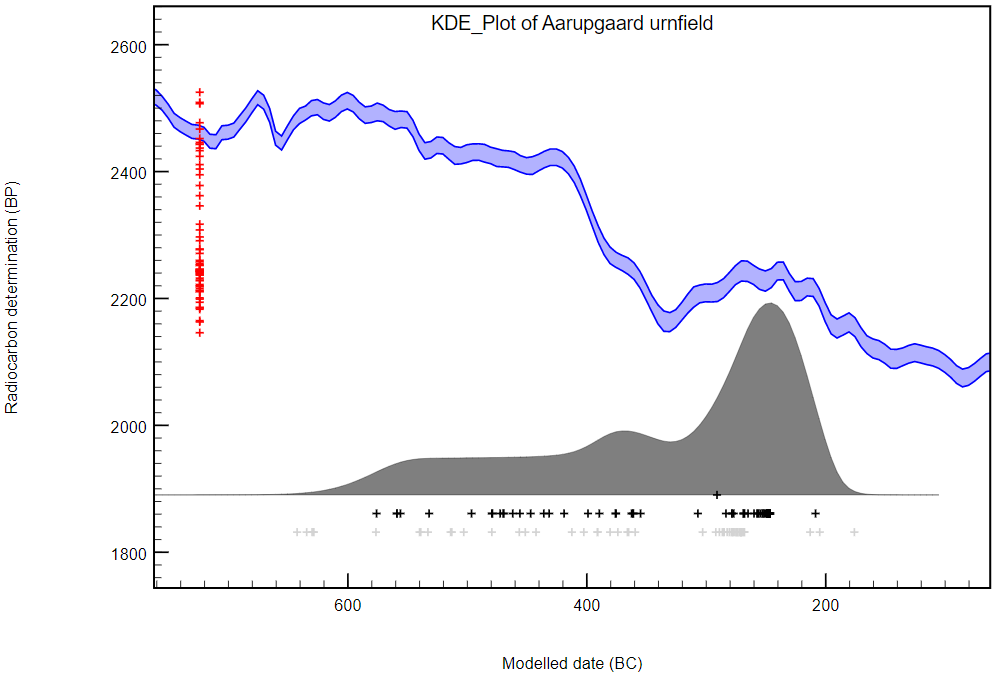


**Fig S1.2.3. Kernel density plot of Aarupgaard urnfield.** Estimated by the preferred urnfield model B.

## S1.3. Spatio-temporal development


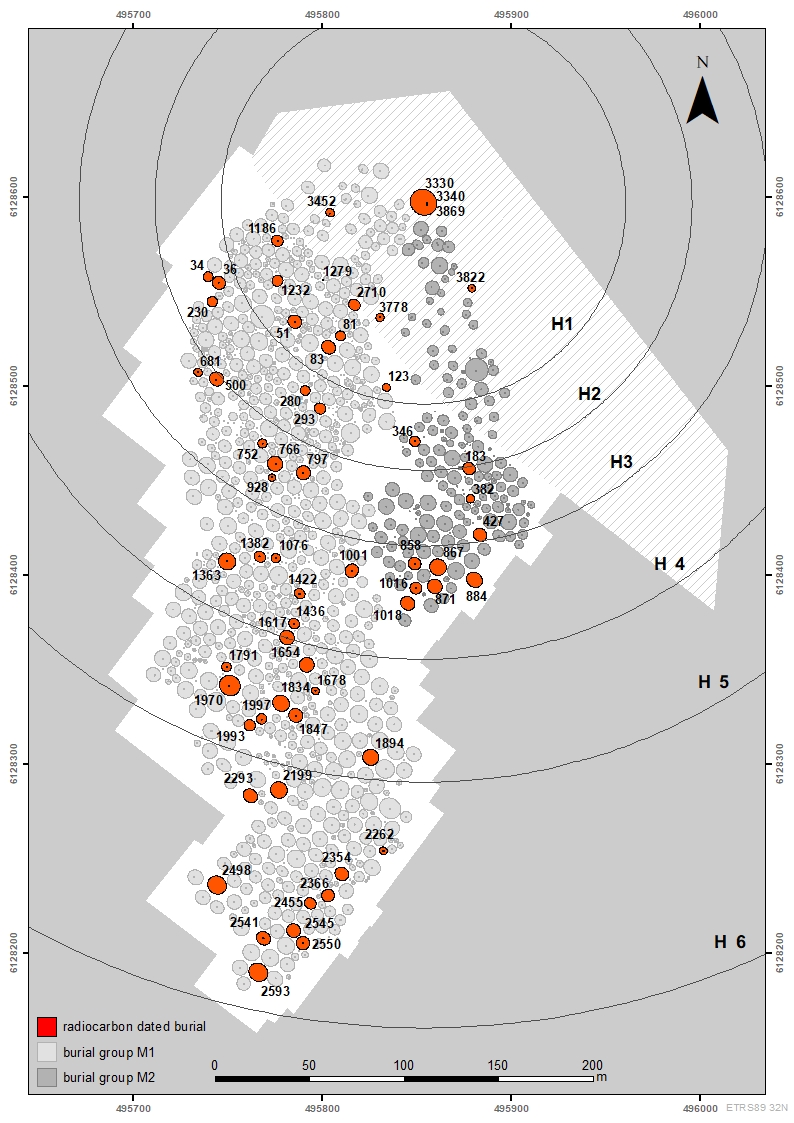


**Fig S1.3.1. Aarupgaard urnfield divided into arbitrary horizontal groups (H1-H6) of c.200 burials.** ^14^C dated burials are marked in red, burial group M1 in light grey and burial group M2 in dark grey.


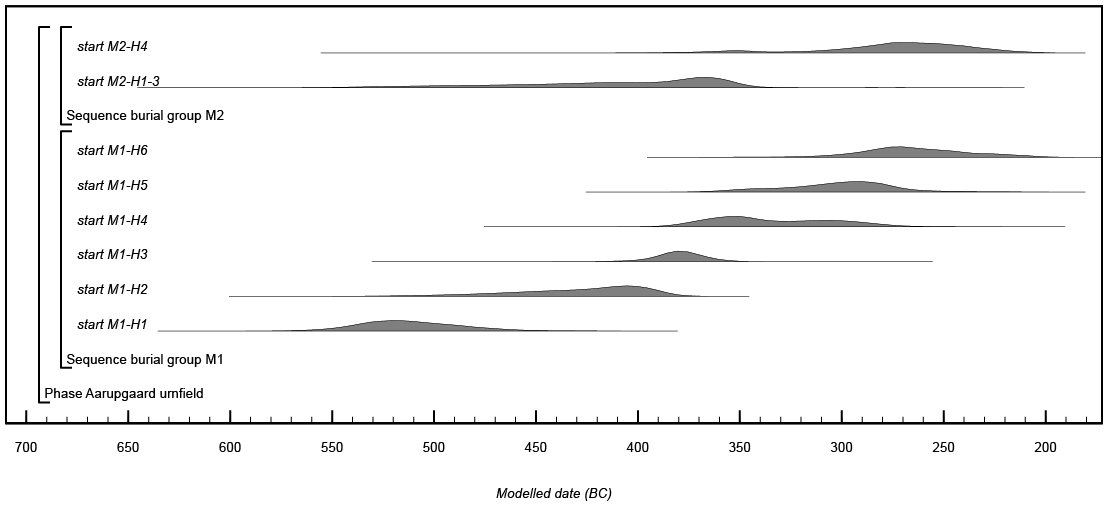
 **Fig S1.3.2. Posterior estimated starts of arbitrary horizontal groups at Aarupgaard urnfield.** Excluding the initial ‘founding phase’ with burials U3330, U3341 and U3869.


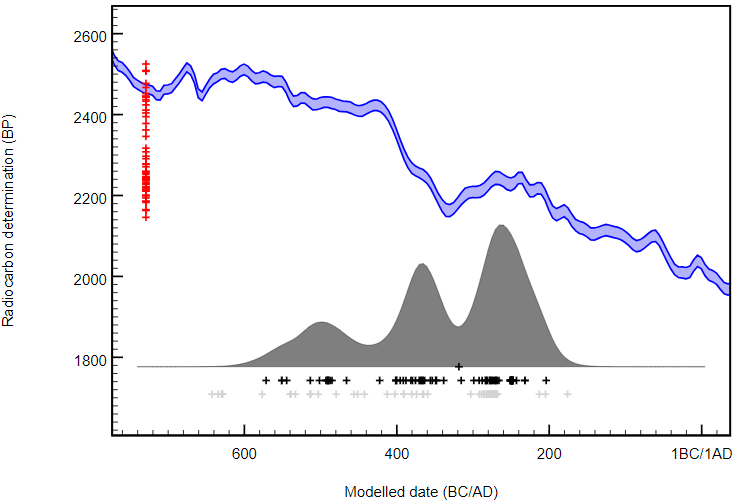


**Fig S1.3.3. Kernel density estimate of alternative horizontal model of Aarupgaard urnfield.**

## S1.4. Sensitivity testing


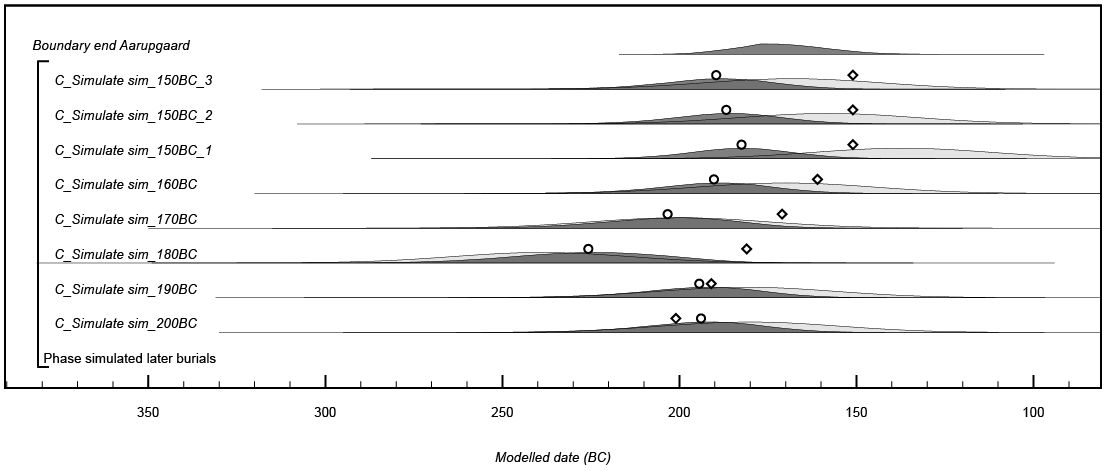
 **Fig S1.4.1. Adding simulated later burial dates to preferred urnfield model B.** Diamonds are medians of true calendar dates, circles are medians of posterior estimates.

## S1.5. Modelling artefact currencies

**Table S1.5.1. Graves containing possible heirlooms.** The table includes graves containing artefacts with posterior estimated outlier probabilities >5% as estimated by a General Outlier_Model [2]. All artefacts from the respective graves are included, but only outliers are marked in red. Identified heirlooms with residence offsets are marked in bold red.

| **Grave** | **object 1** | **object 2** | **object 3** | **object 4** |
| --- | --- | --- | --- | --- |
| Søhale x18 | Narrow (8%) |  |  |  |
| Søhale x30 | Eye let ring | Triangular (29%) |  |  |
| Søhale x38 | Narrow (10%) |  |  |  |
| Søhale x41 | Circular | Narrow (11%) |  |  |
| Søhale x65 | Eyelet ring | **Type 1 pin (18%)** |  |  |
| Aarre A99 | **Type 1 pin (34%)** |  |  |  |
| Aarre A155 | Circular (70%) |  |  |  |
| Aarre A281 | Circular (11%) |  |  |  |
| Aarupgaard U36 | 20B (6%) | Type 2 |  |  |
| Aarupgaard U51 | 15B (6%) | Type 1 |  |  |
| Aarupgaard U83 | 12B (6%) | Type 2 |  |  |
| Aarupgaard U123 | 20B | Circular | Triangular (9%) |  |
| Aarupgaard U183 | 12B (7%) | **Circular (11%)** | Tongue-shaped |  |
| Aarupgaard U293 | 20B | Circular (7%) |  |  |
| Aarupgaard U928 | 15B | **Type 2 (55%)** | Circular |  |
| Aarupgaard U1186 | 13A | Type 1 | Simple iron ring (6%) |  |
| Aarupgaard U1382 | 18C | **Circular (32%)** | Triangular |  |
| Aarupgaard U1617 | 18C | **Circular (62%)** | Triangular | Pin with grooved head |
| Aarupgaard U1834 | 12B (8%) | Tongue-shaped |  |  |
| Aarupgaard U2366 | **20B (17%)** | Simple iron ring (6%) | Pin with rod-shaped head |  |
| Aarupgaard U2455 | Simple iron ring (6%) | Pin with rod-shaped head |  |  |
| Aarupgaard U3452 | 15B (18%) | Type 1 (19%) |  |  |


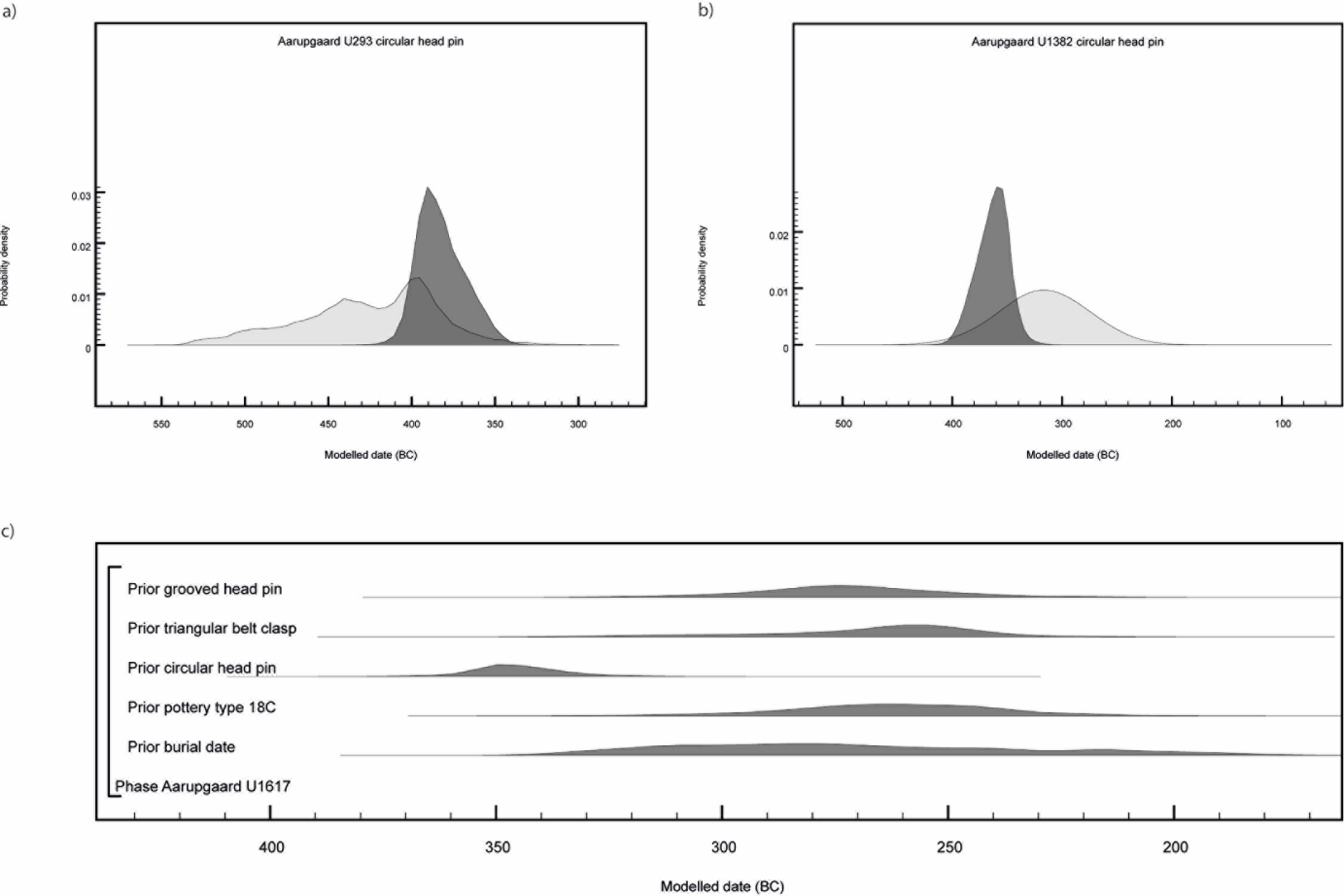
 **Fig S1.5.1. Identifying residence offsets.** Posterior estimated dates from preferred currency model with a General Outlier_Model [2]. a) circular head pin from Aarupgaard U293 estimated to be later than its burial date, b) circular head pin from Aarupgaard U1382 estimated to be earlier than its burial date, c) posterior estimated dates from Aarupgaard U1617, demonstrating the circular head pin to be an heirloom with a considerable residence offset.


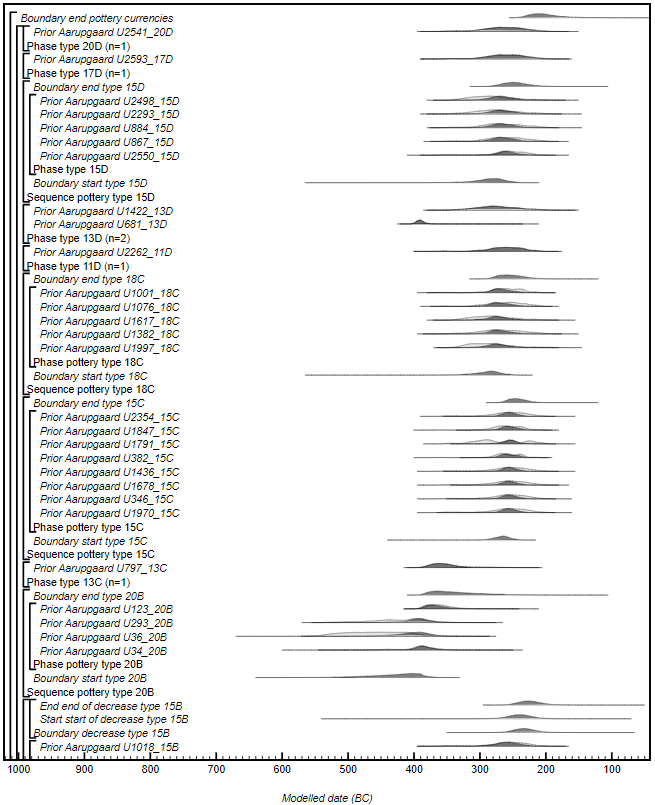
 **Fig S1.5.2. Chronological model of pottery currencies (1/2).**


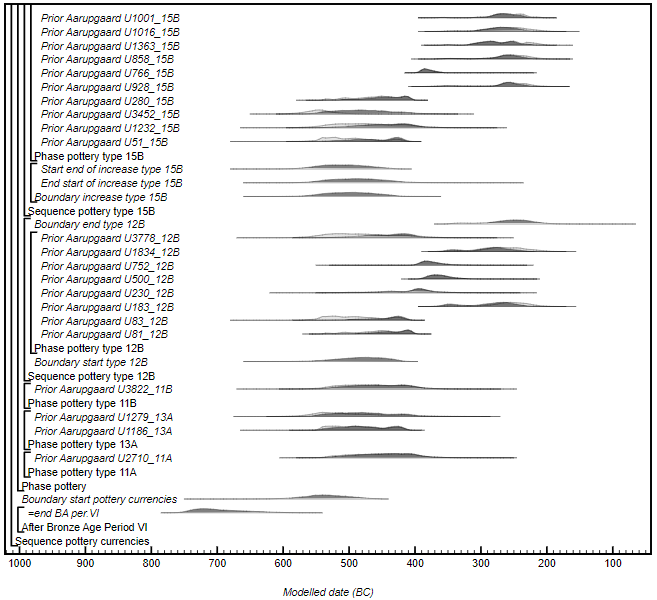


**Fig S1.5.2. Chronological model of pottery currencies (2/2).**


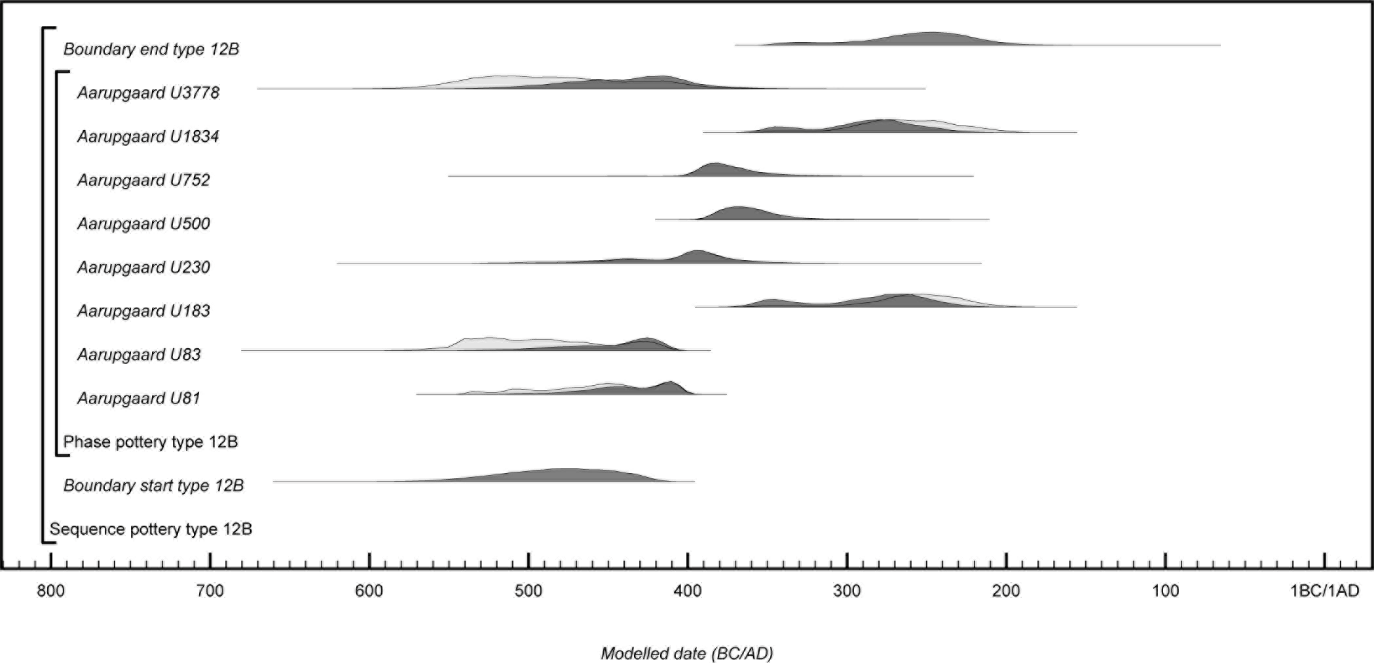
 **Fig S1.5.3. Currency model of pottery type 12B.**



 **Fig S1.5.4. Currency model of pottery type 15B.** Example from grave U766 from Aarupgaard urnfield.



 **Fig S1.5.5. Currency model of pottery type 15C.** Example from grave U346 from Aarupgaard urnfield.


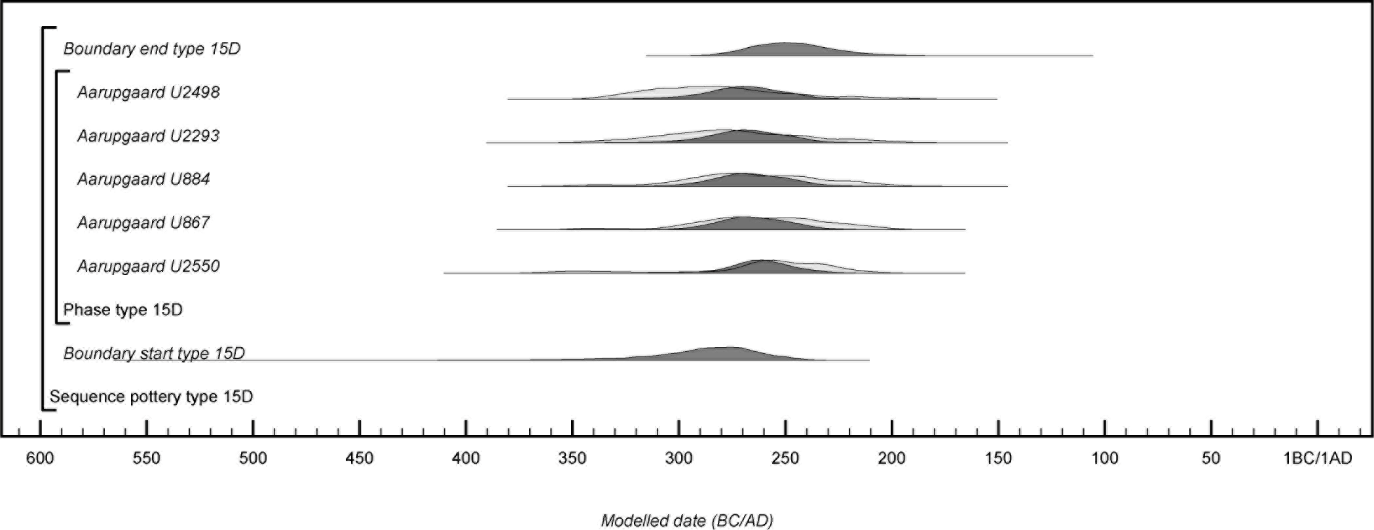
 **Fig S1.5.6. Currency model of pottery type 15D.**



 **Fig S1.5.7. Currency model of pottery type 18C.** Example from grave U1001 from Aarupgaard urnfield.



 **Fig S1.5.8. Currency model of pottery type 20B.** Example from grave U36 from Aarupgaard urnfield.


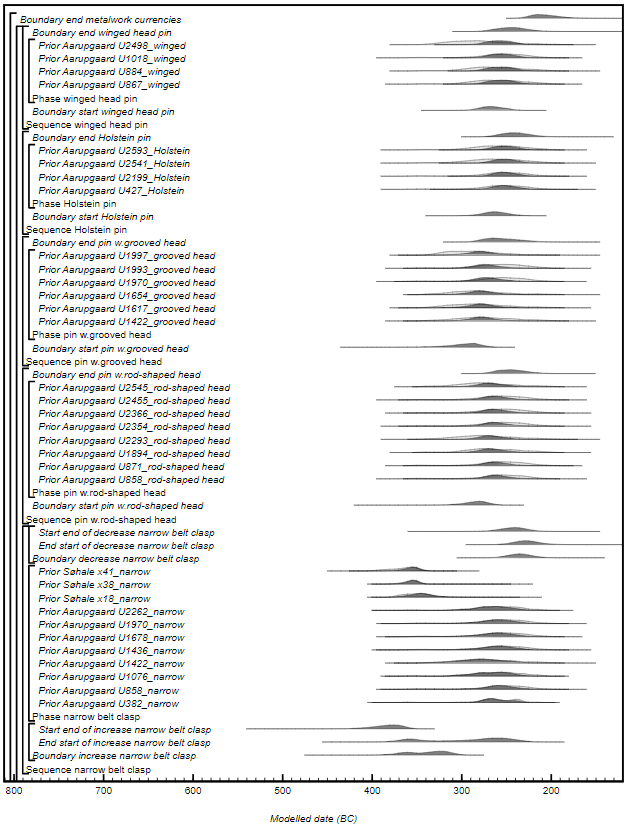
 **Fig S1.5.9 Chronological model of metalwork currencies (1/4).**


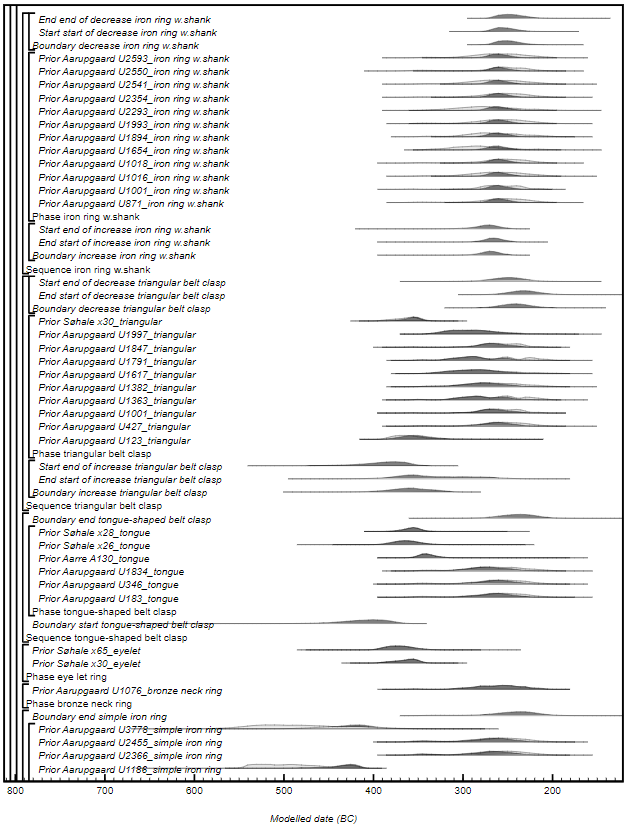
 **Fig S1.5.9. Chronological model of metalwork currencies (2/4).**


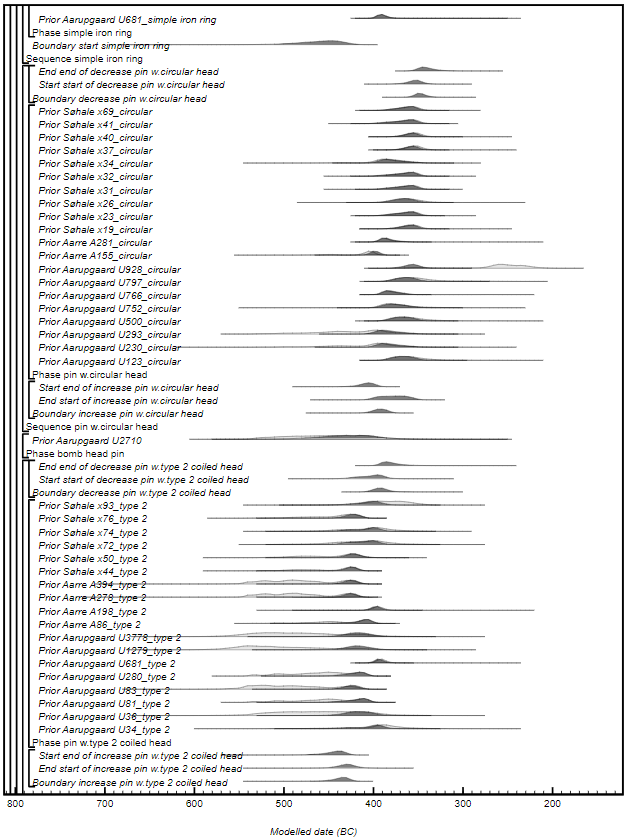
 **Fig S1.5.9. Chronological model of metalwork currencies (3/4).**


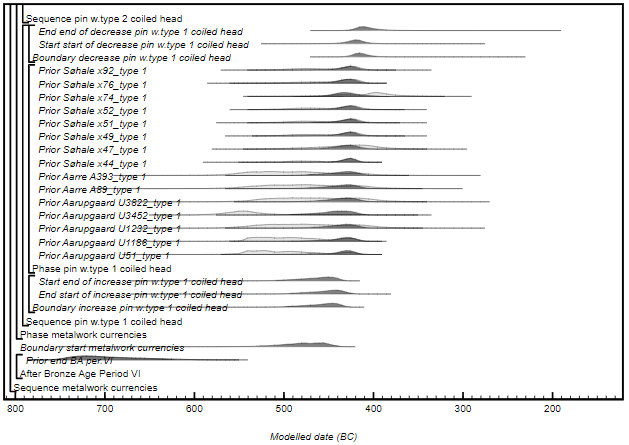
 **Fig S1.5.9. Chronological model of metalwork currencies (4/4).**


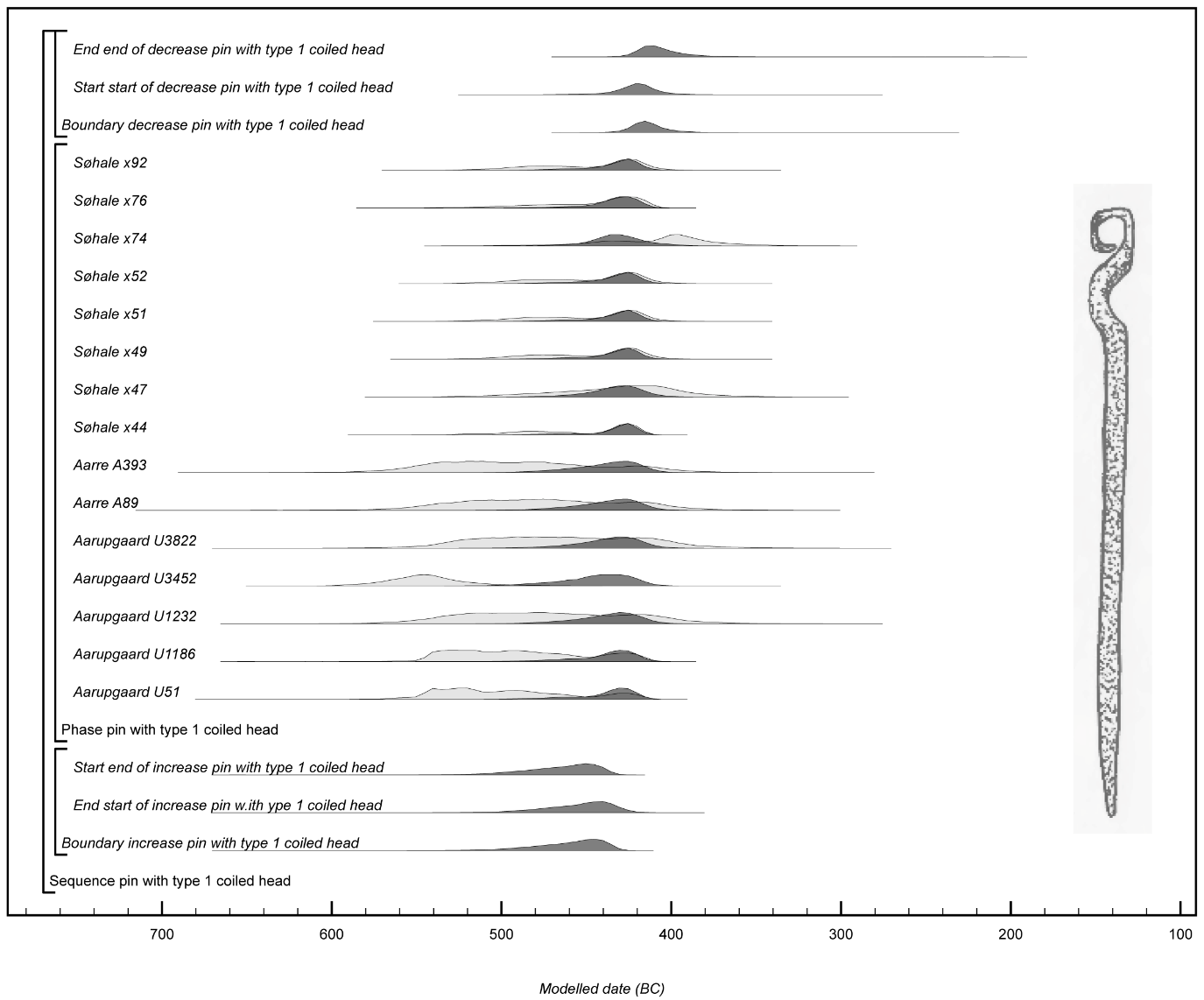
 **Fig S1.5.10. Currency model of pin with type 1 coiled head.** Example from grave U1186 from Aarupgaard urnfield.



 **Fig S1.5.11. Currency model of pin with type 2 coiled head.** Example from grave U81 from Aarupgaard urnfield.



 **Fig S1.5.12. Currency model of pin with circular head.** Example from grave U500 from Aarupgaard urnfield.


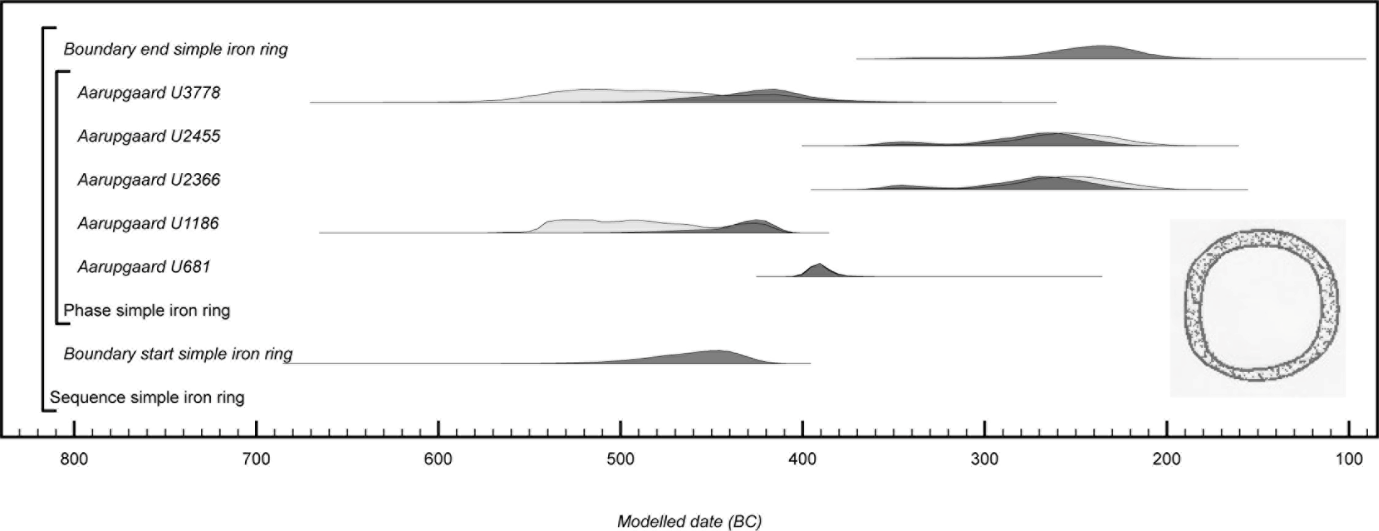
 **Fig S1.5.13. Currency model of simple iron ring.** Example from grave U1186 from Aarupgaard urnfield.



 **Fig S1.5.14. Currency model of tongue-shaped belt clasp.** Example from grave U1834 from Aarupgaard urnfield.



 **Fig S1.5.15. Currency model of triangular belt clasp.** Example from grave U1363 from Aarupgaard urnfield.



 **Fig S1.5.16. Currency model of narrow belt clasp.** Example from grave U1678 from Aarupgaard urnfield.



 **Fig S1.5.17. Currency model of iron ring with shank.** Example from grave U2550 from Aarupgaard urnfield.


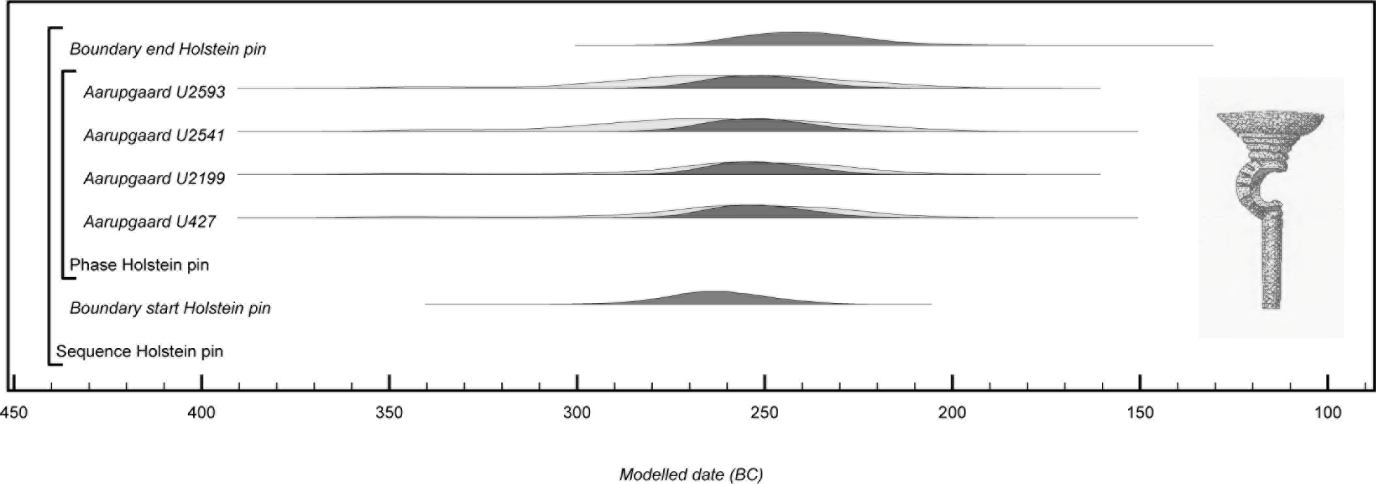
 **Fig S1.5.18. Currency model of Holstein pin.** Example from grave U2541 from Aarupgaard urnfield.


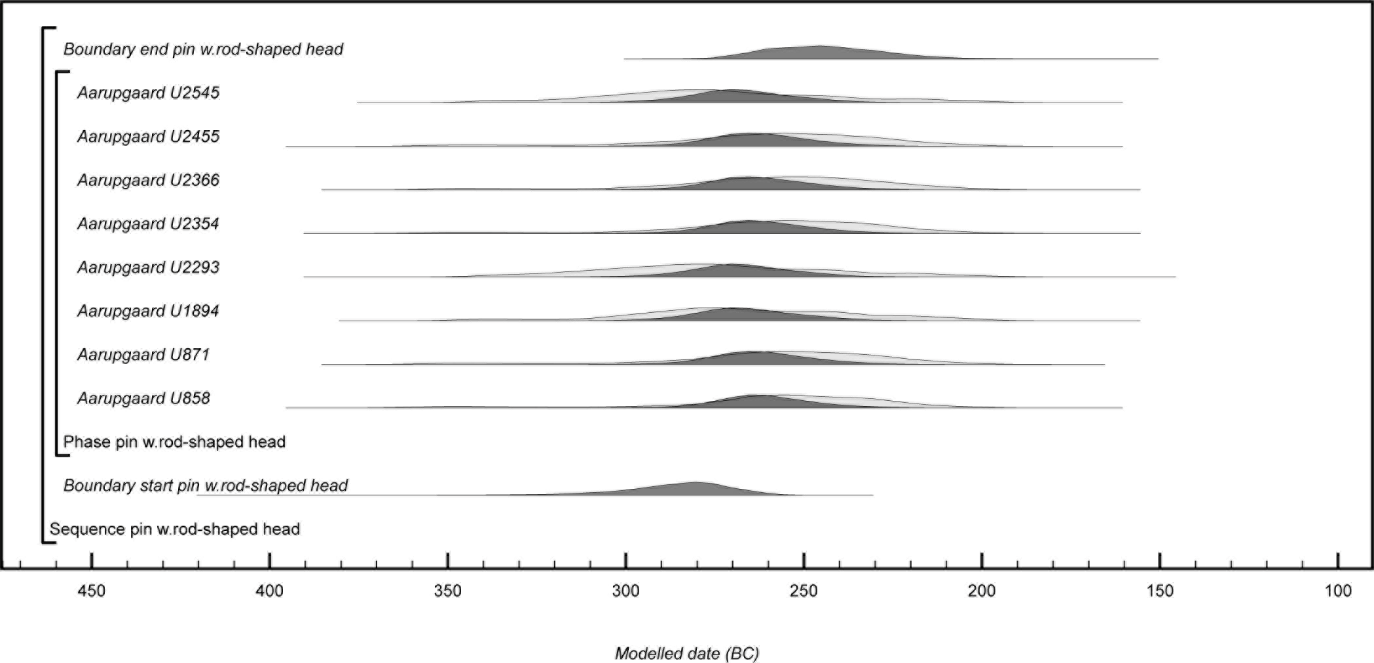
 **Fig S1.5.19. Currency model of pin with rod-shaped head.**


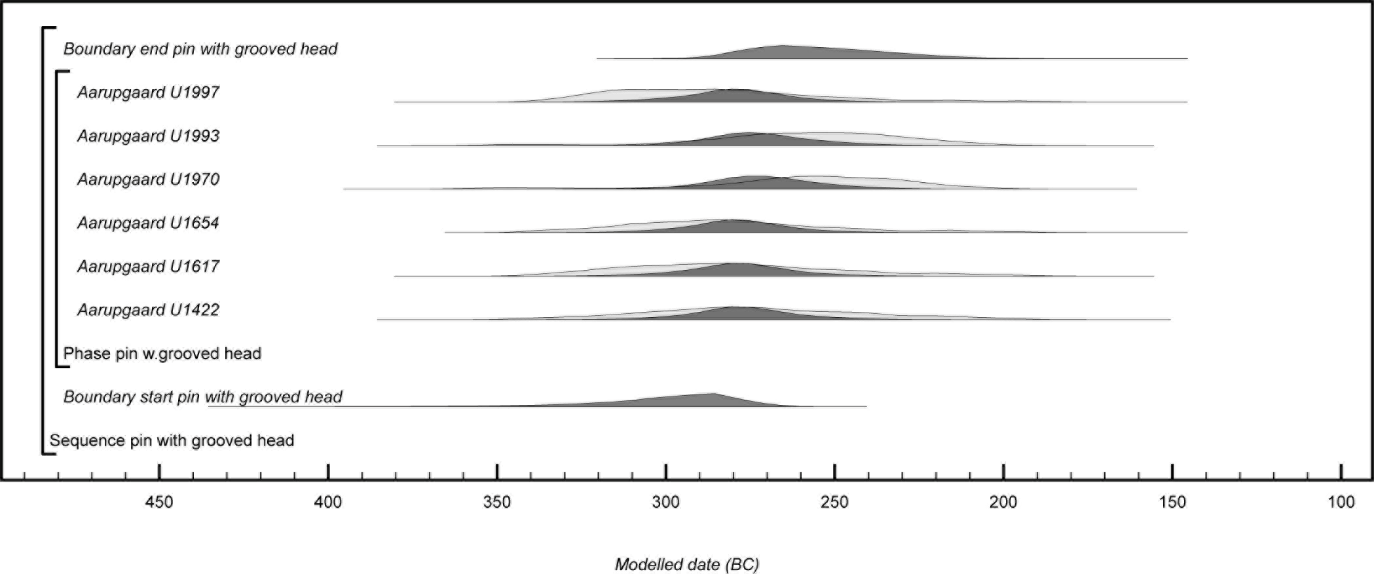
 **Fig S1.5.20. Currency model of pin with rod-shaped head.**


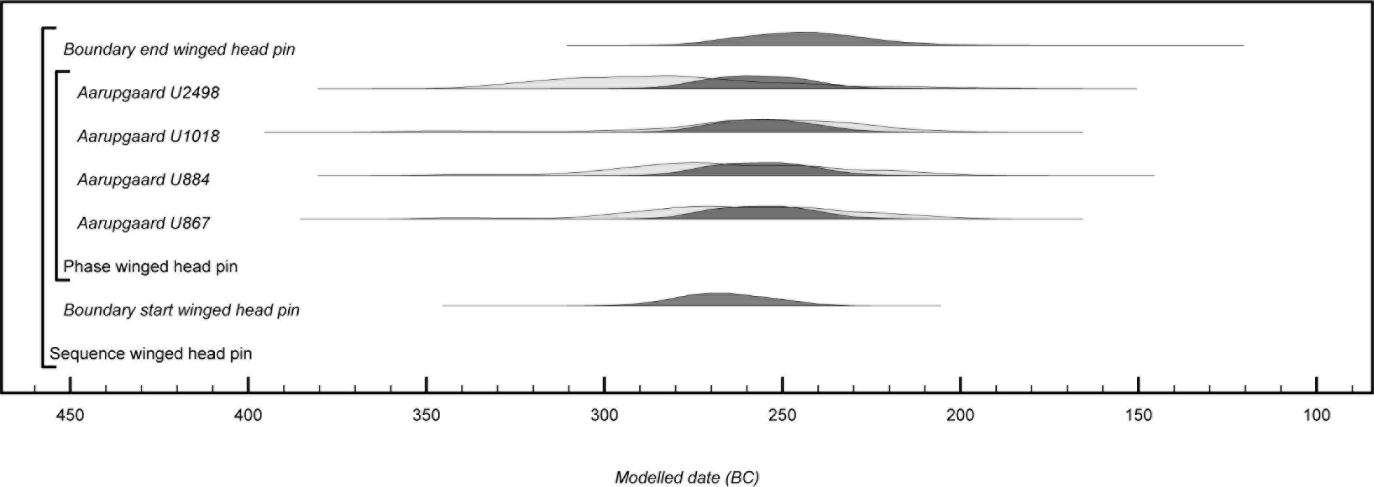
 **Fig S1.5.21. Currency model of winged head pin.**

**Fig SI.5.22. Circular head pins from Aarupgaard urnfield.** Plotted are the head size index against mean ages estimated by the currency model.

## S1.6. Discussion

| a) | 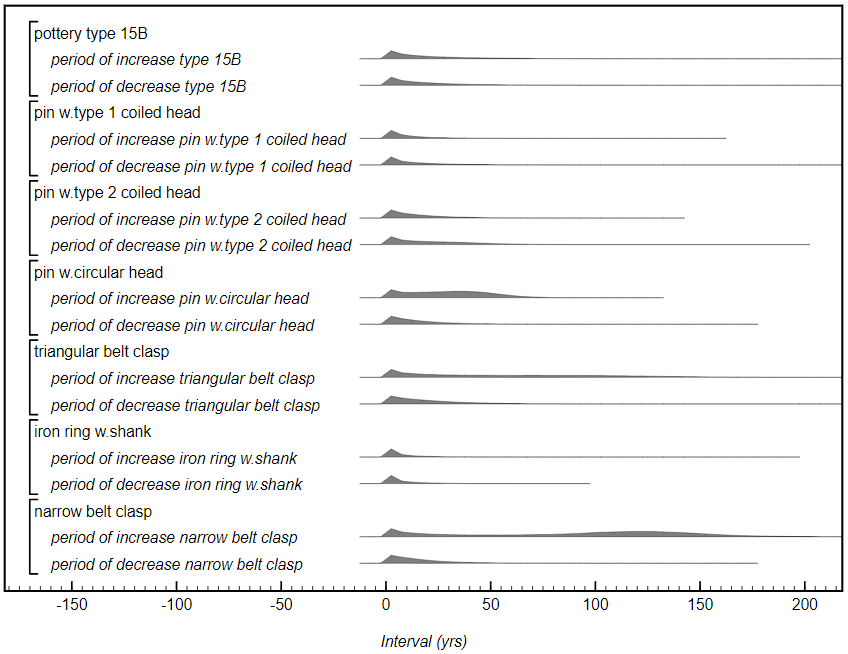 |
| --- | --- |
| b) | 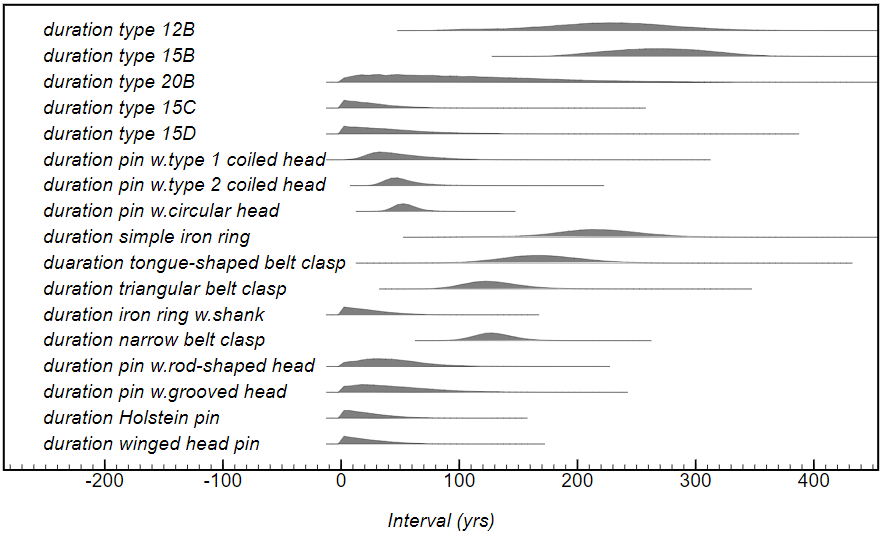 |

**Fig S1.6.1. Posterior estimated parameters from currency models.** a) Periods of production increase and decrease estimated by trapezium prior models, b) duration of currencies modelled in bounded phases.


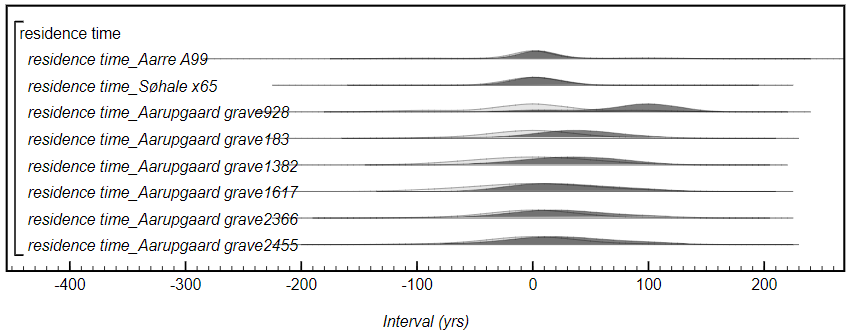


**Fig SI.6.2. Posterior estimated residence time.**

## References

1. Jensen CK. Kontekstuel kronologi: en revision af det kronologiske grundlag for førromersk jernalder i Sydskandinavien. Højbjerg: Kulturlaget; 2005.

2. Bronk Ramsey C. Dealing with Outliers and Offsets in Radiocarbon Dating. Radiocarbon. 2009; 51(3): 1023-45. <https://doi.org/10.1017/S0033822200034093>
