# Supplementary material for: Dividing time—An absolute chronological study of material culture from Early Iron Age urnfields in Denmark
Source: PLoS One. 2024 May 28;19(5):e0300649. doi: 10.1371/journal.pone.0300649 (PMC11132521; doi:10.1371/journal.pone.0300649)
Supplement: S2 File — (DOCX) [file pone.0300649.s002.docx]

**Dividing time – an absolute chronological study of material culture from Early Iron Age urnfields in Denmark**

Helene Agerskov Rose^1,2^* ORCID id 0000-0003-1061-3129

John Meadows^1,3^ORCID id 0000-0002-4346-5591

Supplementary Information

# S2. OxCal model code

The following CQL code will run in OxCal v4 [1], which is available online at <https://c14.arch.ox.ac.uk/>. Full details of how the program works and what individual functions are designed to do is given at <http://c14.arch.ox.ac.uk/oxcalhelp/hlp_contents.html>. A full bibliography is also provided. Please contact the corresponding author if you experience difficulties using the models.

# S2.1. Urnfield model A

Plot()

{

MCMC_Sample()

{

};

Outlier_Model ("cremation", Exp(0.9,-10,-0.1), U(1,3),"t");

Sequence("BA per.V-VI")

{

Boundary("start BA per.V");

Phase("BA per.V")

{

R_Date("AAR-8786(PB4) Ø.Herup CB", 2722, 25)

{

Outlier("cremation", 1);

};

R_Date("AAR-8787 (PB5) Sevel Kalkværk CB", 2650, 25)

{

Outlier("cremation", 1);

};

R_Date("AAR-9520 (PB24) Jersild CB", 2683, 36)

{

Outlier("cremation", 1);

};

Combine("Gl. Brydegård")

{

R_Date("AAR-9570 (PB32) Gl. Brydegård pitch", 2706, 35);

R_Date("AAR-9576 (PB38) Gl. Brydegård CB", 2714, 34)

{

Outlier("cremation", 1);

};

};

R_Date("AAR-9575 (PB37) Lusehøj GX CB", 2611, 33)

{

Outlier("cremation", 1);

};

};

Boundary("Transition V/VI");

Phase("BA VI")

{

R_Date("AAR-9571 (PB33) Lerbjerg I CB", 2486, 25)

{

Outlier("cremation", 1);

};

R_Date("AAR-9568 (PB30) Lerbjerg I pitch", 2851, 24)

{

Outlier();

};

Combine("Lerbjerg II")

{

R_Date("AAR-9569 (PB31) Lerbjerg II pitch", 2459, 41);

R_Date("AAR-9573 (PB35) Lerbjerg II CB", 2502, 39)

{

Outlier("cremation", 1);

};

};

};

Boundary("end BA per.VI");

};

Page( );

Sequence("urnfields")

{

After("Bronze Age Period VI")

{

Date("=end BA per.VI");

};

Boundary("start urnfields");

Phase("urnfields")

{

Sequence ("Aarre urnfield")

{

Boundary("start Aarre");

Phase ("Aarre urnfield cemetery")

{

R_Date("Aarre A89 RICH-25356", 2464, 27)

{

Outlier("cremation", 1);

};

R_Date("Aarre A281 comb", 2296, 15)

{

Outlier("cremation", 1);

};

Sequence ("A86")

{

After ("older CC from A86")

{

R_Date ("A86 KIA-53941", 2463, 25);

};

Phase("A86")

{

R_Date ("Aarre A86 comb KIA-53942", 2382, 19)

{

Outlier ("cremation", 1);

};

};

};

Combine("A95")

{

R_Date ("RICH-25342 CB", 2428, 27)

{

Outlier ("cremation", 1);

};

R_Date ("A95 twigs comb", 2399, 19);

};

Sequence ("A99")

{

After ("older CC from A99")

{

R_Date ("A99 RICH-25071", 2269, 29);

R_Date ("A99 RICH-25066", 2251, 30);

R_Date ("A99 RICH-25067", 3115, 31);

};

Phase("A99")

{

R_Date ("Aarre A99 GrM-16774", 2255, 20)

{

Outlier ("cremation", 1);

};

R_Date ("RICH-25069", 2085, 29)

{

Outlier();

};

};

};

Sequence ("A117")

{

After ("older CC from A117")

{

R_Date ("A117 KIA-53943", 2449, 25);

R_Date ("A117 KIA-53944", 2495, 24);

};

Phase("A117")

{

R_Date ("Aarre A117 CB comb", 2431, 15)

{

Outlier ("cremation", 1);

};

};

};

Sequence ("A130")

{

After ("older CC from A130")

{

R_Date ("A130 KIA-53945", 2585, 25);

};

Combine("A130")

{

R_Date ("Aarre A130 KIA-53947", 2253, 18)

{

Outlier ("cremation", 1);

};

R_Date ("A130 KIA-53946", 2156, 24);

};

};

Sequence ("A155")

{

After ("older CC from A155")

{

R_Date ("A155 KIA-53948", 2494, 24);

R_Date ("A155 KIA-53949", 2466, 24);

};

Phase("A155")

{

R_Date ("Aarre A155 comb KIA-53950", 2367, 18)

{

Outlier ("cremation", 1);

};

};

};

Sequence ("A198")

{

After ("older CC from A198")

{

R_Date ("A198 KIA-53951", 2967,24);

};

Phase("A198")

{

R_Date ("Aarre A198 comb KIA-53952", 2325, 21)

{

Outlier ("cremation", 1);

};

};

};

Combine("A278")

{

R_Date ("Aarre A278 KIA-53955", 2463, 19)

{

Outlier ("cremation", 1);

};

R_Date ("A278 grass comb", 2423, 18);

};

Sequence ("A393")

{

After ("older CC from A393")

{

R_Date ("A393 RICH-25068", 2901, 32);

R_Date ("A393 RICH-25070", 2914, 32);

R_Date ("A393 KIA-52411", 3134, 25);

R_Date ("A393 KIA-52412", 3150, 27);

R_Date ("A393 KIA-52413", 2611, 27);

};

Phase("A393")

{

R_Date ("Aarre A393 RICH-25341", 2480, 27)

{

Outlier ("cremation", 1);

};

};

};

Sequence ("A394")

{

After ("older CC from A394")

{

R_Date ("A394 KIA-52414", 2778, 27);

R_Date ("A394 KIA-52415", 2843, 26);

R_Date ("A394 KIA-52416", 2772, 26);

R_Date ("A394 KIA-52417", 2719, 27);

R_Date ("A394 KIA-53983", 3029, 24);

};

Phase("A394")

{

R_Date ("Aarre A394 comb", 2446,14)

{

Outlier ("cremation", 1);

};

};

};

};

Span("duration Aarre urnfield");

KDE_Plot("Aarre urnfield",);

Boundary("end Aarre");

};

Page( );

Sequence("Aarupgaard urnfield - entrance model")

{

Boundary("start Aarupgaard");

Phase("founding graves")

{

R_Date("Aarupgaard grave 3869", 2507, 14)

{

Outlier ("cremation", 1);

};

R_Date("Aarupgaard grave 3330 comb", 2509, 14)

{

Outlier ("cremation", 1);

};

R_Date("Aarupgaard grave 3340", 2477, 27)

{

Outlier ("cremation", 1);

};

};

Phase("multiple pathways")

{

R_Date("Aarupgaard grave 83 comb", 2443, 21)

{

Outlier ("cremation", 1);

};

R_Date("Aarupgaard grave 34", 2346, 28)

{

Outlier ("cremation", 1);

};

R_Date("Aarupgaard grave 36", 2424, 30)

{

Outlier ("cremation", 1);

};

R_Date("Aarupgaard grave 81 comb", 2395, 18)

{

Outlier ("cremation", 1);

};

R_Date("Aarupgaard grave 123", 2277, 26)

{

Outlier ("cremation", 1);

};

R_Date("Aarupgaard grave 230", 2362, 25)

{

Outlier ("cremation", 1);

};

R_Date("Aarupgaard grave 280 comb", 2404, 15)

{

Outlier ("cremation", 1);

};

R_Date("Aarupgaard grave 293", 2378, 23)

{

Outlier ("cremation", 1);

};

R_Date("Aarupgaard grave 500", 2278, 23)

{

Outlier ("cremation", 1);

};

R_Date("Aarupgaard grave 681 comb", 2308, 14)

{

Outlier ("cremation", 1);

};

R_Date("Aarupgaard grave 752", 2317, 26)

{

Outlier ("cremation", 1);

};

R_Date("Aarupgaard grave 766 comb", 2271, 16)

{

Outlier ("cremation", 1);

};

R_Date("Aarupgaard grave 797", 2255, 28)

{

Outlier ("cremation", 1);

};

R_Date("Aarupgaard grave 1186 comb", 2437, 15)

{

Outlier ("cremation", 1);

};

R_Date("Aarupgaard grave 1279", 2467, 26)

{

Outlier ("cremation", 1);

};

R_Date("Aarupgaard grave 3452", 2525, 25)

{

Outlier ("cremation", 1);

};

R_Date("Aarupgaard grave 3778", 2452, 25)

{

Outlier ("cremation", 1);

};

R_Date("Aarupgaard grave 51 comb", 2446, 16)

{

Outlier ("cremation", 1);

};

R_Date("Aarupgaard grave 2710", 2411, 27)

{

Outlier ("cremation", 1);

};

R_Date("Aarupgaard grave 3822", 2433, 26)

{

Outlier ("cremation", 1);

};

R_Date("Aarupgaard grave 1232", 2443, 27)

{

Outlier ("cremation", 1);

};

};

Phase("2 pathways")

{

R_Date("Aarupgaard grave 183", 2243, 23)

{

Outlier ("cremation", 1);

};

R_Date("Aarupgaard grave 346", 2246, 24)

{

Outlier ("cremation", 1);

};

R_Date("Aarupgaard grave 382 comb", 2260, 16)

{

Outlier ("cremation", 1);

};

R_Date("Aarupgaard grave 858", 2258, 23)

{

Outlier ("cremation", 1);

};

R_Date("Aarupgaard grave 928", 2297, 25)

{

Outlier ("cremation", 1);

};

R_Date("Aarupgaard grave 1001 comb", 2244, 15)

{

Outlier ("cremation", 1);

};

R_Date("Aarupgaard grave 1016", 2220, 25)

{

Outlier ("cremation", 1);

};

R_Date("Aarupgaard grave 1076 comb", 2229, 15)

{

Outlier ("cremation", 1);

};

R_Date("Aarupgaard grave 1363 comb", 2213, 16)

{

Outlier ("cremation", 1);

};

R_Date("Aarupgaard grave 1382", 2201, 27)

{

Outlier ("cremation", 1);

};

R_Date("Aarupgaard grave 1422", 2186, 26)

{

Outlier ("cremation", 1);

};

R_Date("Aarupgaard grave 1436", 2252, 28)

{

Outlier ("cremation", 1);

};

R_Date("Aarupgaard grave 1617", 2163, 28)

{

Outlier ("cremation", 1);

};

R_Date("Aarupgaard grave 1678", 2243, 25)

{

Outlier ("cremation", 1);

};

R_Date("Aarupgaard grave 1791 comb", 2199, 18)

{

Outlier ("cremation", 1);

};

R_Date("Aarupgaard grave 1847 comb", 2244, 16)

{

Outlier ("cremation", 1);

};

R_Date("Aarupgaard grave 1970", 2246, 23)

{

Outlier ("cremation", 1);

};

R_Date("Aarupgaard grave 1997", 2146, 27)

{

Outlier ("cremation", 1);

};

R_Date("Aarupgaard grave 2262 comb", 2237, 19)

{

Outlier ("cremation", 1);

};

R_Date("Aarupgaard grave 2541", 2216, 26)

{

Outlier ("cremation", 1);

};

R_Date("Aarupgaard grave 2550", 2291, 26)

{

Outlier ("cremation", 1);

};

R_Date("KIA-55388_Aarupgaard grave 427",2241,17)

{

Outlier("cremation", 1);

};

R_Date("KIA-55389_Aarupgaard grave 867",2219,12)

{

Outlier("cremation", 1);

};

R_Date("KIA-55390_Aarupgaard grave 871",2239,17)

{

Outlier("cremation", 1);

};

R_Date("KIA-55391_Aarupgaard grave 884",2211,16)

{

Outlier("cremation", 1);

};

R_Date("KIA-55392_Aarupgaard grave 1018",2242,17)

{

Outlier("cremation", 1);

};

R_Date("KIA-55393_Aarupgaard grave 1654",2183,14)

{

Outlier("cremation", 1);

};

R_Date("Aarupgaard grave 1834 comb", 2253,15)

{

Outlier("cremation", 1);

};

R_Date("KIA-55395_Aarupgaard grave 1894",2211,14)

{

Outlier("cremation", 1);

};

R_Date("KIA-55396_Aarupgaard grave 1993",2228,15)

{

Outlier("cremation", 1);

};

R_Date("KIA-55397_Aarupgaard grave 2199",2244,17)

{

Outlier("cremation", 1);

};

R_Date("KIA-55398_Aarupgaard grave 2293",2185,25)

{

Outlier("cremation", 1);

};

R_Date("KIA-55399_Aarupgaard grave 2354",2237,18)

{

Outlier("cremation", 1);

};

R_Date("KIA-55400_Aarupgaard grave 2366",2232,15)

{

Outlier("cremation", 1);

};

R_Date("KIA-55401_Aarupgaard grave 2455",2240,25)

{

Outlier("cremation", 1);

};

R_Date("KIA-55402_Aarupgaard grave 2498",2165,25)

{

Outlier("cremation", 1);

};

R_Date("KIA-55403_Aarupgaard grave 2545",2194,18)

{

Outlier("cremation", 1);

};

R_Date("KIA-55404_Aarupgaard grave 2593",2222,17)

{

Outlier("cremation", 1);

};

};

Span("duration Aarupgaard urnfield");

KDE_Plot("Aarupgaard urnfield", );

Boundary("end Aarupgaard");

};

Page( );

Sequence("Søhale urnfield")

{

Boundary("start Søhale");

Phase("all burials")

{

R_Date("Søhale x47-III AAR-25258", 2421, 27)

{

Outlier ("cremation", 1);

};

R_Date("Søhale x34-II RICH-26502", 2342, 26)

{

Outlier ("cremation", 1);

};

R_Date("Søhale x33-II AAR-25256", 2353, 27)

{

Outlier ("cremation", 1);

};

R_Date("Søhale x55-II AAR-25262", 2345, 27)

{

Outlier ("cremation", 1);

};

Phase("no circular ditch")

{

Combine("Søhale x76-II comb")

{

R_Date("KIA-53434", 2434, 21)

{

Outlier("cremation", 1);

};

R_Date("KIA-53940", 2429, 26)

{

Outlier("cremation", 1);

};

};

R_Date("Søhale x72-VII KIA-53432", 2382, 21)

{

Outlier ("cremation", 1);

};

R_Date("Søhale x74-II KIA-53433", 2373, 21)

{

Outlier ("cremation", 1);

};

};

Phase("no entrances")

{

R_Date("Søhale x35-II AAR-25257", 2469, 26)

{

Outlier ("cremation", 1);

};

Combine("Søhale x44-II comb")

{

R_Date("KIA-53939", 2468, 25)

{

Outlier("cremation", 1);

};

R_Date("GrM-16772", 2465, 20)

{

Outlier("cremation", 1);

};

};

R_Date("Søhale x49-III GrM-16773", 2425, 19)

{

Outlier ("cremation", 1);

};

R_Date("Søhale x50-II KIA-53431", 2438, 21)

{

Outlier ("cremation", 1);

};

R_Date("Søhale x51-IV AAR-25260", 2440, 27)

{

Outlier ("cremation", 1);

};

R_Date("Søhale x52-II AAR-25261", 2418, 31)

{

Outlier ("cremation", 1);

};

R_Date("Søhale x92-III KIA-53435", 2427, 21)

{

Outlier ("cremation", 1);

};

};

Phase("N-S entrances")

{

R_Date("Søhale x26-III AAR-25252", 2277, 38)

{

Outlier ("cremation", 1);

};

R_Date("Søhale x48-II AAR-25259", 2460, 30)

{

Outlier ("cremation", 1);

};

R_Date("Søhale x21-III AAR-25250", 2258, 27)

{

Outlier ("cremation", 1);

};

R_Date("Søhale x93-II AAR-25265", 2387, 29)

{

Outlier ("cremation", 1);

};

R_Date("Søhale x65-V AAR-25263", 2314, 28)

{

Outlier ("cremation", 1);

};

R_Date("Søhale x18-III AAR-25249", 2185, 27)

{

Outlier ("cremation", 1);

};

};

Phase("NNE-SSW entrances")

{

R_Date("Søhale x22-III AAR-25251", 2211, 27)

{

Outlier ("cremation", 1);

};

R_Date("Søhale x32-II AAR-25255", 2403, 29)

{

Outlier ("cremation", 1);

};

R_Date("Søhale x14 AAR-25246", 2339, 26)

{

Outlier ("cremation", 1);

};

R_Date("Søhale x23-II RICH-26501", 2314, 24)

{

Outlier ("cremation", 1);

};

R_Date("Søhale x30-III AAR-25254", 2322, 28)

{

Outlier ("cremation", 1);

};

R_Date("Søhale x31-II RICH-26494", 2337, 27)

{

Outlier ("cremation", 1);

};

R_Date("Søhale x41-V GrM-16771", 2370, 20)

{

Outlier ("cremation", 1);

};

R_Date("Søhale x69-IV AAR-25264", 2303, 28)

{

Outlier ("cremation", 1);

};

R_Date("Søhale x10-II AAR-25243", 2212, 30)

{

Outlier ("cremation", 1);

};

Combine("Søhale x12/x25A comb")

{

R_Date("AAR-25244", 2181, 38)

{

Outlier("cremation", 1);

};

R_Date("AAR-25245", 2232, 34)

{

Outlier("cremation", 1);

};

R_Date("Søhale x17 AAR-25248", 2262, 40)

{

Outlier ("cremation", 1);

};

R_Date("Søhale x19-II KIA-53936", 2265, 26)

{

Outlier ("cremation", 1);

};

R_Date("Søhale x27-I AAR-25253",2237, 30)

{

Outlier ("cremation", 1);

};

R_Date("Søhale x28-II RICH-26493", 2245, 27)

{

Outlier ("cremation", 1);

};

R_Date("Søhale x37-III KIA-53937", 2220, 20)

{

Outlier ("cremation", 1);

};

Combine("Søhale x38-II comb")

{

R_Date("RICH-26495", 2254, 26)

{

Outlier("cremation", 1);

};

R_Date("KIA-53938", 2192, 27)

{

Outlier("cremation", 1);

};

};

R_Date("Søhale x40-II GrM-16770", 2227, 19)

{

Outlier ("cremation", 1);

};

};

};

Span ("Duration Søhale urnfield cemetery");

KDE_Plot("Søhale urnfield", );

Boundary("end Søhale");

};

};

Span ("duration urnfield burial activity");

KDE_Plot("urnfields", );

Boundary("end urnfields");

};

};

# S2.2. Urnfield model B

Plot()

{

MCMC_Sample()

{

};

Phase("Replicate measurements")

{

R_Combine("Aarre A281x484")

{

R_Date("GrM-14707 Aarre A281x484", 2320, 20);

R_Date("KIA-53100 Aarre A281x484", 2271, 20);

};

R_Combine("Aarre A95x368 twigs")

{

R_Date ("KIA-53984 A95x368 no.1", 2370, 25);

R_Date ("KIA-53985 A95x368 no.3", 2430, 26);

};

R_Combine("Aarre A86x340")

{

R_Date("KIA-53942_1 Aarre A86x340", 2379, 26);

R_Date("KIA-53942_2 Aarre A86x340", 2385, 25);

};

R_Combine("Aarre A117x762")

{

R_Date("GrM-14604 Aarre A117x762", 2445, 20);

R_Date("KIA-53098 Aarre A117x762", 2416, 20);

};

R_Combine("Aarre A155x281")

{

R_Date("KIA-53950_1 Aarre A155x281", 2359, 25);

R_Date("KIA-53950_2 Aarre A155x281", 2374, 25);

};

R_Combine("Aarre A198x338")

{

R_Date("KIA-53952_1 Aarre A198x338", 2323, 26);

R_Date("KIA-53952_2 Aarre A198x338", 2330, 35);

};

R_Combine("Aarre A278x783")

{

R_Date("KIA-53955_1 Aarre A278x783", 2477, 26);

R_Date("KIA-53955_2 Aarre A278x783", 2450, 25);

};

R_Combine("Aarre A278x782 grass")

{

R_Date("KIA-53953 Aarre A278x782 no.1", 2400, 25);

R_Date("KIA-53954 Aarre A278x782 no.2", 2445, 25);

};

R_Combine("Aarre A394x785")

{

R_Date("GrM-14708 Aarre A394x785 CB", 2465, 18);

R_Date("KIA-53099 Aarre A394x785 CB", 2422, 20);

};

R_Combine("Aarupgaard U3869")

{

R_Date("GrM-15076 Aarupgaard U3869", 2540, 20);

R_Date("RICH-24152 Aarupgaard U3869", 2504, 26);

};

R_Combine("Aarupgaard U3330")

{

R_Date("GrM-14594 Aarupgaard U3330", 2535, 20);

R_Date("KIA-51901 Aarupgaard U3330", 2503, 27);

R_Date("KIA 52824 Aarupgaard U3330", 2471, 26);

};

R_Combine("Aarupgaard U83")

{

R_Date("GrM-15078 Aarupgaard U83", 2485, 30);

R_Date("KIA-52825 Aarupgaard U83", 2409, 27);

};

R_Combine("Aarupgaard U81")

{

R_Date("GrM-14589 Aarupgaard U81", 2425, 30);

R_Date("KIA-52819 Aarupgaard U81", 2379, 22);

};

R_Combine("Aarupgaard U280")

{

R_Date("GrM-14588 Aarupgaard U280", 2405, 20);

R_Date("KIA-52820 Aarupgaard U280", 2402, 22);

};

R_Combine("Aarupgaard U681")

{

R_Date("GrM-14705 Aarupgaard U681", 2310, 19);

R_Date("KIA-53094 Aarupgaard U681", 2305, 20);

};

R_Combine("Aarupgaard U766")

{

R_Date("RICH-25343 Aarupgaard U766", 2317, 26);

R_Date("GrM-14589 Aarupgaard U766", 2285, 20);

R_Date("KIA-52821 Aarupgaard U766", 2252, 23);

};

R_Combine("Aarupgaard U1186")

{

R_Date("GrM-14597 Aarupgaard U1186", 2465, 20);

R_Date("KIA-53096 Aarupgaard U1186", 2408, 20);

};

R_Combine("Aarupgaard U51")

{

R_Date("KIA-52339 Aarupgaard U51", 2448, 26);

R_Date("GrM-15072 Aarupgaard U51", 2445, 20);

};

R_Combine("Aarupgaard U382")

{

R_Date("GrM-14596 Aarupgaard U382", 2280, 20);

R_Date("KIA-52827 Aarupgaard U382", 2229, 25);

};

R_Combine("Aarupgaard U1001")

{

R_Date("KIA-53095 Aarupgaard U1001", 2253, 21);

R_Date("GrM-14599 Aarupgaard U1001", 2235, 20);

};

R_Combine("Aarupgaard U1076")

{

R_Date("GrM-14592 Aarupgaard U1076", 2260, 20);

R_Date("KIA-52822 Aarupgaard U1076", 2199, 29);

R_Date("RICH-25340 Aarupgaard U1076", 2198, 27);

};

R_Combine("Aarupgaard U1363")

{

R_Date("GrM-14602 Aarupgaard U1363", 2225, 20);

R_Date("KIA-52828 Aarupgaard U1363", 2195, 24);

};

R_Combine("Aarupgaard U1791")

{

R_Date("GrM-15074 Aarupgaard U1791", 2230, 25);

R_Date("KIA-52341 Aarupgaard U1791", 2167, 25);

};

R_Combine("Aarupgaard U1847")

{

R_Date("GrM-15075 Aarupgaard U1847", 2255, 20);

R_Date("KIA-52342 Aarupgaard U1847", 2228, 25);

};

R_Combine("Aarupgaard U2262")

{

R_Date("GrM-14593 Aarupgaard U2262", 2255, 25);

R_Date("KIA-52823 Aarupgaard U2262", 2214, 28);

};

R_Combine("Søhale x76-II")

{

R_Date("KIA-53434 Søhale x76-II", 2434, 21);

R_Date("KIA-53940 Søhale x76-II", 2429, 26);

};

R_Combine("Søhale x44-II")

{

R_Date("KIA-53939 Søhale x44-II", 2468, 25);

R_Date("GrM-16772 Søhale x44-II", 2465, 20);

};

R_Combine("Søhale x12/x25A")

{

R_Date("AAR-25244 Søhale x12/x25A", 2181, 38);

R_Date("AAR-25245 Søhale x12/x25A", 2232, 34);

};

R_Combine("Søhale x38-II")

{

R_Date("RICH-26495 Søhale x38-II", 2254, 26);

R_Date("KIA-53938 Søhale x38-II", 2192, 27);

};

};

Outlier_Model("cremation", Exp(0.9,-10,-0.1), U(1,3),"t");

Sequence("BA per.V-VI")

{

Boundary("start BA per.V");

Phase("BA per.V")

{

R_Date("AAR-8786(PB4) Ø.Herup CB", 2722, 25)

{

Outlier("cremation", 1);

};

R_Date("AAR-8787 (PB5) Sevel Kalkværk CB", 2650, 25)

{

Outlier("cremation", 1);

};

R_Date("AAR-9520 (PB24) Jersild CB", 2683, 36)

{

Outlier("cremation", 1);

};

Combine("Gl. Brydegård")

{

R_Date("AAR-9570 (PB32) Gl. Brydegård pitch", 2706, 35);

R_Date("AAR-9576 (PB38) Gl. Brydegård CB", 2714, 34)

{

Outlier("cremation", 1);

};

};

R_Date("AAR-9575 (PB37) Lusehøj GX CB", 2611, 33)

{

Outlier("cremation", 1);

};

};

Boundary("Transition V/VI");

Phase("BA VI")

{

R_Date("AAR-9571 (PB33) Lerbjerg I CB", 2486, 25)

{

Outlier("cremation", 1);

};

R_Date("AAR-9568 (PB30) Lerbjerg I pitch", 2851, 24)

{

Outlier();

};

Combine("Lerbjerg II")

{

R_Date("AAR-9569 (PB31) Lerbjerg II pitch", 2459, 41);

R_Date("AAR-9573 (PB35) Lerbjerg II CB", 2502, 39)

{

Outlier("cremation", 1);

};

};

};

Boundary("end BA per.VI");

};

Page( );

Sequence("urnfields")

{

After("Bronze Age Period VI")

{

Date("=end BA per.VI");

};

Boundary("start urnfields");

Phase("urnfields")

{

Sequence ("Aarre urnfield")

{

Boundary("start Aarre");

Phase ("Aarre urnfield cemetery")

{

R_Date("RICH-25356 Aarre A89x311", 2464, 27)

{

Outlier("cremation", 1);

};

Date("=Aarre A281x484")

{

Outlier("cremation", 1);

};

Sequence ("A86")

{

After ("older CC from A86")

{

R_Date ("KIA-53941 Aarre A86x339 ", 2463, 25);

};

Phase("A86")

{

Date("=Aarre A86x340")

{

Outlier ("cremation", 1);

};

};

};

Combine("A95")

{

R_Date ("RICH-25342 Aarre A95x369 CB", 2428, 27)

{

Outlier ("cremation", 1);

};

Date ("=Aarre A95x368 twigs");

};

Sequence ("A99")

{

After ("older CC from A99")

{

R_Date ("RICH-25071 Aarre A99x65 no.2", 2269, 29);

R_Date ("RICH-25066 Aarre A99x346 no.27", 2251, 30);

R_Date ("RICH-25067 Aarre A99x65 no.3", 3115, 31);

};

Phase("A99")

{

R_Date ("GrM-16774 Aarre A99x345", 2255, 20)

{

Outlier ("cremation", 1);

};

R_Date ("RICH-25069 Aarre A99x346 no.1", 2085, 29)

{

Outlier();

};

};

};

Sequence ("A117")

{

After ("older CC from A117")

{

R_Date ("KIA-53943 Aarre A117x769", 2449, 25);

R_Date ("KIA-53944 A117x774", 2495, 24);

};

Phase("A117")

{

Date("=Aarre A117x762")

{

Outlier ("cremation", 1);

};

};

};

Sequence ("A130")

{

After ("older CC from A130")

{

R_Date ("KIA-53945 A130x82 no.1", 2585, 25);

};

Combine("A130")

{

R_Date ("KIA-53947 Aarre A130x217", 2253, 18)

{

Outlier ("cremation", 1);

};

R_Date ("KIA-53946 A130x82 no.2", 2156, 24);

};

};

Sequence ("A155")

{

After ("older CC from A155")

{

R_Date ("KIA-53948 A155x127 no.1", 2494, 24);

R_Date ("KIA-53949 A155x127 no.2", 2466, 24);

};

Phase("A155")

{

Date("=Aarre A155x281")

{

Outlier ("cremation", 1);

};

};

};

Sequence ("A198")

{

After ("older CC from A198")

{

R_Date ("KIA-53951 A198x338", 2967,24);

};

Phase("A198")

{

Date("=Aarre A198x338")

{

Outlier ("cremation", 1);

};

};

};

Combine("A278")

{

Date("=Aarre A278x783")

{

Outlier ("cremation", 1);

};

Date("=Aarre A278x782 grass");

};

Sequence ("A393")

{

After ("older CC from A393")

{

R_Date ("A393 RICH-25068", 2901, 32);

R_Date ("A393 RICH-25070", 2914, 32);

R_Date ("A393 KIA-52411", 3134, 25);

R_Date ("A393 KIA-52412", 3150, 27);

R_Date ("A393 KIA-52413", 2611, 27);

};

R_Date ("Aarre A393 RICH-25341", 2480, 27)

{

Outlier ("cremation", 1);

};

};

Sequence ("A394")

{

After ("older CC from A394")

{

R_Date ("A394 KIA-52414", 2778, 27);

R_Date ("A394 KIA-52415", 2843, 26);

R_Date ("A394 KIA-52416", 2772, 26);

R_Date ("A394 KIA-52417", 2719, 27);

R_Date ("A394 KIA-53983", 3029, 24);

};

Date("=Aarre A394x785")

{

Outlier ("cremation", 1);

};

};

};

Span("duration Aarre urnfield");

KDE_Plot("Aarre urnfield",);

Boundary("end Aarre");

};

Page( );

Sequence("Aarupgaard urnfield - entrance model")

{

Boundary("start Aarupgaard");

Phase("founding graves")

{

Date("=Aarupgaard U3869")

{

Outlier ("cremation", 1);

};

Date("=Aarupgaard U3330")

{

Outlier ("cremation", 1);

};

R_Date("RICH-25354 Aarupgaard U3341", 2477, 27)

{

Outlier ("cremation", 1);

};

};

Phase("multiple pathways")

{

Date("=Aarupgaard U83")

{

Outlier ("cremation", 1);

};

R_Date("Aarupgaard U34", 2346, 28)

{

Outlier ("cremation", 1);

};

R_Date("Aarupgaard U36", 2424, 30)

{

Outlier ("cremation", 1);

};

Date("=Aarupgaard U81")

{

Outlier ("cremation", 1);

};

R_Date("Aarupgaard U123", 2277, 26)

{

Outlier ("cremation", 1);

};

Phase("U230")

{

R_Date("GrM-14704 Aarupgaard U230", 2546, 19)

{

Outlier ();

};

R_Date("KIA-52826 Aarupgaard U230", 2362, 25)

{

Outlier ("cremation", 1);

};

};

Date("=Aarupgaard U280")

{

Outlier ("cremation", 1);

};

R_Date("Aarupgaard U293", 2378, 23)

{

Outlier ("cremation", 1);

};

R_Date("Aarupgaard U500", 2278, 23)

{

Outlier ("cremation", 1);

};

Date("=Aarupgaard U681")

{

Outlier ("cremation", 1);

};

R_Date("Aarupgaard U752", 2317, 26)

{

Outlier ("cremation", 1);

};

Date("=Aarupgaard U766")

{

Outlier ("cremation", 1);

};

R_Date("Aarupgaard U797", 2255, 28)

{

Outlier ("cremation", 1);

};

Date("=Aarupgaard U1186")

{

Outlier ("cremation", 1);

};

R_Date("Aarupgaard U1279", 2467, 26)

{

Outlier ("cremation", 1);

};

R_Date("Aarupgaard U3452", 2525, 25)

{

Outlier ("cremation", 1);

};

R_Date("Aarupgaard U3778", 2452, 25)

{

Outlier ("cremation", 1);

};

Date("=Aarupgaard U51")

{

Outlier ("cremation", 1);

};

R_Date("Aarupgaard U2710", 2411, 27)

{

Outlier ("cremation", 1);

};

R_Date("Aarupgaard U3822", 2433, 26)

{

Outlier ("cremation", 1);

};

R_Date("Aarupgaard U1232", 2443, 27)

{

Outlier ("cremation", 1);

};

};

Boundary("transition from multiple to 2 pathways")

{

Start("Start of transition from multiple to 2 pathways");

Transition("Period of transition from multiple to 2 pathways");

End("End of transition from multiple to 2 pathways");

};

Phase("2 pathways")

{

R_Date("Aarupgaard U183", 2243, 23)

{

Outlier ("cremation", 1);

};

R_Date("Aarupgaard U346", 2246, 24)

{

Outlier ("cremation", 1);

};

Date("=Aarupgaard U382")

{

Outlier ("cremation", 1);

};

R_Date("Aarupgaard U858", 2258, 23)

{

Outlier ("cremation", 1);

};

R_Date("Aarupgaard U928", 2297, 25)

{

Outlier ("cremation", 1);

};

Date("=Aarupgaard U1001")

{

Outlier ("cremation", 1);

};

R_Date("Aarupgaard U1016", 2220, 25)

{

Outlier ("cremation", 1);

};

Date("=Aarupgaard U1076")

{

Outlier ("cremation", 1);

};

Date("=Aarupgaard U1363")

{

Outlier ("cremation", 1);

};

R_Date("Aarupgaard U1382", 2201, 27)

{

Outlier ("cremation", 1);

};

R_Date("Aarupgaard U1422", 2186, 26)

{

Outlier ("cremation", 1);

};

R_Date("Aarupgaard U1436", 2252, 28)

{

Outlier ("cremation", 1);

};

R_Date("Aarupgaard U1617", 2163, 28)

{

Outlier ("cremation", 1);

};

R_Date("Aarupgaard U1678", 2243, 25)

{

Outlier ("cremation", 1);

};

Date("=Aarupgaard U1791")

{

Outlier ("cremation", 1);

};

Date("=Aarupgaard U1847")

{

Outlier ("cremation", 1);

};

R_Date("Aarupgaard U1970", 2246, 23)

{

Outlier ("cremation", 1);

};

R_Date("Aarupgaard U1997", 2146, 27)

{

Outlier ("cremation", 1);

};

Date("=Aarupgaard U2262")

{

Outlier ("cremation", 1);

};

R_Date("Aarupgaard U2541", 2216, 26)

{

Outlier ("cremation", 1);

};

R_Date("Aarupgaard U2550", 2291, 26)

{

Outlier ("cremation", 1);

};

R_Date("KIA-55388_Aarupgaard U427",2241,17)

{

Outlier("cremation", 1);

};

R_Date("KIA-55389_Aarupgaard U867",2219,12)

{

Outlier("cremation", 1);

};

R_Date("KIA-55390_Aarupgaard U871",2239,17)

{

Outlier("cremation", 1);

};

R_Date("KIA-55391_Aarupgaard U884",2211,16)

{

Outlier("cremation", 1);

};

R_Date("KIA-55392_Aarupgaard U1018",2242,17)

{

Outlier("cremation", 1);

};

R_Date("KIA-55393_Aarupgaard U1654",2183,14)

{

Outlier("cremation", 1);

};

Phase("U1834")

{

R_Date("RICH-24144 Aarupgaard U1834", 2322, 25)

{

Outlier();

};

R_Date("KIA-55394 Aarupgaard U1834", 2216, 18)

{

Outlier("cremation", 1);

};

};

R_Date("KIA-55395_Aarupgaard U1894",2211,14)

{

Outlier("cremation", 1);

};

R_Date("KIA-55396_Aarupgaard U1993",2228,15)

{

Outlier("cremation", 1);

};

R_Date("KIA-55397_Aarupgaard U2199",2244,17)

{

Outlier("cremation", 1);

};

R_Date("KIA-55398_Aarupgaard U2293",2185,25)

{

Outlier("cremation", 1);

};

R_Date("KIA-55399_Aarupgaard U2354",2237,18)

{

Outlier("cremation", 1);

};

R_Date("KIA-55400_Aarupgaard U2366",2232,15)

{

Outlier("cremation", 1);

};

R_Date("KIA-55401_Aarupgaard U2455",2240,25)

{

Outlier("cremation", 1);

};

R_Date("KIA-55402_Aarupgaard U2498",2165,25)

{

Outlier("cremation", 1);

};

R_Date("KIA-55403_Aarupgaard U2545",2194,18)

{

Outlier("cremation", 1);

};

R_Date("KIA-55404_Aarupgaard U2593",2222,17)

{

Outlier("cremation", 1);

};

};

Span("duration Aarupgaard urnfield");

KDE_Plot("Aarupgaard urnfield", );

Boundary("end Aarupgaard");

};

Page( );

Sequence("Søhale urnfield")

{

Boundary("start Søhale");

Phase("all burials")

{

R_Date("AAR-25258 Søhale x47-III", 2421, 27)

{

Outlier ("cremation", 1);

};

R_Date("RICH-26502 Søhale x34-II", 2342, 26)

{

Outlier ("cremation", 1);

};

R_Date("AAR-25256 Søhale x33-II", 2353, 27)

{

Outlier ("cremation", 1);

};

R_Date("AAR-25262 Søhale x55-II", 2345, 27)

{

Outlier ("cremation", 1);

};

Phase("no circular ditch")

{

Date("=Søhale x76-II")

{

Outlier("cremation", 1);

};

R_Date("KIA-53432 Søhale x72-VII", 2382, 21)

{

Outlier ("cremation", 1);

};

R_Date("KIA-53433 Søhale x74-II", 2373, 21)

{

Outlier ("cremation", 1);

};

};

Sequence("no entrances")

{

Boundary("start no entrances");

Phase("no entrances")

{

R_Date("AAR-25257 Søhale x35-II", 2469, 26)

{

Outlier ("cremation", 1);

};

Date("=Søhale x44-II")

{

Outlier("cremation", 1);

};

R_Date("GrM-16773 Søhale x49-III", 2425, 19)

{

Outlier ("cremation", 1);

};

R_Date("KIA-53431 Søhale x50-II", 2438, 21)

{

Outlier ("cremation", 1);

};

R_Date("AAR-25260 Søhale x51-IV", 2440, 27)

{

Outlier ("cremation", 1);

};

R_Date("AAR-25261 Søhale x52-II", 2418, 31)

{

Outlier ("cremation", 1);

};

R_Date("KIA-53435 Søhale x92-III", 2427, 21)

{

Outlier ("cremation", 1);

};

};

Boundary("end no entrances");

};

Sequence("N-S entrances")

{

Boundary("start N-S entrances");

Phase("N-S entrances")

{

R_Date("AAR-25252 Søhale x26-III", 2277, 38)

{

Outlier ("cremation", 1);

};

R_Date("AAR-25259 Søhale x48-II", 2460, 30)

{

Outlier ("cremation", 1);

};

R_Date("AAR-25250 Søhale x21-III", 2258, 27)

{

Outlier ("cremation", 1);

};

R_Date("AAR-25265 Søhale x93-II", 2387, 29)

{

Outlier ("cremation", 1);

};

R_Date("AAR-25263 Søhale x65-V", 2314, 28)

{

Outlier ("cremation", 1);

};

R_Date("AAR-25249 Søhale x18-III", 2185, 27)

{

Outlier ("cremation", 1);

};

};

Boundary("end N-S entrances");

};

Sequence("NNE-SSW entrances")

{

Boundary("start NNE-SSW entrances");

Phase("NNE-SSW entrances")

{

R_Date("AAR-25251 Søhale x22-III", 2211, 27)

{

Outlier ("cremation", 1);

};

R_Date("AAR-25255 Søhale x32-II", 2403, 29)

{

Outlier ("cremation", 1);

};

R_Date("AAR-25246 Søhale x14", 2339, 26)

{

Outlier ("cremation", 1);

};

R_Date("RICH-26501 Søhale x23-II", 2314, 24)

{

Outlier ("cremation", 1);

};

R_Date("AAR-25254 Søhale x30-III", 2322, 28)

{

Outlier ("cremation", 1);

};

R_Date("RICH-26494 Søhale x31-II", 2337, 27)

{

Outlier ("cremation", 1);

};

R_Date("GrM-16771 Søhale x41-V", 2370, 20)

{

Outlier ("cremation", 1);

};

R_Date("AAR-25264 Søhale x69-IV", 2303, 28)

{

Outlier ("cremation", 1);

};

R_Date("AAR-25243 Søhale x10-II", 2212, 30)

{

Outlier ("cremation", 1);

};

Date("=Søhale x12/x25A")

{

Outlier("cremation", 1);

};

R_Date("AAR-25248 Søhale x17", 2262, 40)

{

Outlier ("cremation", 1);

};

R_Date("KIA-53936 Søhale x19-II", 2265, 26)

{

Outlier ("cremation", 1);

};

R_Date("AAR-25253 Søhale x27-I",2237, 30)

{

Outlier ("cremation", 1);

};

R_Date("RICH-26493 Søhale x28-II", 2245, 27)

{

Outlier ("cremation", 1);

};

R_Date("KIA-53937 Søhale x37-III", 2220, 20)

{

Outlier ("cremation", 1);

};

Date("=Søhale x38-II")

{

Outlier("cremation", 1);

};

R_Date("GrM-16770 Søhale x40-II", 2227, 19)

{

Outlier ("cremation", 1);

};

};

Boundary("end NNE-SSW entrances");

};

};

Span ("Duration Søhale urnfield cemetery");

KDE_Plot("Søhale urnfield", );

Boundary("end Søhale");

};

};

KDE_Plot("urnfield burial activity", );

Span("duration of urnfields");

Boundary("end urnfields");

};

};

# S2.3. Alternative site model C

To run the alternative horizontal model of Aarupgaard urnfield, replace the Sequence "Aarupgaard urnfield" in urnfield model B with the model code given below.

Sequence("Aarupgaard urnfield")

{

Boundary("start Aarupgaard");

Phase("founding graves")

{

R_Date("Aarupgaard grave 3869 CB", 2507, 14)

{

Outlier ("cremation", 1);

};

R_Date("Aarupgaard grave 3330 CB comb", 2509, 14)

{

Outlier ("cremation", 1);

};

R_Date("Aarupgaard grave 3340 CB", 2477, 27)

{

Outlier ("cremation", 1);

};

};

Phase("burial groups M1-M2")

{

Phase("burial group M1")

{

Sequence("M1-CC1")

{

Boundary("start M1-CC1");

Phase("M1-CC1")

{

R_Date("Aarupgaard grave 3452 CB", 2525, 25)

{

Outlier ("cremation", 1);

};

R_Date("Aarupgaard grave 2710 CB", 2411, 27)

{

Outlier ("cremation", 1);

};

R_Date("Aarupgaard grave 1279 CB", 2467, 26)

{

Outlier ("cremation", 1);

};

R_Date("Aarupgaard grave 3778 CB", 2452, 25)

{

Outlier ("cremation", 1);

};

R_Date("Aarupgaard grave 81 CB comb", 2395, 18)

{

Outlier ("cremation", 1);

};

R_Date("Aarupgaard grave 1232 CB", 2443, 27)

{

Outlier ("cremation", 1);

};

R_Date("Aarupgaard grave 51 CB comb", 2446, 16)

{

Outlier ("cremation", 1);

};

R_Date("Aarupgaard grave 83 CB comb", 2443, 21)

{

Outlier ("cremation", 1);

};

R_Date("Aarupgaard grave 1186 CB comb", 2437, 15)

{

Outlier ("cremation", 1);

};

Sum("M1-CC1");

};

Boundary("end M1-CC1");

};

Sequence("M1-CC2")

{

Boundary("start M1-CC2");

Phase("M1-CC2")

{

R_Date("Aarupgaard grave 123 CB", 2277, 26)

{

Outlier ("cremation", 1);

};

R_Date("Aarupgaard grave 280 CB comb", 2404, 15)

{

Outlier ("cremation", 1);

};

R_Date("Aarupgaard grave 36 CB", 2424, 30)

{

Outlier ("cremation", 1);

};

R_Date("Aarupgaard grave 34 CB", 2346, 28)

{

Outlier ("cremation", 1);

};

R_Date("Aarupgaard grave 230 CB", 2362, 25)

{

Outlier ("cremation", 1);

};

R_Date("Aarupgaard grave 293 CB", 2378, 23)

{

Outlier ("cremation", 1);

};

Sum("M1-CC2");

};

Boundary("end M1-CC2");

};

Sequence("M1-CC3")

{

Boundary("start M1-CC3");

Phase("M1-CC3")

{

R_Date("Aarupgaard grave 500 CB", 2278, 23)

{

Outlier ("cremation", 1);

};

R_Date("Aarupgaard grave 681 CB comb", 2308, 14)

{

Outlier ("cremation", 1);

};

R_Date("Aarupgaard grave 752 CB", 2317, 26)

{

Outlier ("cremation", 1);

};

R_Date("Aarupgaard grave 766 CB comb", 2271, 16)

{

Outlier ("cremation", 1);

};

R_Date("Aarupgaard grave 928 CB", 2297, 25)

{

Outlier ("cremation", 1);

};

R_Date("Aarupgaard grave 797 CB", 2255, 28)

{

Outlier ("cremation", 1);

};

Sum("M1-CC3");

};

Boundary("end M1-CC3");

};

Sequence("M1-CC4")

{

Boundary("start M1-CC4");

Phase("M1-CC4")

{

R_Date("Aarupgaard grave 1363 CB comb", 2213, 16)

{

Outlier ("cremation", 1);

};

R_Date("Aarupgaard grave 1382 CB", 2201, 27)

{

Outlier ("cremation", 1);

};

R_Date("Aarupgaard grave 1076 CB comb", 2229, 15)

{

Outlier ("cremation", 1);

};

R_Date("Aarupgaard grave 1422 CB", 2186, 26)

{

Outlier ("cremation", 1);

};

R_Date("Aarupgaard grave 1436 CB", 2252, 28)

{

Outlier ("cremation", 1);

};

R_Date("Aarupgaard grave 1001 CB comb", 2244, 15)

{

Outlier ("cremation", 1);

};

Sum("M1-CC4");

};

Boundary("end M1-CC4");

};

Sequence("M1-CC5")

{

Boundary("start M1-CC5");

Phase("M1-CC5")

{

R_Date("Aarupgaard grave 1617 CB", 2163, 28)

{

Outlier ("cremation", 1);

};

R_Date("KIA-55393_Aarupgaard grave 1654",2183,14)

{

Outlier("cremation", 1);

};

R_Date("Aarupgaard grave 1678 CB", 2243, 25)

{

Outlier ("cremation", 1);

};

R_Date("Aarupgaard grave 1791 CB comb", 2199, 18)

{

Outlier ("cremation", 1);

};

Combine("Aarupgaard grave 1834")

{

R_Date("KIA-55394_Aarupgaard grave 1834",2216,18)

{

Outlier("cremation", 1);

};

R_Date("RICH-24144_Aarupgaard grave 1834", 2322, 25)

{

Outlier ();

};

};

R_Date("Aarupgaard grave 1847 CB comb", 2244, 16)

{

Outlier ("cremation", 1);

};

R_Date("KIA-55395_Aarupgaard grave 1894",2211,14)

{

Outlier("cremation", 1);

};

R_Date("Aarupgaard grave 1970 CB", 2246, 23)

{

Outlier ("cremation", 1);

};

R_Date("KIA-55396_Aarupgaard grave 1993",2228,15)

{

Outlier("cremation", 1);

};

R_Date("Aarupgaard grave 1997 CB", 2146, 27)

{

Outlier ("cremation", 1);

};

Sum("M1-CC5");

};

Boundary("end M1-CC5");

};

Sequence("M1-CC6")

{

Boundary("start M1-CC6");

Phase("M1-CC6")

{

R_Date("KIA-55397_Aarupgaard grave 2199",2244,17)

{

Outlier("cremation", 1);

};

R_Date("Aarupgaard grave 2541 CB", 2216, 26)

{

Outlier ("cremation", 1);

};

R_Date("Aarupgaard grave 2262 CB comb", 2237, 19)

{

Outlier ("cremation", 1);

};

R_Date("Aarupgaard grave 2550 CB", 2291, 26)

{

Outlier ("cremation", 1);

};

R_Date("KIA-55398_Aarupgaard grave 2293",2185,25)

{

Outlier("cremation", 1);

};

R_Date("KIA-55399_Aarupgaard grave 2354",2237,18)

{

Outlier("cremation", 1);

};

R_Date("KIA-55400_Aarupgaard grave 2366",2232,15)

{

Outlier("cremation", 1);

};

R_Date("KIA-55401_Aarupgaard grave 2455",2240,25)

{

Outlier("cremation", 1);

};

R_Date("KIA-55402_Aarupgaard grave 2498",2165,25)

{

Outlier("cremation", 1);

};

R_Date("KIA-55403_Aarupgaard grave 2545",2194,18)

{

Outlier("cremation", 1);

};

R_Date("KIA-55404_Aarupgaard grave 2593",2222,17)

{

Outlier("cremation", 1);

};

Sum("M1-CC6");

};

Boundary("end M1-CC6");

};

};

Phase("burial group M2")

{

Sequence("M2-CC1-3")

{

Boundary("start M2-CC1-3");

Phase("M2-CC1-3")

{

R_Date("Aarupgaard grave 3822 CB", 2433, 26)

{

Outlier ("cremation", 1);

};

R_Date("Aarupgaard grave 346 CB", 2246, 24)

{

Outlier ("cremation", 1);

};

R_Date("Aarupgaard grave 183 CB", 2243, 23)

{

Outlier ("cremation", 1);

};

R_Date("Aarupgaard grave 382 CB comb", 2260, 16)

{

Outlier ("cremation", 1);

};

R_Date("KIA-55388_Aarupgaard grave 427",2241,17)

{

Outlier("cremation", 1);

};

Sum("M2-CC1-3");

};

Boundary("end M2-CC1-3");

};

Sequence("M2-CC4")

{

Boundary("start M2-CC4");

Phase("M2-CC4")

{

R_Date("Aarupgaard grave 858 CB", 2258, 23)

{

Outlier ("cremation", 1);

};

R_Date("KIA-55389_Aarupgaard grave 867",2219,12)

{

Outlier("cremation", 1);

};

R_Date("KIA-55390_Aarupgaard grave 871",2239,17)

{

Outlier("cremation", 1);

};

R_Date("KIA-55391_Aarupgaard grave 884",2211,16)

{

Outlier("cremation", 1);

};

R_Date("Aarupgaard grave 1016 CB", 2220, 25)

{

Outlier ("cremation", 1);

};

R_Date("KIA-55392_Aarupgaard grave 1018",2242,17)

{

Outlier("cremation", 1);

};

Sum("M2-CC4");

};

Boundary("end M2-CC4");

};

};

};

Span("duration Aarupgaard urnfield");

KDE_Plot("Aarupgaard urnfield", );

Boundary("end Aarupgaard");

};

Phase("Aarupgaard - horizontal stratigraphy")

{

Phase("M1 - order of spatial phases")

{

Sequence("M1 - start of phases")

{

Date("=start M1-CC1");

Date("=start M1-CC2");

Date("=start M1-CC3");

Date("=start M1-CC4");

Date("=start M1-CC5");

Date("=start M1-CC6");

};

};

Phase("M2 - order of spatial phases")

{

Sequence("M2 - start of phases")

{

Date("=start M2-CC1-3");

Date("=start M2-CC4");

};

};

};

# S2.4. Alternative site model D

To run the alternative chronological model of Søhale urnfield based on the orientation of interruptions of circular ditches, replace the Sequence "Søhale urnfield" in urnfield model B with the model code given below.

Sequence("Søhale urnfield")

{

Boundary("start Søhale");

Phase("all burials")

{

R_Date("Søhale x47-III AAR-25258", 2421, 27)

{

Outlier ("cremation", 1);

};

R_Date("Søhale x34-II RICH-26502", 2342, 26)

{

Outlier ("cremation", 1);

};

R_Date("Søhale x33-II AAR-25256", 2353, 27)

{

Outlier ("cremation", 1);

};

R_Date("Søhale x55-II AAR-25262", 2345, 27)

{

Outlier ("cremation", 1);

};

Phase("no circular ditch")

{

Combine("Søhale x76-II comb")

{

R_Date("KIA-53434", 2434, 21)

{

Outlier("cremation", 1);

};

R_Date("KIA-53940", 2429, 26)

{

Outlier("cremation", 1);

};

};

R_Date("Søhale x72-VII KIA-53432", 2382, 21)

{

Outlier ("cremation", 1);

};

R_Date("Søhale x74-II KIA-53433", 2373, 21)

{

Outlier ("cremation", 1);

};

};

Sequence("no entrances")

{

Boundary("start no entrances");

Phase("no entrances")

{

R_Date("Søhale x35-II AAR-25257", 2469, 26)

{

Outlier ("cremation", 1);

};

Combine("Søhale x44-II comb")

{

R_Date("KIA-53939", 2468, 25)

{

Outlier("cremation", 1);

};

R_Date("GrM-16772", 2465, 20)

{

Outlier("cremation", 1);

};

};

R_Date("Søhale x49-III GrM-16773", 2425, 19)

{

Outlier ("cremation", 1);

};

R_Date("Søhale x50-II KIA-53431", 2438, 21)

{

Outlier ("cremation", 1);

};

R_Date("Søhale x51-IV AAR-25260", 2440, 27)

{

Outlier ("cremation", 1);

};

R_Date("Søhale x52-II AAR-25261", 2418, 31)

{

Outlier ("cremation", 1);

};

R_Date("Søhale x92-III KIA-53435", 2427, 21)

{

Outlier ("cremation", 1);

};

};

Boundary("end no entrances");

};

Sequence("N-S entrances")

{

Boundary("start N-S entrances");

Phase("N-S entrances")

{

R_Date("Søhale x26-III AAR-25252", 2277, 38)

{

Outlier ("cremation", 1);

};

R_Date("Søhale x48-II AAR-25259", 2460, 30)

{

Outlier ("cremation", 1);

};

R_Date("Søhale x21-III AAR-25250", 2258, 27)

{

Outlier ("cremation", 1);

};

R_Date("Søhale x93-II AAR-25265", 2387, 29)

{

Outlier ("cremation", 1);

};

R_Date("Søhale x65-V AAR-25263", 2314, 28)

{

Outlier ("cremation", 1);

};

R_Date("Søhale x18-III AAR-25249", 2185, 27)

{

Outlier ("cremation", 1);

};

};

Boundary("end N-S entrances");

};

Sequence("NNE-SSW entrances")

{

Boundary("start NNE-SSW entrances");

Phase("NNE-SSW entrances")

{

R_Date("Søhale x22-III AAR-25251", 2211, 27)

{

Outlier ("cremation", 1);

};

R_Date("Søhale x32-II AAR-25255", 2403, 29)

{

Outlier ("cremation", 1);

};

R_Date("Søhale x14 AAR-25246", 2339, 26)

{

Outlier ("cremation", 1);

};

R_Date("Søhale x23-II RICH-26501", 2314, 24)

{

Outlier ("cremation", 1);

};

R_Date("Søhale x30-III AAR-25254", 2322, 28)

{

Outlier ("cremation", 1);

};

R_Date("Søhale x31-II RICH-26494", 2337, 27)

{

Outlier ("cremation", 1);

};

R_Date("Søhale x41-V GrM-16771", 2370, 20)

{

Outlier ("cremation", 1);

};

R_Date("Søhale x69-IV AAR-25264", 2303, 28)

{

Outlier ("cremation", 1);

};

R_Date("Søhale x10-II AAR-25243", 2212, 30)

{

Outlier ("cremation", 1);

};

Combine("Søhale x12/x25A comb")

{

R_Date("AAR-25244", 2181, 38)

{

Outlier("cremation", 1);

};

R_Date("AAR-25245", 2232, 34)

{

Outlier("cremation", 1);

};

};

R_Date("Søhale x17 AAR-25248", 2262, 40)

{

Outlier ("cremation", 1);

};

R_Date("Søhale x19-II KIA-53936", 2265, 26)

{

Outlier ("cremation", 1);

};

R_Date("Søhale x27-I AAR-25253",2237, 30)

{

Outlier ("cremation", 1);

};

R_Date("Søhale x28-II RICH-26493", 2245, 27)

{

Outlier ("cremation", 1);

};

R_Date("Søhale x37-III KIA-53937", 2220, 20)

{

Outlier ("cremation", 1);

};

Combine("Søhale x38-II comb")

{

R_Date("RICH-26495", 2254, 26)

{

Outlier("cremation", 1);

};

R_Date("KIA-53938", 2192, 27)

{

Outlier("cremation", 1);

};

};

R_Date("Søhale x40-II GrM-16770", 2227, 19)

{

Outlier ("cremation", 1);

};

};

Boundary("end NNE-SSW entrances");

};

};

Span ("Duration Søhale urnfield cemetery");

KDE_Plot("Søhale urnfield", );

Boundary("end Søhale");

};

# S2.5. Currency model

Run the urnfield model B first and save the posterior estimated burial dates using the exact same names as provided below in Phase("burial dates").

Plot()

{

MCMC_Sample()

{

};

Phase("burial dates")

{

Phase("Aarupgaard burials")

{

Prior("Aarupgaard U34_burial", "Aarupgaard_U34.prior");

Prior("Aarupgaard U36_burial", "Aarupgaard_U36.prior");

Prior("Aarupgaard U51_burial", "Aarupgaard_U51.prior");

Prior("Aarupgaard U81_burial", "Aarupgaard_U81.prior");

Prior("Aarupgaard U83_burial", "Aarupgaard_U83.prior");

Prior("Aarupgaard U123_burial", "Aarupgaard_U123.prior");

Prior("Aarupgaard U183_burial", "Aarupgaard_U183.prior");

Prior("Aarupgaard U230_burial", "Aarupgaard_U230.prior");

Prior("Aarupgaard U280_burial", "Aarupgaard_U280.prior");

Prior("Aarupgaard U293_burial", "Aarupgaard_U293.prior");

Prior("Aarupgaard U346_burial", "Aarupgaard_U346.prior");

Prior("Aarupgaard U382_burial", "Aarupgaard_U382.prior");

Prior("Aarupgaard U427_burial", "Aarupgaard_U427.prior");

Prior("Aarupgaard U500_burial", "Aarupgaard_U500.prior");

Prior("Aarupgaard U681_burial", "Aarupgaard_U681.prior");

Prior("Aarupgaard U752_burial", "Aarupgaard_U752.prior");

Prior("Aarupgaard U766_burial", "Aarupgaard_U766.prior");

Prior("Aarupgaard U797_burial", "Aarupgaard_U797.prior");

Prior("Aarupgaard U858_burial", "Aarupgaard_U858.prior");

Prior("Aarupgaard U867_burial", "Aarupgaard_U867.prior");

Prior("Aarupgaard U871_burial", "Aarupgaard_U871.prior");

Prior("Aarupgaard U884_burial", "Aarupgaard_U884.prior");

Prior("Aarupgaard U928_burial", "Aarupgaard_U928.prior");

Prior("Aarupgaard U1001_burial", "Aarupgaard_U1001.prior");

Prior("Aarupgaard U1016_burial", "Aarupgaard_U1016.prior");

Prior("Aarupgaard U1018_burial", "Aarupgaard_U1018.prior");

Prior("Aarupgaard U1076_burial", "Aarupgaard_U1076.prior");

Prior("Aarupgaard U1186_burial", "Aarupgaard_U1186.prior");

Prior("Aarupgaard U1232_burial", "Aarupgaard_U1232.prior");

Prior("Aarupgaard U1279_burial", "Aarupgaard_U1279.prior");

Prior("Aarupgaard U1363_burial", "Aarupgaard_U1363.prior");

Prior("Aarupgaard U1382_burial", "Aarupgaard_U1382.prior");

Prior("Aarupgaard U1422_burial", "Aarupgaard_U1422.prior");

Prior("Aarupgaard U1436_burial", "Aarupgaard_U1436.prior");

Prior("Aarupgaard U1617_burial", "Aarupgaard_U1617.prior");

Prior("Aarupgaard U1654_burial", "Aarupgaard_U1654.prior");

Prior("Aarupgaard U1678_burial", "Aarupgaard_U1678.prior");

Prior("Aarupgaard U1791_burial", "Aarupgaard_U1791.prior");

Prior("Aarupgaard U1834_burial", "Aarupgaard_U1834.prior");

Prior("Aarupgaard U1847_burial", "Aarupgaard_U1847.prior");

Prior("Aarupgaard U1894_burial", "Aarupgaard_U1894.prior");

Prior("Aarupgaard U1970_burial", "Aarupgaard_U1970.prior");

Prior("Aarupgaard U1993_burial", "Aarupgaard_U1993.prior");

Prior("Aarupgaard U1997_burial", "Aarupgaard_U1997.prior");

Prior("Aarupgaard U2199_burial", "Aarupgaard_U2199.prior");

Prior("Aarupgaard U2262_burial", "Aarupgaard_U2262.prior");

Prior("Aarupgaard U2293_burial", "Aarupgaard_U2293.prior");

Prior("Aarupgaard U2354_burial", "Aarupgaard_U2354.prior");

Prior("Aarupgaard U2366_burial", "Aarupgaard_U2366.prior");

Prior("Aarupgaard U2455_burial", "Aarupgaard_U2455.prior");

Prior("Aarupgaard U2498_burial", "Aarupgaard_U2498.prior");

Prior("Aarupgaard U2541_burial", "Aarupgaard_U2541.prior");

Prior("Aarupgaard U2545_burial", "Aarupgaard_U2545.prior");

Prior("Aarupgaard U2550_burial", "Aarupgaard_U2550.prior");

Prior("Aarupgaard U2593_burial", "Aarupgaard_U2593.prior");

Prior("Aarupgaard U2710_burial", "Aarupgaard_U2710.prior");

Prior("Aarupgaard U3452_burial", "Aarupgaard_U3452.prior");

Prior("Aarupgaard U3778_burial", "Aarupgaard_U3778.prior");

Prior("Aarupgaard U3822_burial", "Aarupgaard_U3822.prior");

};

Phase("Søhale burials")

{

Prior("Søhale x18_burial", "Sohale_x18.prior");

Prior("Søhale x19_burial", "Sohale_x19.prior");

Prior("Søhale x23_burial", "Sohale_x23.prior");

Prior("Søhale x26_burial", "Sohale_x26.prior");

Prior("Søhale x28_burial", "Sohale_x28.prior");

Prior("Søhale x30_burial", "Sohale_x30.prior");

Prior("Søhale x31_burial", "Sohale_x31.prior" );

Prior("Søhale x32_burial", "Sohale_x32.prior");

Prior("Søhale x34_burial", "Sohale_x34.prior");

Prior("Søhale x37_burial", "Sohale_x37.prior");

Prior("Søhale x38_burial", "Sohale_x38.prior");

Prior("Søhale x40_burial", "Sohale_x40.prior");

Prior("Søhale x41_burial", "Sohale_x41.prior");

Prior("Søhale x44_burial", "Sohale_x44.prior");

Prior("Søhale x47_burial", "Sohale_x47.prior");

Prior("Søhale x49_burial", "Sohale_x49.prior");

Prior("Søhale x50_burial", "Sohale_x50.prior");

Prior("Søhale x51_burial", "Sohale_x51.prior");

Prior("Søhale x52_burial", "Sohale_x52.prior");

Prior("Søhale x65_burial", "Sohale_x65.prior");

Prior("Søhale x69_burial", "Sohale_x69.prior");

Prior("Søhale x72_burial", "Sohale_x72.prior");

Prior("Søhale x74_burial", "Sohale_x74.prior");

Prior("Søhale x76_burial", "Sohale_x76.prior");

Prior("Søhale x92_burial", "Sohale_x92.prior");

Prior("Søhale x93_burial", "Sohale_x93.prior");

};

Phase("Aarre burials")

{

Prior( "Aarre A86_burial", "Aarre_A86.prior");

Prior("Aarre A89_burial", "Aarre_A89.prior");

Prior("Aarre A99_burial", "Aarre_A99.prior");

Prior("Aarre A130_burial", "Aarre_A130.prior");

Prior("Aarre A155_burial", "Aarre_A155.prior");

Prior("Aarre A198_burial", "Aarre_A198.prior");

Prior("Aarre A278_burial", "Aarre_A278.prior");

Prior("Aarre A281_burial", "Aarre_A281.prior");

Prior("Aarre A393_burial", "Aarre_A393.prior");

Prior("Aarre A394_burial", "Aarre_A394.prior");

};

};

Sequence("pottery currencies")

{

After("Bronze Age Period VI")

{

Date("=end BA per.VI");

};

Boundary("start pottery currencies");

Phase("pottery")

{

Phase("pottery type 11A")

{

Date("Aarupgaard U2710_11A", Prior("Aarupgaard U2710", "Aarupgaard_U2710.prior"));

};

Phase("pottery type 13A")

{

Date("Aarupgaard U1186_13A", Prior("Aarupgaard U1186", "Aarupgaard_U1186.prior"));

Date("Aarupgaard U1279_13A", Prior("Aarupgaard U1279", "Aarupgaard_U1279.prior"));

};

Phase("pottery type 11B")

{

Date("Aarupgaard U3822_11B", Prior("Aarupgaard U3822", "Aarupgaard_U3822.prior"));

};

Sequence("pottery type 12B")

{

Boundary("start type 12B");

Phase("pottery type 12B")

{

Date("Aarupgaard U81_12B", Prior("Aarupgaard_U81", "Aarupgaard_U81.prior"));

Date("Aarupgaard U83_12B", Prior("Aarupgaard_U83", "Aarupgaard_U83.prior"));

Date("Aarupgaard U183_12B", Prior("Aarupgaard_U183", "Aarupgaard_U183.prior"));

Date("Aarupgaard U230_12B", Prior("Aarupgaard_U230", "Aarupgaard_U230.prior"));

Date("Aarupgaard U500_12B", Prior("Aarupgaard_U500", "Aarupgaard_U500.prior"));

Date("Aarupgaard U752_12B", Prior("Aarupgaard_U752", "Aarupgaard_U752.prior"));

Date("Aarupgaard U1834_12B", Prior("Aarupgaard_U1834", "Aarupgaard_U1834.prior"));

Date("Aarupgaard U3778_12B", Prior("Aarupgaard_U3778", "Aarupgaard_U3778.prior"));

};

Span("duration type 12B");

Sum("type 12B (n=8)");

Boundary("end type 12B");

};

Sequence("pottery type 15B")

{

Boundary("increase type 15B")

{

End("start of increase type 15B");

Transition("period of increase type 15B");

Start("end of increase type 15B");

};

Phase("pottery type 15B")

{

Date("Aarupgaard U51_15B", Prior("Aarupgaard U51", "Aarupgaard_U51.prior"));

Date("Aarupgaard U1232_15B", Prior("Aarupgaard U1232", "Aarupgaard_U1232.prior"));

Date("Aarupgaard U3452_15B", Prior("Aarupgaard U3452", "Aarupgaard_U3452.prior"));

Date("Aarupgaard U280_15B", Prior("Aarupgaard U280", "Aarupgaard_U280.prior"));

Date("Aarupgaard U928_15B", Prior("Aarupgaard U928", "Aarupgaard_U928.prior"));

Date("Aarupgaard U766_15B", Prior("Aarupgaard U766", "Aarupgaard_U766.prior"));

Date("Aarupgaard U858_15B", Prior("Aarupgaard U858", "Aarupgaard_U858.prior"));

Date("Aarupgaard U1363_15B", Prior("Aarupgaard U1363", "Aarupgaard_U1363.prior"));

Date("Aarupgaard U1016_15B", Prior("Aarupgaard U1016", "Aarupgaard_U1016.prior"));

Date("Aarupgaard U1001_15B", Prior("Aarupgaard U1001", "Aarupgaard_U1001.prior"));

Date("Aarupgaard U1018_15B", Prior("Aarupgaard U1018", "Aarupgaard_U1018.prior"));

};

Span("duration type 15B");

Sum("type 15B (n=11)");

Boundary("decrease type 15B")

{

Start("start of decrease type 15B");

Transition("period of decrease type 15B");

End("end of decrease type 15B");

};

};

Sequence("pottery type 20B")

{

Boundary("start type 20B");

Phase("pottery type 20B")

{

Date("Aarupgaard U34_20B", Prior("Aarupgaard U34", "Aarupgaard_U34.prior"));

Date("Aarupgaard U36_20B", Prior("Aarupgaard U36", "Aarupgaard_U36.prior"));

Date("Aarupgaard U293_20B", Prior("Aarupgaard U293", "Aarupgaard_U293.prior"));

Date("Aarupgaard U123_20B", Prior("Aarupgaard U123", "Aarupgaard_U123.prior"));

Date("Aarupgaard U2366_20B", Prior("Aarupgaard U2366", "Aarupgaard_U2366.prior"))-N(50,25);

};

Span("duration type 20B");

Sum("type 20B (n=5)");

Boundary("end type 20B");

};

Phase("type 13C (n=1)")

{

Date("Aarupgaard U797_13C", Prior("Aarupgaard U797", "Aarupgaard_U797.prior"));

};

Sequence("pottery type 15C")

{

Boundary("start type 15C");

Phase("pottery type 15C")

{

Date("Aarupgaard U1970_15C", Prior("Aarupgaard U1970", "Aarupgaard_U1970.prior"));

Date("Aarupgaard U346_15C", Prior("Aarupgaard U346", "Aarupgaard_U346.prior"));

Date("Aarupgaard U1678_15C", Prior("Aarupgaard U1678", "Aarupgaard_U1678.prior"));

Date("Aarupgaard U1436_15C", Prior("Aarupgaard U1436", "Aarupgaard_U1436.prior"));

Date("Aarupgaard U382_15C", Prior("Aarupgaard U382", "Aarupgaard_U382.prior"));

Date("Aarupgaard U1791_15C", Prior("Aarupgaard U1791", "Aarupgaard_U1791.prior"));

Date("Aarupgaard U1847_15C", Prior("Aarupgaard U1847", "Aarupgaard_U1847.prior"));

Date("Aarupgaard U2354_15C", Prior("Aarupgaard U2354", "Aarupgaard_U2354.prior"));

};

Span("duration type 15C");

Sum("type 15C (n=8)");

Boundary("end type 15C");

};

Sequence("pottery type 18C")

{

Boundary("start type 18C");

Phase("pottery type 18C")

{

Date("Aarupgaard U1997_18C", Prior("Aarupgaard U1997", "Aarupgaard_U1997.prior"));

Date("Aarupgaard U1382_18C", Prior("Aarupgaard U1382", "Aarupgaard_U1382.prior"));

Date("Aarupgaard U1617_18C", Prior("Aarupgaard U1617", "Aarupgaard_U1617.prior"));

Date("Aarupgaard U1076_18C", Prior("Aarupgaard U1076", "Aarupgaard_U1076.prior"));

Date("Aarupgaard U1001_18C", Prior("Aarupgaard U1001", "Aarupgaard_U1001.prior"));

};

Span("duration type 18C");

Sum("type 18C (n=5)");

Boundary("end type 18C");

};

Phase("type 11D (n=1)")

{

Date("Aarupgaard U2262_11D", Prior("Aarupgaard U2262", "Aarupgaard_U2262.prior"));

};

Phase("type 13D (n=2)")

{

Date("Aarupgaard U681_13D", Prior("Aarupgaard U681", "Aarupgaard_U681.prior"));

Date("Aarupgaard U1422_13D", Prior("Aarupgaard U1422", "Aarupgaard_U1422.prior"));

};

Sequence("pottery type 15D")

{

Boundary("start type 15D");

Phase("type 15D")

{

Date("Aarupgaard U2550_15D", Prior("Aarupgaard U2550", "Aarupgaard_U2550.prior"));

Date("Aarupgaard U867_15D", Prior("Aarupgaard U867", "Aarupgaard_U867.prior"));

Date("Aarupgaard U884_15D", Prior("Aarupgaard U884", "Aarupgaard_U884.prior"));

Date("Aarupgaard U2293_15D", Prior("Aarupgaard U2293", "Aarupgaard_U2293.prior"));

Date("Aarupgaard U2498_15D", Prior("Aarupgaard U2498", "Aarupgaard_U2498.prior"));

};

Span("duration type 15D");

Sum("type 15D (n=5)");

Boundary("end type 15D");

};

Phase("type 17D (n=1)")

{

Date("Aarupgaard U2593_17D", Prior("Aarupgaard U2593", "Aarupgaard_U2593.prior"));

};

Phase("type 20D (n=1)")

{

Date("Aarupgaard U2541_20D", Prior("Aarupgaard U2541", "Aarupgaard_U2541.prior"));

};

};

Boundary("end pottery currencies");

KDE_Plot("KDE_pottery", );

};

Sequence("metalwork currencies")

{

After("Bronze Age Period VI")

{

Date("end BA per.VI", Prior("end BA per.VI", "end_BA_perVI.prior"));

};

Boundary("start metalwork currencies");

Phase("metalwork currencies")

{

Sequence("pin w.type 1 coiled head")

{

Boundary("increase pin w.type 1 coiled head")

{

End("start of increase pin w.type 1 coiled head");

Transition("period of increase pin w.type 1 coiled head");

Start("end of increase pin w.type 1 coiled head");

};

Phase("pin w.type 1 coiled head")

{

Date("Aarupgaard U51_type 1", Prior("Aarupgaard U51", "Aarupgaard_U51.prior"));

Date("Aarupgaard U1186_type 1", Prior("Aarupgaard U1186", "Aarupgaard_U1186.prior"));

Date("Aarupgaard U1232_type 1", Prior("Aarupgaard U1232", "Aarupgaard_U1232.prior"));

Date("Aarupgaard U3452_type 1", Prior("Aarupgaard U3452", "Aarupgaard_U3452.prior"));

Date("Aarupgaard U3822_type 1", Prior("Aarupgaard U3822", "Aarupgaard_U3822.prior"));

Date("Aarre A89_type 1", Prior("Aarre A89", "Aarre_A89.prior"));

Date("Aarre A99_type1", Prior("Aarre A99", "Aarre_A99.prior"))-N(50,25);

Date("Aarre A393_type 1", Prior("Aarre A393", "Aarre_A393.prior"));

Date("Søhale x44_type 1", Prior("Søhale x44", "Sohale_x44.prior"));

Date("Søhale x47_type 1", Prior("Søhale x47", "Sohale_x47.prior"));

Date("Søhale x49_type 1", Prior("Søhale x49", "Sohale_x49.prior"));

Date("Søhale x51_type 1", Prior("Søhale x51", "Sohale_x51.prior"));

Date("Søhale x52_type 1", Prior("Søhale x52", "Sohale_x52.prior"));

Date("Søhale x65_type 1", Prior("Søhale x65", "Sohale_x65.prior"))-N(50,25);

Date("Søhale x74_type 1", Prior("Søhale x74", "Sohale_x74.prior"));

Date("Søhale x76_type 1", Prior("Søhale x76", "Sohale_x76.prior"));

Date("Søhale x92_type 1", Prior("Søhale x92", "Sohale_x92.prior"));

};

Span("duration pin w.type 1 coiled head");

Sum("pin w.type 1 coiled head (n=17)");

Boundary("decrease pin w.type 1 coiled head")

{

Start("start of decrease pin w.type 1 coiled head");

Transition("period of decrease pin w.type 1 coiled head");

End("end of decrease pin w.type 1 coiled head");

};

};

Sequence("pin w.type 2 coiled head")

{

Boundary("increase pin w.type 2 coiled head")

{

End("start of increase pin w.type 2 coiled head");

Transition("period of increase pin w.type 2 coiled head");

Start("end of increase pin w.type 2 coiled head");

};

Phase("pin w.type 2 coiled head")

{

Date("Aarupgaard U34_type 2", Prior("Aarupgaard U34", "Aarupgaard_U34.prior"));

Date("Aarupgaard U36_type 2", Prior("Aarupgaard U36", "Aarupgaard_U36.prior"));

Date("Aarupgaard U81_type 2", Prior("Aarupgaard U81", "Aarupgaard_U81.prior"));

Date("Aarupgaard U83_type 2", Prior("Aarupgaard U83", "Aarupgaard_U83.prior"));

Date("Aarupgaard U280_type 2", Prior("Aarupgaard U280", "Aarupgaard_U280.prior"));

Date("Aarupgaard U681_type 2", Prior("Aarupgaard U681", "Aarupgaard_U681.prior"));

Date("Aarupgaard U928_type 2", Prior("Aarupgaard U928", "Aarupgaard_U928.prior"))

-N(50,25);

Date("Aarupgaard U1279_type 2", Prior("Aarupgaard U1279", "Aarupgaard_U1279.prior"));

Date("Aarupgaard U3778_type 2", Prior("Aarupgaard U3778", "Aarupgaard_U3778.prior"));

Date("Aarre A86_type 2", Prior("Aarre A86", "Aarre_A86.prior"));

Date("Aarre A198_type 2", Prior("Aarre A198", "Aarre_A198.prior"));

Date("Aarre A278_type 2", Prior("Aarre A278", "Aarre_A278.prior"));

Date("Aarre A394_type 2", Prior("Aarre A394", "Aarre_A394.prior"));

Date("Søhale x44_type 2", Prior("Søhale x44", "Sohale_x44.prior"));

Date("Søhale x50_type 2", Prior("Søhale x50", "Sohale_x50.prior"));

Date("Søhale x72_type 2", Prior("Søhale x72", "Sohale_x72.prior"));

Date("Søhale x74_type 2", Prior("Søhale x74", "Sohale_x74.prior"));

Date("Søhale x76_type 2", Prior("Søhale x76", "Sohale_x76.prior"));

Date("Søhale x93_type 2", Prior("Søhale x93", "Sohale_x93.prior"));

};

Span("duration pin w.type 2 coiled head");

Sum("pin w.type 2 coiled head (n=19)");

Boundary("decrease pin w.type 2 coiled head")

{

Start("start of decrease pin w.type 2 coiled head");

Transition("period of decrease pin w.type 2 coiled head");

End("end of decrease pin w.type 2 coiled head");

};

};

Phase("bomb head pin")

{

Date("Aarupgaard U2710", Prior("Aarupgaard U2710", "Aarupgaard_U2710.prior"));

};

Sequence("pin w.circular head")

{

Boundary("increase pin w.circular head")

{

End("start of increase pin w.circular head");

Transition("period of increase pin w.circular head");

Start("end of increase pin w.circular head");

};

Phase("pin w.circular head")

{

Date("Aarupgaard U123_circular", Prior("Aarupgaard U123", "Aarupgaard_U123.prior"));

Date("Aarupgaard U183_circular", Prior("Aarupgaard U183", "Aarupgaard_U183.prior"))

-N(50,25);

Date("Aarupgaard U230_circular", Prior("Aarupgaard U230", "Aarupgaard_U230.prior"));

Date("Aarupgaard U293_circular", Prior("Aarupgaard U293", "Aarupgaard_U293.prior"));

Date("Aarupgaard U500_circular", Prior("Aarupgaard U500", "Aarupgaard_U500.prior"));

Date("Aarupgaard U752_circular", Prior("Aarupgaard U752", "Aarupgaard_U752.prior"));

Date("Aarupgaard U766_circular", Prior("Aarupgaard U766", "Aarupgaard_U766.prior"));

Date("Aarupgaard U797_circular", Prior("Aarupgaard U797", "Aarupgaard_U797.prior"));

Date("Aarupgaard U928_circular", Prior("Aarupgaard U928", "Aarupgaard_U928.prior"));

Date("Aarupgaard U1382_circular", Prior("Aarupgaard U1382", "Aarupgaard_U1382.prior"))-N(50,25);

Date("Aarupgaard U1617_circular", Prior("Aarupgaard U1617", "Aarupgaard_U1617.prior"))-N(50,25);

Date("Aarre A155_circular", Prior("Aarre A155", "Aarre_A155.prior"));

Date("Aarre A281_circular", Prior("Aarre A281", "Aarre_A281.prior"));

Date("Søhale x19_circular", Prior("Søhale x19", "Sohale_x19.prior"));

Date("Søhale x23_circular", Prior("Søhale x23", "Sohale_x23.prior"));

Date("Søhale x26_circular", Prior("Søhale x26", "Sohale_x26.prior"));

Date("Søhale x31_circular", Prior("Søhale x31", "Sohale_x31.prior"));

Date("Søhale x32_circular", Prior("Søhale x32", "Sohale_x32.prior"));

Date("Søhale x34_circular", Prior("Søhale x34", "Sohale_x34.prior"));

Date("Søhale x37_circular", Prior("Søhale x37", "Sohale_x37.prior"));

Date("Søhale x40_circular", Prior("Søhale x40", "Sohale_x40.prior"));

Date("Søhale x41_circular", Prior("Søhale x41", "Sohale_x41.prior"));

Date("Søhale x69_circular", Prior("Søhale x69", "Sohale_x69.prior"));

};

Span("duration pin w.circular head");

Sum("pin w.circular head (n=23)");

Boundary("decrease pin w.circular head")

{

Start("start of decrease pin w.circular head");

Transition("period of decrease pin w.circular head");

End("end of decrease pin w.circular head");

};

};

Sequence("simple iron ring")

{

Boundary("start simple iron ring");

Phase("simple iron ring")

{

Date("Aarupgaard U681_simple iron ring", Prior("Aarupgaard U681", "Aarupgaard_U681.prior"));

Date("Aarupgaard U1186_simple iron ring", Prior("Aarupgaard U1186", "Aarupgaard_U1186.prior"));

Date("Aarupgaard U2366_simple iron ring", Prior("Aarupgaard U2366", "Aarupgaard_U2366.prior"));

Date("Aarupgaard U2455_simple iron ring", Prior("Aarupgaard U2455", "Aarupgaard_U2455.prior"));

Date("Aarupgaard U3778_simple iron ring", Prior("Aarupgaard U3778", "Aarupgaard_U3778.prior"));

};

Span("duration simple iron ring");

Sum("simple iron ring (n=5)");

Boundary("end simple iron ring");

};

Phase("bronze neck ring")

{

Date("Aarupgaard U1076_bronze neck ring", Prior("Aarupgaard U1076", "Aarupgaard_U1076.prior"));

};

Phase("eye let ring")

{

Date("Søhale x30_eyelet", Prior("Søhale x30", "Sohale_x30.prior"));

Date("Søhale x65_eyelet", Prior("Søhale x65", "Sohale_x65.prior"));

};

Sequence("tongue-shaped belt clasp")

{

Boundary("start tongue-shaped belt clasp");

Phase("tongue-shaped belt clasp")

{

Date("Aarupgaard U183_tongue", Prior("Aarupgaard U183", "Aarupgaard_U183.prior"));

Date("Aarupgaard U346_tongue", Prior("Aarupgaard U346", "Aarupgaard_U346.prior"));

Date("Aarupgaard U1834_tongue", Prior("Aarupgaard U1834", "Aarupgaard_U1834.prior"));

Date("Aarre A130_tongue", Prior("Aarre A130", "Aarre_A130.prior"));

Date("Søhale x26_tongue", Prior("Søhale x26", "Sohale_x26.prior"));

Date("Søhale x28_tongue", Prior("Søhale x28", "Sohale_x28.prior"));

};

Span("duaration tongue-shaped belt clasp");

Sum("tongue-shaped belt clasp (n=6)");

Boundary("end tongue-shaped belt clasp");

};

Sequence("triangular belt clasp")

{

Boundary("increase triangular belt clasp")

{

End("start of increase triangular belt clasp");

Transition("period of increase triangular belt clasp");

Start("end of increase triangular belt clasp");

};

Phase("triangular belt clasp")

{

Date("Aarupgaard U123_triangular", Prior("Aarupgaard U123", "Aarupgaard_U123.prior"));

Date("Aarupgaard U427_triangular", Prior("Aarupgaard U427", "Aarupgaard_U427.prior"));

Date("Aarupgaard U1001_triangular", Prior("Aarupgaard U1001", "Aarupgaard_U1001.prior"));

Date("Aarupgaard U1363_triangular", Prior("Aarupgaard U1363", "Aarupgaard_U1363.prior"));

Date("Aarupgaard U1382_triangular", Prior("Aarupgaard U1382", "Aarupgaard_U1382.prior"));

Date("Aarupgaard U1617_triangular", Prior("Aarupgaard U1617", "Aarupgaard_U1617.prior"));

Date("Aarupgaard U1791_triangular", Prior("Aarupgaard U1791", "Aarupgaard_U1791.prior"));

Date("Aarupgaard U1847_triangular", Prior("Aarupgaard U1847", "Aarupgaard_U1847.prior"));

Date("Aarupgaard U1997_triangular", Prior("Aarupgaard U1997", "Aarupgaard_U1997.prior"));

Date("Søhale x30_triangular", Prior("Søhale x30", "Sohale_x30.prior"));

};

Span("duration triangular belt clasp");

Sum("triangular belt clasp (n=10)");

Boundary("decrease triangular belt clasp")

{

End("start of decrease triangular belt clasp");

Transition("period of decrease triangular belt clasp");

Start("end of decrease triangular belt clasp");

};

};

Sequence("iron ring w.shank")

{

Boundary("increase iron ring w.shank")

{

End("start of increase iron ring w.shank");

Transition("period of increase iron ring w.shank");

Start("end of increase iron ring w.shank");

};

Phase("iron ring w.shank")

{

Date("Aarupgaard U871_iron ring w.shank", Prior("Aarupgaard U871", "Aarupgaard_U871.prior"))

{

};

Date("Aarupgaard U1001_iron ring w.shank", Prior("Aarupgaard U1001", "Aarupgaard_U1001.prior"));

Date("Aarupgaard U1016_iron ring w.shank", Prior("Aarupgaard U1016", "Aarupgaard_U1016.prior"));

Date("Aarupgaard U1018_iron ring w.shank", Prior("Aarupgaard U1018", "Aarupgaard_U1018.prior"));

Date("Aarupgaard U1654_iron ring w.shank", Prior("Aarupgaard U1654", "Aarupgaard_U1654.prior"));

Date("Aarupgaard U1894_iron ring w.shank", Prior("Aarupgaard U1894", "Aarupgaard_U1894.prior"));

Date("Aarupgaard U1993_iron ring w.shank", Prior("Aarupgaard U1993", "Aarupgaard_U1993.prior"));

Date("Aarupgaard U2293_iron ring w.shank", Prior("Aarupgaard U2293", "Aarupgaard_U2293.prior"));

Date("Aarupgaard U2354_iron ring w.shank", Prior("Aarupgaard U2354", "Aarupgaard_U2354.prior"));

Date("Aarupgaard U2541_iron ring w.shank", Prior("Aarupgaard U2541", "Aarupgaard_U2541.prior"));

Date("Aarupgaard U2550_iron ring w.shank", Prior("Aarupgaard U2550", "Aarupgaard_U2550.prior"));

Date("Aarupgaard U2593_iron ring w.shank", Prior("Aarupgaard U2593", "Aarupgaard_U2593.prior"))

{

};

};

Span("duration iron ring w.shank");

Sum("iron ring w.shank (n=12)");

Boundary("decrease iron ring w.shank")

{

Start("start of decrease iron ring w.shank");

Transition("period of decrease iron ring w.shank");

End("end of decrease iron ring w.shank");

};

};

Sequence("narrow belt clasp")

{

Boundary("increase narrow belt clasp")

{

End("start of increase narrow belt clasp");

Transition("period of increase narrow belt clasp");

Start("end of increase narrow belt clasp");

};

Phase("narrow belt clasp")

{

Date("Aarupgaard U382_narrow", Prior("Aarupgaard U382", "Aarupgaard_U382.prior"));

Date("Aarupgaard U858_narrow", Prior("Aarupgaard U858", "Aarupgaard_U858.prior"));

Date("Aarupgaard U1076_narrow", Prior("Aarupgaard U1076", "Aarupgaard_U1076.prior"));

Date("Aarupgaard U1422_narrow", Prior("Aarupgaard U1422", "Aarupgaard_U1422.prior"));

Date("Aarupgaard U1436_narrow", Prior("Aarupgaard U1436", "Aarupgaard_U1436.prior"));

Date("Aarupgaard U1678_narrow", Prior("Aarupgaard U1678", "Aarupgaard_U1678.prior"));

Date("Aarupgaard U1970_narrow", Prior("Aarupgaard U1970", "Aarupgaard_U1970.prior"));

Date("Aarupgaard U2262_narrow", Prior("Aarupgaard U2262", "Aarupgaard_U2262.prior"));

Date("Søhale x18_narrow", Prior("Søhale x18", "Sohale_x18.prior"));

Date("Søhale x38_narrow", Prior("Søhale x38", "Sohale_x38.prior"));

Date("Søhale x41_narrow", Prior("Søhale x41", "Sohale_x41.prior"));

};

Span("duration narrow belt clasp");

Sum("narrow belt clasp (n=11)");

Boundary("decrease narrow belt clasp")

{

End("start of decrease narrow belt clasp");

Transition("period of decrease narrow belt clasp");

Start("end of decrease narrow belt clasp");

};

};

Sequence("pin w.rod-shaped head")

{

Boundary("start pin w.rod-shaped head");

Phase("pin w.rod-shaped head")

{

Date("Aarupgaard U858_rod-shaped head", Prior("Aarupgaard U858", "Aarupgaard_U858.prior"));

Date("Aarupgaard U871_rod-shaped head", Prior("Aarupgaard U871", "Aarupgaard_U871.prior"));

Date("Aarupgaard U1894_rod-shaped head", Prior("Aarupgaard U1894", "Aarupgaard_U1894.prior"));

Date("Aarupgaard U2293_rod-shaped head", Prior("Aarupgaard U2293", "Aarupgaard_U2293.prior"));

Date("Aarupgaard U2354_rod-shaped head", Prior("Aarupgaard U2354", "Aarupgaard_U2354.prior"));

Date("Aarupgaard U2366_rod-shaped head", Prior("Aarupgaard U2366", "Aarupgaard_U2366.prior"));

Date("Aarupgaard U2455_rod-shaped head", Prior("Aarupgaard U2455", "Aarupgaard_U2455.prior"));

Date("Aarupgaard U2545_rod-shaped head", Prior("Aarupgaard U2545", "Aarupgaard_U2545.prior"));

};

Span("duration pin w.rod-shaped head");

Sum("pin w.rod-shaped head(n=8)");

Boundary("end pin w.rod-shaped head");

};

Sequence("pin w.grooved head")

{

Boundary("start pin w.grooved head");

Phase("pin w.grooved head")

{

Date("Aarupgaard U1422_grooved head", Prior("Aarupgaard U1422", "Aarupgaard_U1422.prior"));

Date("Aarupgaard U1617_grooved head", Prior("Aarupgaard U1617", "Aarupgaard_U1617.prior"));

Date("Aarupgaard U1654_grooved head", Prior("Aarupgaard U1654", "Aarupgaard_U1654.prior"));

Date("Aarupgaard U1970_grooved head", Prior("Aarupgaard U1970", "Aarupgaard_U1970.prior"));

Date("Aarupgaard U1993_grooved head", Prior("Aarupgaard U1993", "Aarupgaard_U1993.prior"));

Date("Aarupgaard U1997_grooved head", Prior("Aarupgaard U1997", "Aarupgaard_U1997.prior"));

};

Span("duration pin w.grooved head");

Sum("pin w.grooved head(n=6)");

Boundary("end pin w.grooved head");

};

Sequence("Holstein pin")

{

Boundary("start Holstein pin");

Phase("Holstein pin")

{

Date("Aarupgaard U427_Holstein", Prior("Aarupgaard U427", "Aarupgaard_U427.prior"));

Date("Aarupgaard U2199_Holstein", Prior("Aarupgaard U2199", "Aarupgaard_U2199.prior"));

Date("Aarupgaard U2541_Holstein", Prior("Aarupgaard U2541", "Aarupgaard_U2541.prior"));

Date("Aarupgaard U2593_Holstein", Prior("Aarupgaard U2593", "Aarupgaard_U2593.prior"));

};

Span("duration Holstein pin");

Sum("Holstein pin (n=4)");

Boundary("end Holstein pin");

};

Sequence("winged head pin")

{

Boundary("start winged head pin");

Phase("winged head pin")

{

Date("Aarupgaard U867_winged", Prior("Aarupgaard U867", "Aarupgaard_U867.prior"));

Date("Aarupgaard U884_winged", Prior("Aarupgaard U884", "Aarupgaard_U884.prior"));

Date("Aarupgaard U1018_winged", Prior("Aarupgaard U1018", "Aarupgaard_U1018.prior"));

Date("Aarupgaard U2498_winged", Prior("Aarupgaard U2498", "Aarupgaard_U2498.prior"));

};

Span("duration winged head pin");

Sum("winged head pin(n=4)");

Boundary("end winged head pin");

};

};

Boundary("end metalwork currencies");

KDE_Plot("KDE_metalwork", );

};

Phase("order of introduction")

{

Sequence()

{

Date("=end of increase pin w.type 1 coiled head");

Date("=end of increase pin w.type 2 coiled head");

Date("=end of increase pin w.circular head");

Phase()

{

Date("=start pin w.grooved head");

Date("=start pin w.rod-shaped head");

};

Phase()

{

Date("=start Holstein pin");

Date("=start winged head pin");

};

};

Sequence()

{

Date("=start simple iron ring");

Phase()

{

Date("=start tongue-shaped belt clasp");

Date("=end of increase triangular belt clasp");

};

Phase()

{

Date("=end of increase iron ring w.shank");

Date("=end of increase narrow belt clasp");

};

};

};

Order("introduction of pottery")

{

Date("=start type 12B");

Date("=end of increase type 15B");

Date("=start type 20B");

Date("=start type 15C");

Date("=start type 18C");

Date("=start type 15D");

};

Phase("residence time")

{

Difference ("residence time_Aarre A99", "Aarre A99_burial", "Aarre A99_type1");

Difference ("residence time_Søhale x65", "Søhale x65_burial", "Søhale x65_type 1");

Difference ("residence time_Aarupgaard U928", "Aarupgaard U928_burial", "Aarupgaard U928_type 2");

Difference ("residence time_Aarupgaard U183", "Aarupgaard U183_burial", "Aarupgaard U183_circular");

Difference ("residence time_Aarupgaard U1382", "Aarupgaard U1382_burial", "Aarupgaard U1382_circular");

Difference ("residence time_Aarupgaard U1617", "Aarupgaard U1617_burial", "Aarupgaard U1617_circular");

Difference ("residence time_Aarupgaard U2366", "Aarupgaard U2366_burial", "Aarupgaard U2366_20B");

};

};

# References

1. Bronk Ramsey C. Bayesian Analysis of Radiocarbon Dates. Radiocarbon. 2009; 51(1): 337-60. <https://doi.org/10.1017/S0033822200033865>
